# Photooxidation of Dipyrrinones: Reaction with Singlet Oxygen and Characterization of Reaction Intermediates

**DOI:** 10.1021/acs.joc.4c02954

**Published:** 2025-02-05

**Authors:** Dominik Madea, Júlia Peňáková, Jaya Mehara, Rikuo Akisaka, Marek Martínek, Jana Roithová, Petr Klán

**Affiliations:** † Department of Chemistry, Faculty of Science, Masaryk University, Kamenice 5, Brno 625 00, Czech Republic; ‡ RECETOX, Faculty of Science, Masaryk University, Kamenice 5, Brno 625 00, Czech Republic; § Institute for Molecules and Materials, Faculty of Science, Radboud University, Heyendaalseweg 135, Nijmegen 6525 AJ, Netherlands

## Abstract

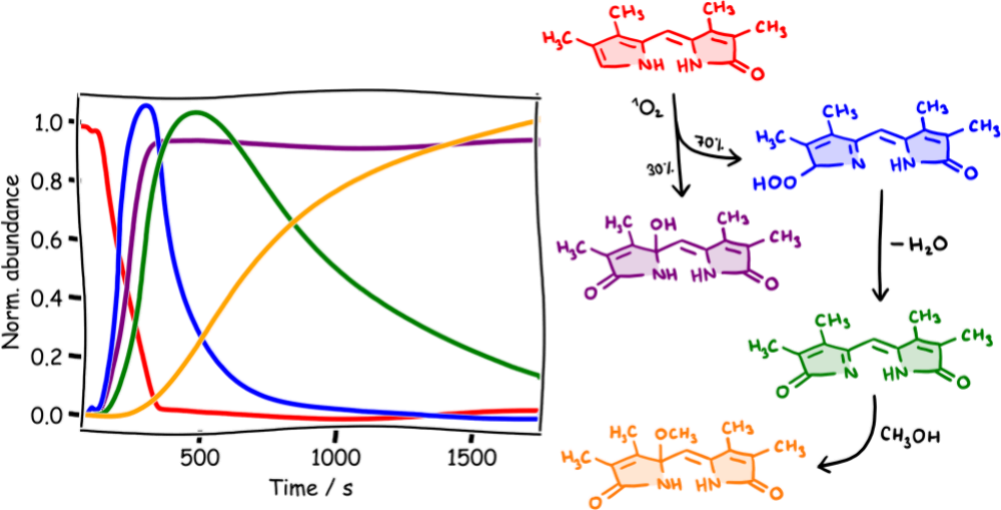

Bilirubin (BR) is a water-insoluble product of heme catabolism in mammals. Elevated blood concentrations of BR, especially in the neonatal period, are treated with blue-green light phototherapy. The major mechanism of BR elimination during phototherapy is photoisomerization, while a minor, less studied mechanism of degradation is oxidation. In this work, we studied the oxidation of the bilirubin model tetramethyl-dipyrrinone (*Z*-**13**) by singlet oxygen in methanol using UV–vis and ESI-MS spectroscopy, resulting in propentdyopents as the main oxidation products. We also identified two additional intermediates that were formed during the reaction (hydroperoxide **21a** and imine **17**). The structure of the hydroperoxide was confirmed by helium-tagging IR spectroscopy. Such reaction intermediates formed during the oxidation of BR or bilirubin models have not been described so far. We believe that this work can be used as a first step in studying the complex oxidation mechanism of BR during phototherapy.

## Introduction

**Figure 1:**
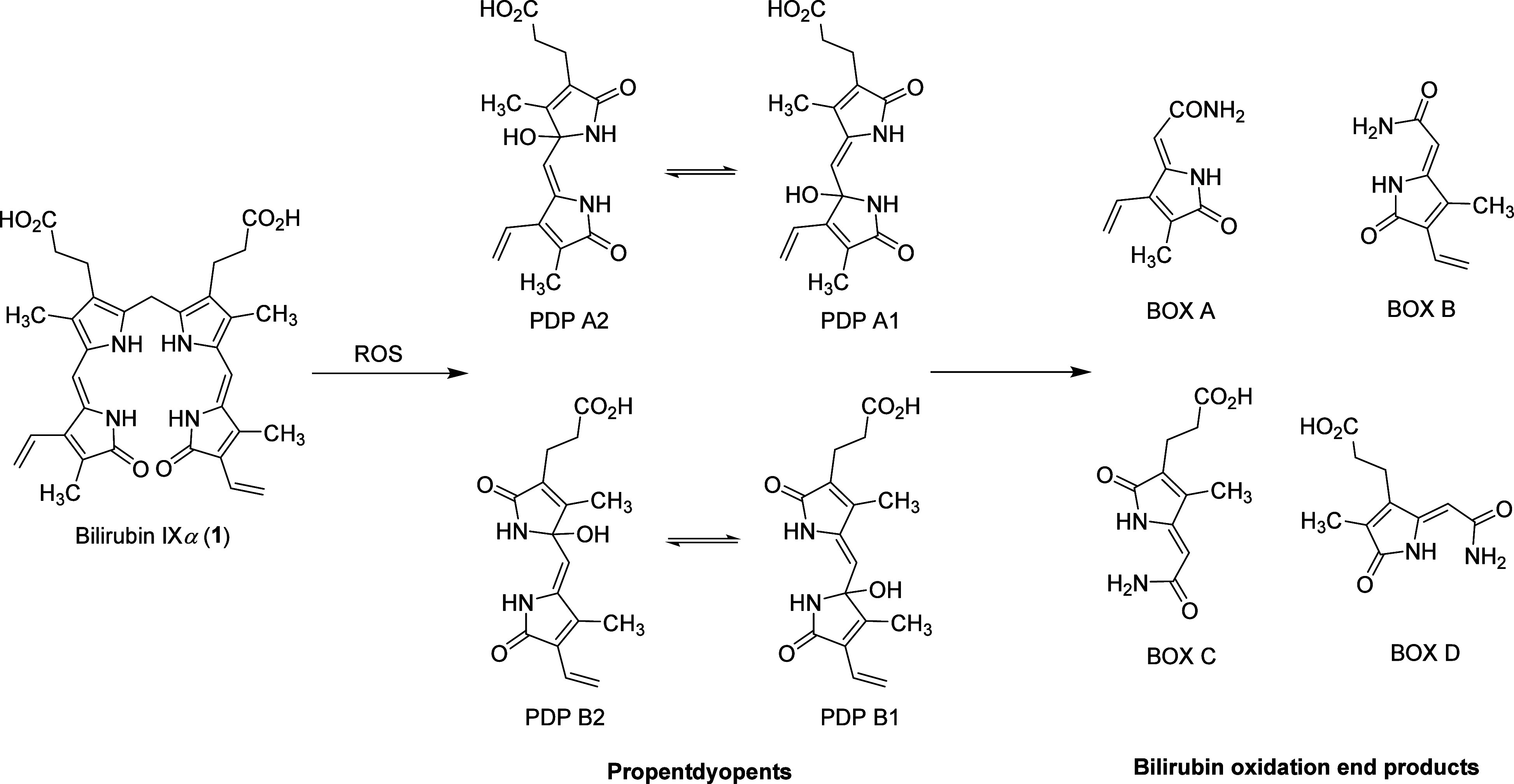
Bilirubin and its oxidation products.^9^

Bilirubin IXα (BR, **1,**Figure 1) is a water-insoluble product of heme catabolism in mammals. Its yellow coloration is noticeable in jaundiced patients or when experiencing bruising. Bilirubin serves as a powerful antioxidant and, in small amounts, exhibits anti-inflammatory properties that are correlated with a reduced incidence of chronic, especially cardiovascular, diseases.^1^ In addition, bilirubin is now recognized as an important signaling molecule that acts through modulation of specific receptors.^2^ However, elevated levels of bilirubin in the bloodstream lead to jaundice, a potentially life-threatening condition, especially in newborns, because bilirubin is neurotoxic.^3^ Phototherapy using blue or turquoise light is a widely accepted treatment for neonatal jaundice.^4−6^ When exposed to light, bilirubin in tissues undergoes configurational and structural isomerization and is converted into forms more soluble in water and easily excreted.^7,8^

In addition to the natural elimination of bilirubin through glucuronidation in healthy adults, bilirubin undergoes nonenzymatic degradation by reactive oxygen species (ROS), particularly during oxidative stress.^10^ This process generates products, such as dipyrrolic propentdyopents (PDPs, Figure 1)^11^ and monopyrrolic bilirubin oxidation end products (BOXes, Figure 1).^12,13^ BOXes play a significant role in the development of cerebral vasospasms, often observed after complications from subarachnoid hemorrhage.^14^

A minor pathway involved in the degradation of bilirubin during phototherapy is photooxidation.^15^ The products of *in vivo* and *in vitro* self-sensitized bilirubin photooxidation, which is induced by direct irradiation of bilirubin, as well as dye-sensitized photooxidation, are primarily propentdyopents and maleimide derivatives such as hematinic acid and methylvinylmaleimide, along with some other compounds.^15−20^ Possible photooxidation mechanisms have been extensively discussed in the literature, with both Type I and Type II mechanisms proposed.^21^ The Type I mechanism^22^ involves electron transfer (eT) from the excited state of bilirubin (singlet and/or triplet state) to ground state oxygen (^3^O_2_), forming an ion pair of the bilirubin radical cation (BR^•+^) and superoxide (O_2_^•–^), which then undergo further reactions to yield various oxidation products. On the other hand, the Type II mechanism^22^ involves the production of singlet oxygen (^1^O_2_) via triplet–triplet annihilation of the triplet state of bilirubin and ^3^O_2_. Singlet oxygen subsequently reacts with bilirubin either directly or via an eT mechanism.^21^ It is widely accepted that bilirubin reacts through both Type I and Type II mechanisms, as various experimental evidence supports either mechanism.^21^ Interestingly, bilirubin exhibits chemiluminescence in the presence of various oxidation agents (NaClO, *N*-bromosuccinimide, peroxonitrite)^23,24^ or even under aerobic conditions in strongly alkaline aqueous solutions.^25,26^ Hydroperoxide and/or hydroxide intermediates were thought to be responsible for the observed chemiluminescence.^25,26^

**Scheme 1:**
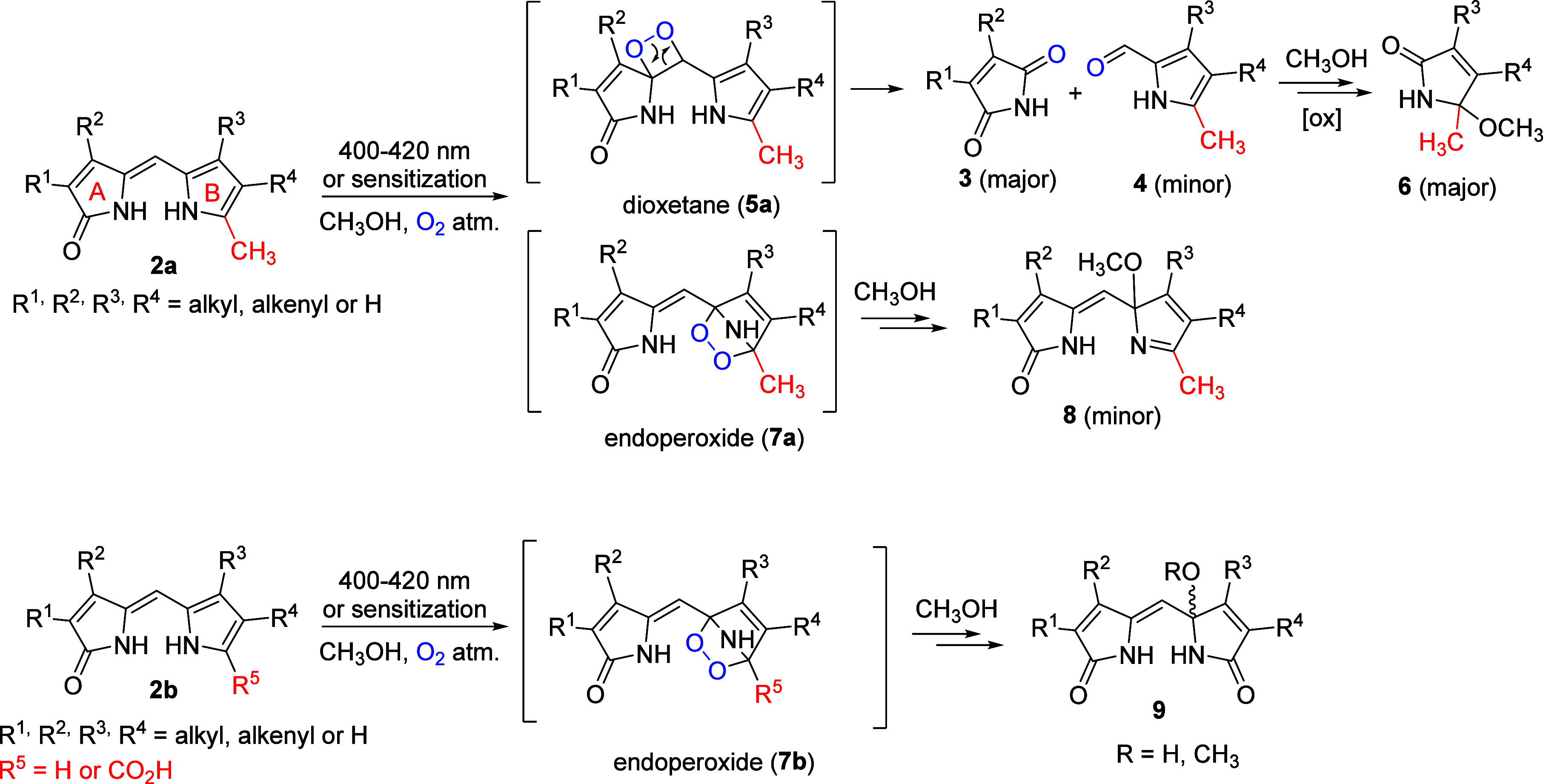
Reaction Products of Photooxidation of Dipyrrinone Models for BR^21,35,36^^,^ Information in the parentheses refers to isolated yields.

Because of the intricate structure of bilirubin, researchers have been investigating simplified versions of bilirubin, dipyrrinones (**2a**,**b,**Scheme 1).^19,21,27−36^ The structures of the products resulting from self-sensitized (under direct irradiation) and dye-sensitized photooxidation of dipyrrinone models (Scheme 1) in methanol are strongly affected by the presence of a 2-methyl group of the pyrrole moiety of dipyrrinone (Scheme 1). Dipyrrinones possessing this methyl group (**2a**) give maleimide **3** and pyrrolecarbaldehyde **4** as major primary photoproducts. Maleimide is exclusively derived from ring A of dipyrrinone **2a**, while the aldehyde originates from ring B, as demonstrated for dipyrrinones with different substituents on both rings.^21,28,31,33^ It has been suggested that these products probably originate from the dioxetane intermediate **5a** (Scheme 1), formed at some stage of the reaction by a Type I or Type II process.^21,28,33,34^ Other isolated products include 5-methyl-5-methoxy-pyrrolin-2-one (**6**), which was shown to be produced by further oxidation of **4,**(37) and **8** (Scheme 1), which was proposed to originate from the endoperoxide intermediate **7a** (Scheme 1).^21^ Propentdyopents, observed during the photooxidation of bilirubin, were not detected in this process.^21,28,31,33^ However, the scenario is different for unsubstituted dipyrrinones or those containing a carboxylic group at the 2 position of the pyrrole group (**2b**). The main products of photooxidation of **2b** are propentdyopents **9** (Scheme 1), probably originating from the endoperoxide intermediate **7b**.^9,35,36^

**Figure 2:**
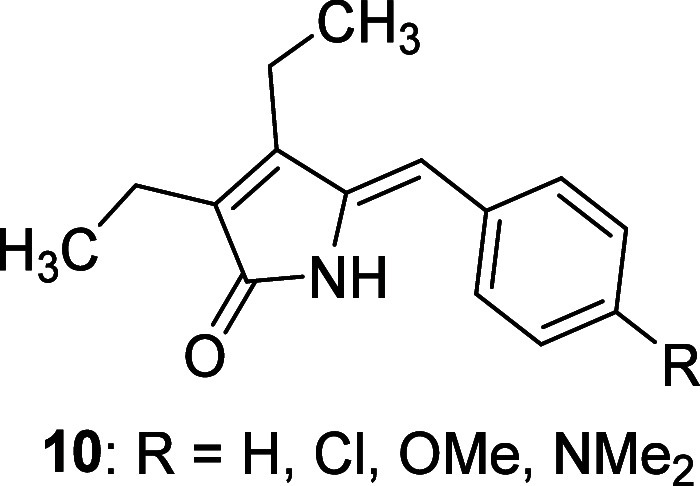
Benzalpyrrolinones.

The formation of primary products **3** and **4** from 2-methyldipyrrinones was initially explained as the addition of singlet oxygen to the ene-amide group of **2a**, forming the dioxetane intermediate **5a**.^28^ This assumption was based on the known reactivity of enamines toward ^1^O_2_, resulting in analogous products.^38^ Later, several benzalpyrrolinones (**10**, Figure 2) were synthesized and subjected to self- and dye-sensitized reactions.^39^ However, in addition to demonstrating configurational isomerization, they remained stable during photooxidation. Moreover, introducing electron-donating groups on benzalpyrrolinones (**10**, Figure 2) to mimic the effect of the pyrrole ring in dipyrrinones had no impact on reactivity.^39^ Thus, the formation mechanism of products **3** and **4** remains unclear because the ene-amide group itself does not exhibit the “unique” reactivity as observed in **2a**. The distinctive reactivity observed between 2-methyl and unsubstituted (or carboxylic group-containing) dipyrrinones thus remains unexplained. The kinetics of bilirubin photooxidation or its subunit models have not been comprehensively investigated using mass spectrometry (MS) methods. Only UV–vis kinetic studies have been conducted for 2-substituted dipyrrinone models, including xanthobilirubic acid.^21,30^

**Scheme 2:**
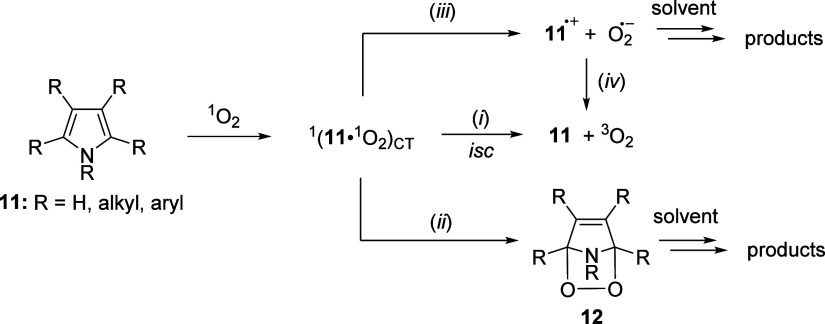
Simplified Scheme Illustrating Quenching of ^1^O_2_ by Pyrrole Derivatives

Compared to ground-state oxygen (*E*_red_ = −0.35 V vs NHE),^40^ singlet oxygen (*E*_red_ = 0.64 V vs NHE)^40^ is a much stronger oxidant. It is generally accepted that when ^1^O_2_ interacts with a quencher that has a sufficiently low oxidation potential (*E*_ox_ < 1.9 V vs SCE) and a high triplet energy (*E*_T_ > 0.97 eV), ^1^O_2_ is deactivated through the formation of a charge-transfer complex (exciplex)^41^ (see Scheme 2 in which substituted pyrrole **11** is explicitly used as a quencher). This was rationalized by correlating the total quenching rate constant with the free Gibbs energy associated with complete eT for different quenchers.^41^ This exciplex can decay by physical quenching to give ground state oxygen (*i*), chemical reaction (*ii*), or full eT to form the pyrrole cation radical (**11**^•+^) and superoxide (O_2_^•–^) (*iii*, Scheme 2). Free ions can undergo back eT or form reaction products, either in the presence or absence of a solvent. Superoxide was detected directly (EPR)^42^ or indirectly (by reduction of 1,4-benzoquinone^43^ or NBT,^44,45^ disproportionation by superoxide dismutase,^44,45^ or monitoring reaction products^46,47^) in scenarios where strong electron donors and/or protic solvents such as water or methanol are used, which facilitate the dissociation of the exciplex to free ions via a radical pair mechanism. Furthermore, it was observed that singlet oxygen can also undergo hydrogen atom transfer (HAT) from some tertiary aliphatic amines in aprotic solvents.^47−51^

The main reaction of pyrroles with ^1^O_2_ is believed to be a spin-allowed Diels–Alder [4 + 2] addition (pathway *ii*, Scheme 2), leading to the formation of endoperoxide **12** (Scheme 2).^52,53^ According to density functional theory (DFT) calculations, this route shows the lowest activation energy compared to those of alternative mechanisms such as [2 + 2] addition and two-step *ene*-reaction.^54−56^ However, certain electron-rich pyrroles predominantly give products arising from [2 + 2] addition.^57,58^ The endoperoxide intermediate was observed only by low-temperature ^1^H NMR, and it was argued that the [2 + 2] product could be formed by its rearrangement.^57,59^ The reaction products obtained from the addition of ^1^O_2_ to 2,5-disubstituted pyrroles exhibit notable differences compared to unsubstituted pyrroles at one of these positions.^60−62^ These products pose challenges in terms of explaining their origin from the initial endoperoxide. Orfanopoulos and co-workers proposed an eT mechanism as a possible explanation.^62^ The hypothesis was supported by the fact that a radical scavenger inhibits the reaction.^62^ Finally, the different pathways (*ii* and *iii,*Scheme 2) may afford identical reaction products, as experimentally observed for a tryptophan derivative.^63^ Specifically for dipyrrinones and BR, it was suggested multiple times^21,28,32,33,64^ that the interaction of singlet oxygen with these compounds may involve eT, leading to superoxide and the corresponding radical cation. However, to the best of our knowledge, no experiments directly detecting superoxide were carried out to validate this hypothesis.

In our previous study, we observed and isolated propentdyopents by dye-sensitized photooxidation and direct irradiation of the bilirubin subunit *Z*-isovinylneoxanthobilirubic acid methyl ester.^36^ Propentdyopents originating from the second part of BR were also obtained by sensitized photooxidation of *Z*-vinylneoxanthobilirubic acid methyl ester containing a carboxylic group at the 2 position.^9^ Due to the unresolved mysteries surrounding the photooxidation processes, we were motivated in this work to investigate this mechanism in more detail. We chose simplified dipyrrinones analogous to those previously studied but with the key positions substituted with methyl groups. This modification ensured the formation of symmetrical propentdyopents. Similar to our previous reports, we studied the reactivity of dipyrrinones mainly in methanol, which dissolves our substrates while mimicking the aqueous environment during phototherapy. Here, we present a mechanistic investigation of the reactivity of *Z*-**13** with ^1^O_2_ using UV–vis and MS kinetic experiments in conjunction with infrared detection of reaction intermediates. We focus exclusively on its oxidation by singlet oxygen utilizing both dye-sensitized and thermally generated ^1^O_2_. In future reports, we will delve into the mechanism of photooxidation under direct irradiation conditions.

## Results and Discussion

### Synthesis

**Scheme 3:**
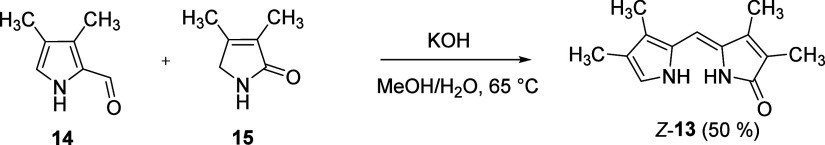
Synthesis of *Z*-**3**

**Scheme 4:**
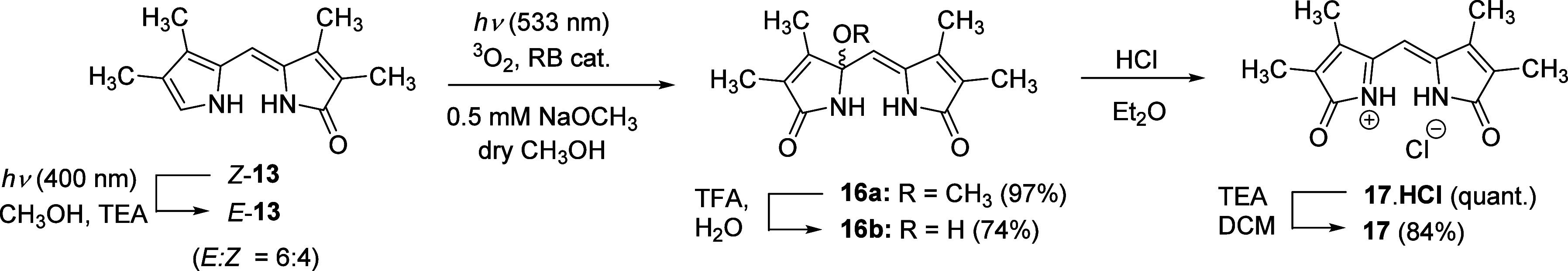
Synthesis of Propentdyopents, Iminium Salt **17**·**HCl**, and the Corresponding Imine **17**

The synthesis of model BR subunit *Z*-**13** was accomplished by condensation of aldehyde **14** and pyrrolone **15** (Scheme 3) under basic conditions in moderate yield (50%).^65,66^ The building blocks **14** and **15** were prepared from 3,4-dimethylpyrrole^67^ according to the literature.^66^ The (*E*)-isomer of **13** was obtained by irradiation of *Z*-**13** in methanol using 400 nm light to obtain a ∼ 1:1 mixture of the configurational isomers of **13**, from which *E*-**13** was isolated by silica-gel column chromatography (see configurational isomerization quantum yields on page S2). The methanol adduct of propentdyopent (**16a**, Scheme 4) was prepared in almost quantitative yield using rose bengal (RB)-sensitized oxidation of *Z*-**13** according to our previously reported method.^36^ The corresponding water adduct of propentdyopent (**16b**) was prepared in good yield by treatment of **16a** with trifluoroacetic acid, followed by the addition of water. Iminium salt **17·HCl** was obtained quantitatively by treating **16a** with anhydrous HCl in dry diethyl ether, following a modified literature procedure.^35^ The corresponding freebase **17** was obtained by the reaction of **17·HCl** with triethylamine in dichloromethane in good yields.

### Photophysical Properties

**Figure 3:**
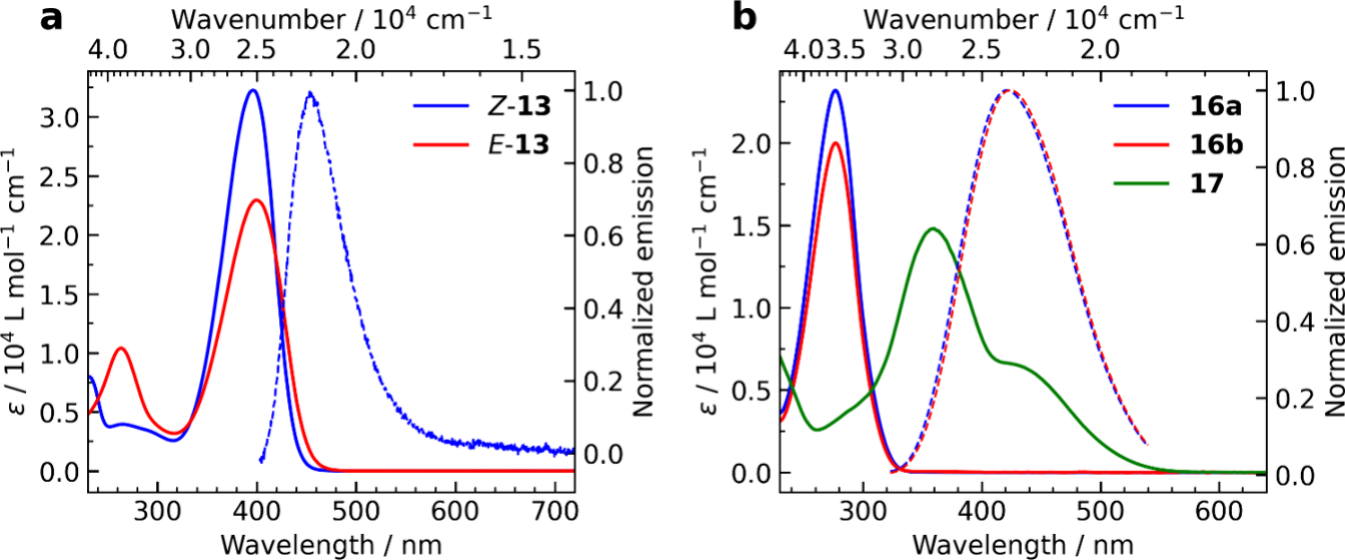
Molar absorption coefficients (solid lines) and fluorescence spectra (dashed lines) of (a) *Z*-**13** and *E*-**13**, and (b) major oxidation products (*vide infra*) obtained in methanol (absorption spectrum of **17** was recorded in dichloromethane). The absorption spectrum of *E*-**13** and emission spectra of **16a** and **16b** were extracted from the HPLC data.

The absorption and emission bands of *Z*-**13** and *E*-**13** in methanol (Figure 3a, Table 1) are hypsochromically shifted compared to those of dipyrrinones that bear π-extending vinyl substituents.^36,68^ Fluorescence of the isomers **13** is very weak and has not been explicitly determined, analogous to those of the bilirubin subunits previously reported.^36,68^*E*-**13** is less fluorescent than *Z*-**13**, indicating a shorter lifetime of the singlet excited state than that of *Z*-**13**, which is in agreement with our previous results.^36^ We did not observe phosphorescence from *Z*-**13** in frozen methanol at 77 K (see Figure S27); the singlet excited state of *Z*-**13** either does not undergo intersystem crossing to the triplet state or its formation is very inefficient. Spectra of propentdyopents **16** in methanol (Figure 3b, Table 1) possess the main absorption peak at 277 nm, which is slightly hypsochromically shifted compared to those of propentdyopents formed by oxidative degradation of bilirubin (λ_max_ = 286 and 296 nm).^36,69^ The absorption spectrum of imine **17** (λ_max_ = 356 nm, Figure 3b, Table 1) is broad and extends over 500 nm. We did not detect any fluorescence from **17** or its salt **17·HCl** in aprotic solvents (CH_2_Cl_2_).

**Table 1:** Photophysical Properties of *Z*-**13** and *E*-**13** and Their Major Photooxidation Products in Methanol^k^

compd	λ_max_(abs)/nm^a^	ε(λ_max_)/10^4^ L mol^–1^ cm^–1^	λ_max_(em)/nm^b^*^,^*^c^	Φ_F_^c^
*Z*-**13**	396	3.23, (2.72),^70^ (2.67 in CH_3_CN),^70^ (3.26),^65^ (3.36)^71^	454	n.d.^e^
*E*-**13**	400	2.30	n.d.^d^	n.d.^e^
**16a**	277, (274)^35^	2.32, (2.01)^35^	421^f^	<0.01
**16b**	277, (273)^72^	2.00, (2.57)^72^	424^f^	<0.01
**17**^g^	360,^g^ 356^h^	1.48^g^	-i	-i
**17·HCl**^j^	400,^j^ (402)^35^	n.d.,^e^ (0.8)^35^	-i	-i

a Absorption maxima.

b Emission maxima; λ_ex_ = 390 nm for *Z***-13**, 290 nm for **16a,b**.

c Determined in solutions with *A*(λ_ex_) < 0.1.

d The fluorescence spectrum could not be recorded due to the presence of the more strongly fluorescent *Z*-**13**. Weak emission could not be extracted from the HPLC emission data.

e n.d. = not determined.

f This value was obtained from HPLC data.

g In dichloromethane.

h In methanol.

i No fluorescence was detected.

j In CF_3_COOH.

k Values in parentheses were taken from the literature.

### Products of Photooxidation and Photodecomposition of **13**

Before studying the reaction mechanism, it was necessary to characterize and quantify the photoproducts generated by direct irradiation of **13** as well as those formed by the reaction of ^1^O_2_ with **13** under different conditions (solvent, temperature, and the presence of different additives such as acid or base). ^1^O_2_ can be generated either by sensitization or thermal reaction, and we employed both methods. RB or methylene blue (MB) served as sensitizers, while 1,4-dimethylnaphthalene endoperoxide (**DMNO**_**2**_) was used as a source of ^1^O_2_ formed thermally.^73^ The use of sensitizers may introduce additional potential photoproducts,^62^ as their excited triplet state can act as an energy donor for *Z*-**13** or participate in electron transfer reactions.^74^ We observed sensitized isomerization of *Z*-**13** upon irradiation of a mixture of *Z*-**13** and RB at 532 nm in methanol under deoxygenated conditions (Ar-purged solution). Park and co-workers also observed this phenomenon on a similar dipyrrinone.^31^ To mitigate these undesired reactions, we used low concentrations of sensitizers (5–10 mol % of the *Z*-**13** concentration) and purged the solution with oxygen before irradiation to maximize singlet oxygen production. HPLC coupled with ESI-MS was used to analyze the reaction mixtures (Supporting Information), and the results are summarized in Table 2.

**Table 2:**
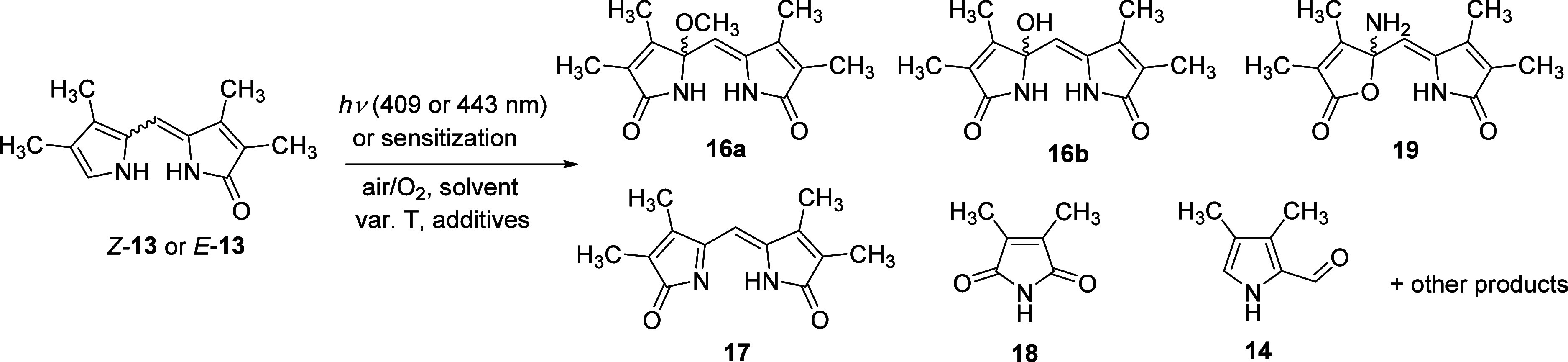
Photoproducts Formed by the Oxidative Degradation of Isomers of **13** in Oxygenated Methanol and Acetonitrile^a^

entry	solvent	*T*/°C	conditions^b^	**16a**^c^	**16b**^c^	**17**^c^	**18**^c^	**19**^c^	**14**^c^	others^d^
1	CH_3_OH^e^	22	direct irr. (409 nm)	51.5	30.2	-	6.3	7.9	2.3	1.7
2	CH_3_OH^f^	22	direct irr. (443 nm)	35.7	32.5	-	7.2	14.0	-	10.6
3	CH_3_OH	22	RB	40.2	29.3	-	2.1	14.2	-	14.2
4	CH_3_OH	22	MB	56.3	20.7	-	3.0	9.6	-	10.4
5	CH_3_OH (with TFA)	22	MB	80.8	-	-	3.5	9.0	-	6.7
6	CH_3_OH	–78	RB	33.0	26.2	-	1.6	19.3	-	19.9
7	CH_3_OH (with NaOCH_3_)	22	RB	95.7	0.6	-	3.7	-	-	0.0
8	CH_3_OH (*E*-**13**)	22	MB	42.4	29.1	-	1.8	12.3	-	14.4
9	CH_3_OH	22	DMNO_2_	56.5	17.3	-	5.3	8.8	-	12.1
10a^g^	CH_3_CN^h^	22	RB	-	24.8	24.2	5.6	30.8	-	9.9
10b^g^	CH_3_CN^i^	22	RB	-	24.8	26.6	10.1	29.9	-	8.6

a *Z*-**13** was used as the starting material in all experiments except entry 8, where *E*-**13** was used.

b Direct irr. (λ/nm) is direct irradiation at a specified wavelength; in other cases, sensitizers, such as rose bengal (RB) or methylene blue (MB), or a singlet oxygen generator 1,4-dimethylnaphthalene endoperoxide (**DMNO**_**2**_) were used.

c Chemical yields estimated from the HPLC data (UV–vis detector; molar absorption coefficients were not considered).

d Estimated total chemical yields of other unidentified photoproducts (see extended Table S2, Figure S39b and Experimental Part for details).

e An aerated sample was irradiated at 409 nm for 42 h at room temperature (Figure S36).

f An oxygenated sample was irradiated at 443 nm for 225 min at room temperature (Figure S37).

g These entries relate to a single experiment conducted with two distinct workup procedures.

h After the reaction was completed, methanol was added, and the solution was incubated for 2 h at room temperature to convert the imine **17** to **16a**. The yield of **16a** was then used to calculate the yield of **17**.

i Same as in the case *h*, but excess water was added, and the yield of **17** was calculated based on the yield of **16b** (entries 10a,b; HPLC chromatograms are shown in Figure S34).

Upon direct irradiation (λ_irr_ = 443 nm) of **13** in methanol under an O_2_ atmosphere at room temperature, the main photoproducts were propentdyopents **16** (Figure S37). Propentdyopent **16b** is stable in neutral methanol and does not convert to **16a**. Such a reaction requires acid catalysis (see entry 5, Table 2).^9,36^ Maleimide **18** and amine **19** (entry 2, Table 2) were also detected along with some other minor products that could not be isolated, probably due to their instability (see the Supplementary discussion in page S43 for more details). Maleimides are commonly observed during the oxidations of pyrrole^52,62,75^ as well as bilirubin.^15,17,19,20^ The structure of **19** was confirmed by 2D NMR and HPLC-MS experiments (Figures S15–S20, S35). **19** was independently prepared by oxidation of *Z*-**13** with *m*-CPBA in dichloromethane at −15 °C, followed by exhaustive separation by reverse phase flash chromatography. To the best of our knowledge, such rearrangement products have never been reported during the photooxidation of pyrroles. Imine **17** was not detected (Table 2, entry 2); however, it was found that **17** is produced as an intermediate during the reaction. In methanol, **17** is quantitatively converted to **16a** with a lifetime of 5.6 min at room temperature (see Figure S28), and the reaction is accelerated in the presence of either acid or base. Similar product ratios were observed under sensitized conditions (Table 2, entries 3 and 4) and using **DMNO**_**2**_ (Table 2, entry 9) as the source of ^1^O_2_. The similar composition of photoproducts in these experiments suggests that the sensitizer does not exhibit any other type of reactivity under the conditions used, apart from the production of singlet oxygen. This indicates that the photosensitizer in unlikely to be involved in eT reactions that are sometimes observed during pyrrole photooxidation.^62^ Additionally, sensitization of *E*-**13** (entry 8, Table 2) resulted in the same products as observed for sensitization of *Z*-**13**. This suggests that the configuration of the internal double bond of **13** does not significantly affect the reaction mechanism. Aldehyde **14** was observed only in small amounts upon direct irradiation at 409 nm in aerated samples (entry 1, Table 2, Figure S36). We believe that this product was formed as a secondary photoproduct of photooxidation/photodegradation of unidentified primary photoproducts (Figure S39b). We did not detect **14** upon irradiation at 443 nm (entry 2, Table 2, Figure S37), where these unidentified photoproducts do not absorb (Figure S39b).

The major products obtained under photosensitized conditions in dry acetonitrile (Table 2, entry 10a) were imine **17**, water adduct **16b**, and amine **19**, along with small amounts of **18** and other products (Figures S33 and S34, see UV–vis kinetics in Figure S45). To quantify the amount of **17**, which cannot be directly observed in HPLC because it reacts with the used mobile phase (water and methanol), we incubated the fresh photolysate with excess methanol to quantitatively convert **17** to **16a**, and the amount of **17** was calculated from the signal of **16a**. To evaluate whether the addition of methanol as a nucleophile would induce any additional thermal reactions besides the conversion of **17** to **16a**, methanol was replaced by water (Table 2, entry 10b, Figure S34). The addition of water caused quantitative conversion of **17** to water adduct **16b**. In both experiments, the product distributions of the main products were comparable (entries 10a, b, Table 2). In addition to isomerization, very inefficient degradation was observed in methanol and acetonitrile upon direct irradiation under degassed conditions (achieved by three freeze–pump–thaw cycles). The products did not absorb significantly in the detectable spectral range (200–400 nm) and were consequently not characterized.

In nonpolar solvents (toluene and hexane), we observed the formation of covalently bound dimers of *Z*-**13** upon irradiation (HPLC with ESI-MS, UV–vis, Figure S29–S31). These dimers decomposed upon exhaustive irradiation. It is evident that the photochemistry of **13** in nonpolar solvents differs from that in polar solvents. The main reason appears to be the self-association of **13**. For instance, some substituted dipyrrinones in CDCl_3_ self-dimerize with moderate association constants of ∼10^4^ M^–1^.^76^ Aggregation of *Z*-**13** in toluene was spectroscopically apparent from concentration-dependent plots (Figure S30). However, a similar dependence in polar solvents (methanol and acetonitrile) did not result in any change in the spectra at the studied concentrations (<5 × 10^–4^ M; Figure S46). We attribute the spectral changes observed in toluene to dimerization due the known behavior of dipyrrinones. We assume that self-association will not be relevant in biological conditions; therefore, the photochemistry of *Z*-**13** in nonpolar solvents was not further studied.

### Reaction Kinetics of Dye-Sensitized Photooxidation of *Z*-**13** in Methanol

**Figure 4:**
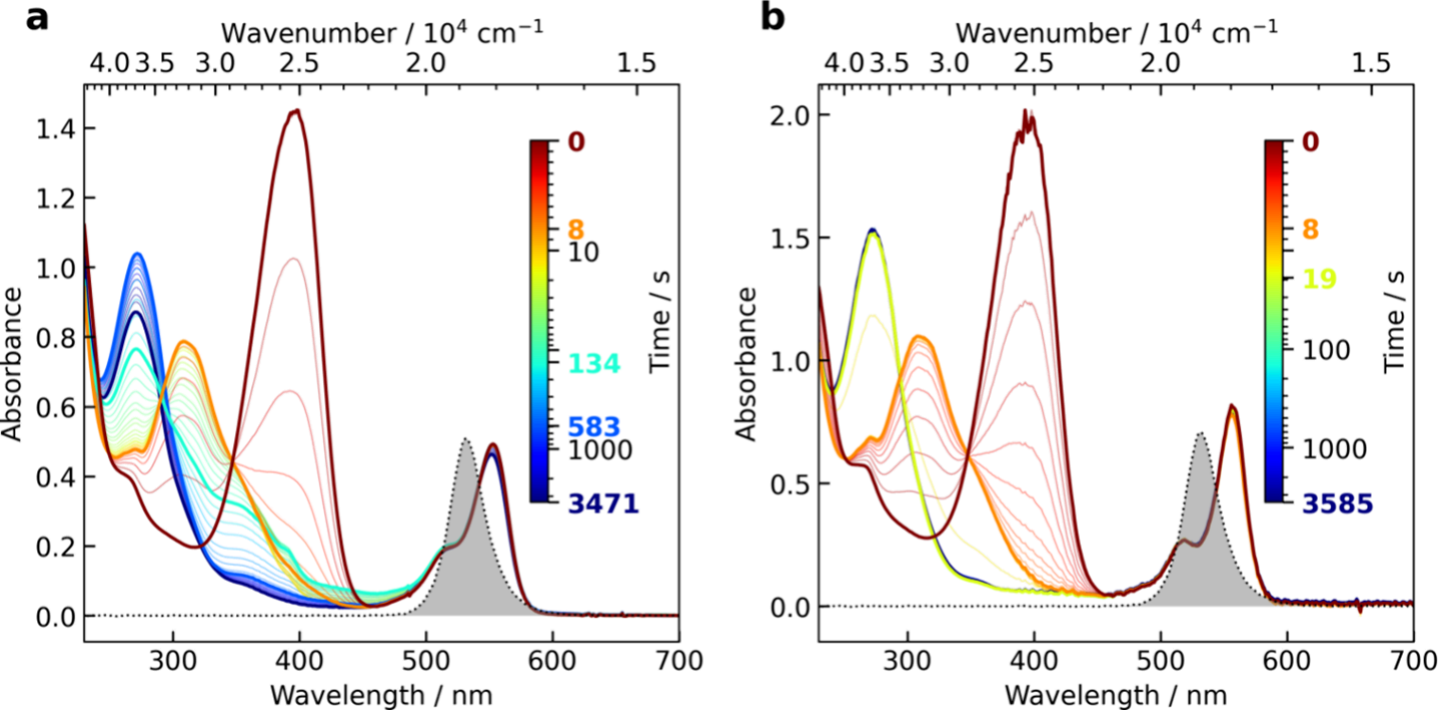
RB-sensitized oxidation of *Z*-**13** in methanol under an oxygen atmosphere. Irradiation was carried out at 532 nm (the spectrum of LEDs is shown as a gray area). (a) After the formation of the intermediate at ∼8 s, the LED irradiation was turned off. (b) An analogous experiment with adding NaOCH_3_ after 8 s (50 μL of 0.5 mol L^–1^ solution in methanol, cuvette volume of ∼2.5 mL).

To understand the evolution of the reaction products and possible intermediates such as imine **17**, we studied the reaction kinetics of *Z*-**13** with ^1^O_2_ using UV–vis spectroscopy and ESI-MS. First, we performed the RB-sensitized oxidation of *Z*-**13** (*c*_*Z*-**13**_ = 43 μmol L^–1^, *c*_RB_ = 6.2 μmol L^–1^) in methanol under an oxygen atmosphere (Figure 4a). The sample was irradiated with 532 nm LEDs (Figure S64) until the absorption band corresponding to *Z*-**13** at ∼400 nm (Figure 4a) disappeared. The process was very fast (8 s), and a new band corresponding to an intermediate absorbing at ∼310 nm (orange line, Figure 4a) appeared. Then, the LED was turned off, and the sample composition in the dark was evaluated. The intermediate (λ_max_ ∼ 310 nm) disappeared in ∼3 min, and new species absorbing at λ_max_ ∼ 277 and ∼360 nm (cyan line at 134 s, Figure 4a) were detected. The band at ∼277 nm was attributed to propentdyopent-like compounds such as **16** and **19** (Figure 4b and Table 1), and the signal at ∼360 nm was assigned to imine **17**. During the additional ∼10 min, the ∼360 nm band disappeared, and that at ∼277 nm band was enhanced (blue line at 1362 s, Figure 4a). The observed kinetics was not significantly different from that when the LED irradiation was maintained throughout (see Figure S41), suggesting that the intermediates formed are not significantly reactive toward singlet oxygen.

Previous experiments have shown that compound **17** forms and remains stable in polar aprotic solvents (Figure S45). However, **17** was quantitatively converted to **16a** in methanol. Therefore, we attribute this process to a later phase of kinetics (the conversion of the cyan line at 216 s into the blue line at 846 s in Figure 4a). On the other hand, we had no information about the initial intermediate absorbing at ∼310 nm (yellow line at 11 s, Figure 4a). Due to the short lifetime of the intermediate (∼3 min), it could not be isolated or detected by conventional analytical methods such as HPLC or NMR. To gain further insight into its behavior, we repeated this experiment and, immediately after the formation of the intermediate, we added a solution of NaOCH_3_ (50 μL of 0.5 mol L^–1^, cuvette volume ∼2.5 mL) in methanol (*c*_*Z*-**13**_ = 60 μmol L^–1^, *c*_RB_ = 7.7 μmol L^–1^, Figure 4b). Subsequently, the major peak immediately disappeared (Figure 4b) and directly formed propentdyopent products absorbing at ∼277 nm (green-yellow line at ∼19 s in Figure 4b) without the formation of imine **17**. This was expected because compound **17** is also immediately solvolyzed in the presence of a base in methanol. A similar experiment, but with the addition of acid (TFA), gave analogous results (Figure S42). Therefore, the intermediate appears to be unstable in both acidic and basic environments. The target products **16** were found to be stable toward singlet oxygen (Figure S43).

**Figure 5:**
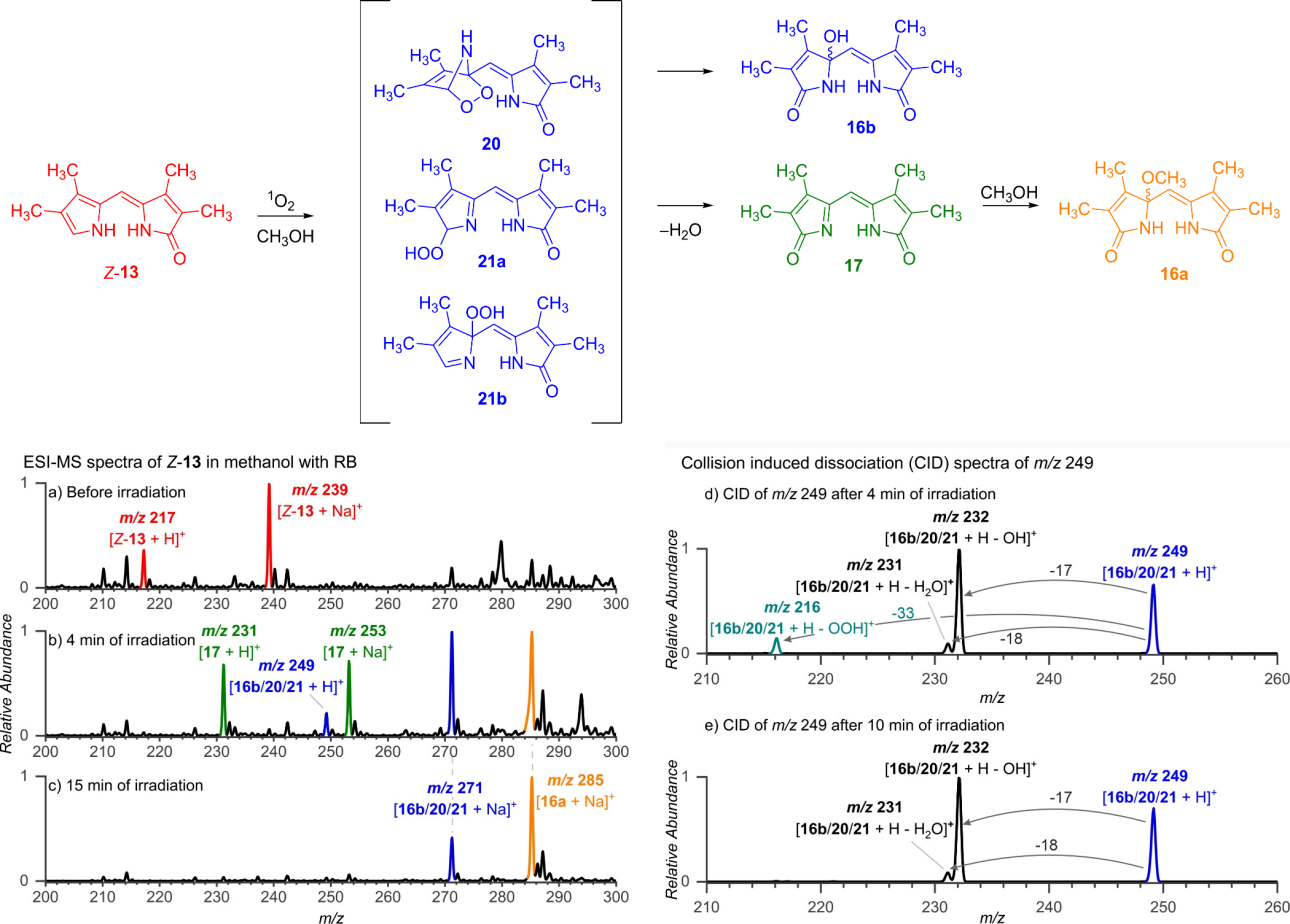
Top: The proposed reaction intermediates and mechanism of RB-sensitized photooxidation of *Z*-**13** in methanol. The structures with the same color share identical *m*/*z* values. Bottom: ESI-MS spectra: (a) before irradiation, (b) after 4 min, and (c) after 15 min of irradiation of the sample at 518 nm (LEDs). Collision-induced dissociation (CID) spectra of ion *m*/*z* 249: (d) after 4 min and (e) after 10 min of irradiation. Color coding of the observed *m*/*z* signals corresponds to the proposed structures.

The MS analysis (*vide infra*) showed that the mass of the intermediate corresponds to that of an adduct of *Z*-**13** with dioxygen. Consequently, we considered endoperoxide **20** and hydroperoxides **21a,b** as possible structures for the target intermediate (Figure 5). These compounds are probably formed upon the interaction of ^1^O_2_ with the pyrrole ring of *Z*-**13**. Among these structures, only **21a** is fully conjugated and would absorb at longer wavelengths (the intermediate absorbs even at ∼400 nm; Figure 4a, orange line at 8 s). In contrast, the conjugation is broken in **20** and **21b**, and these chromophores are expected to absorb similarly as propentdyopents (λ_max_ ∼ 277 nm; Table 1). According to our DFT calculations (B3LYP-D3/6-311+G**, GD3BJ level of theory),^77^**21a** is the most stable isomer compared to **21b** (+75 kJ mol^–1^) and **20** (+200 kJ mol^–1^). Endoperoxides of pyrroles have only been observed at −78 °C in aprotic solvents^57,59^ but they are known to readily undergo methanolysis even at this temperature.^57^ Therefore, endoperoxides must be unstable under the conditions used in our work. Unlike endoperoxides, hydroperoxides can be isolated^78−81^ or were indirectly proven^58^ as final products after photooxidation of some *N*-unsubstituted pyrroles. Based on the stability and absorption properties, we consider structure **21a** to be the most promising short-lived intermediate candidate. Hydroxylated lactams such as **16b** (Figure 5) are commonly observed products during the photooxidation of pyrroles.^52,53,62^ These compounds are usually proposed to be formed by rearrangement from the initial endoperoxides,^53,59,62^ therefore, we also consider endoperoxide **20** in the reaction mechanism.

To gain insight into the reaction mechanism, we performed an ESI-MS experiment similar to that in Figure 4a but with lower concentrations of *Z*-**13** and RB (*c*_*Z*-**13**_ = 27 μmol L^–1^, *c*_RB_ = 3 μmol L^–1^). Before the start of irradiation, *Z*-**13** was detected in the mass spectrum as [**13** + H]^+^ (*m*/*z* 217) and [**13** + Na]^+^ (*m*/*z* 239) ions (Figure 5a, red lines). After 4 min of irradiation with 518 nm (LEDs), new species with *m*/*z* 249 and *m*/*z* 271 appeared, corresponding to protonated **13** and an adduct of [**13** + Na]^+^ with O_2_, respectively. These probably correspond to the proposed intermediates **20** and **21** as well as the final product **16b** (Figure 5b, in blue). Other detected species included imine **17** (in green), represented by the ions [**17** + H]^+^ (*m*/*z* 231) and [**17** + Na]^+^ (*m*/*z* 253), and methanol-propentdyopent **16a** (in orange) as an adduct with sodium, [**16a** + Na]^+^ (*m*/*z* 285). After 15 min of irradiation, only the final products **16a** and **16b** were present in the reaction mixture (Figure 5c). The extracted kinetic profiles from the observed species are shown in Figure S49.

Collision-induced dissociation (CID) experiments provide information about the structure of mass-selected ions.^82^ Fragmentation pattern of the ions with *m/*z 249 (corresponding to [**16b**/**20**/**21** + H]^+^) depended on the irradiation time, suggesting that the ions are a mixture of different isomers. After 4 min of irradiation (Figure 5d), the ions eliminated hydroxy (17 Da) or hydroperoxy (33 Da) radicals or a water molecule (18 Da). After 10 min irradiation, the elimination of the hydroperoxy radical disappeared (Figure 5e). The elimination of the hydroperoxy radical is expected for hydroperoxides **21** (Figure 5). The temporal occurrence of these ions agrees with the intermediate role of **21**. The two other fragmentation channels probably originate from product **16b**, eliminating either HO^•^ or H_2_O (Figures 5d, e). Notably, we did not observe any elimination of O_2_ (32 Da), which would be expected for endoperoxide **20**, in analogy with dissociations of similar endoperoxides tetraanthraporphyrazine^83^ or **DMNO**_**2**_ (Figure S50).

### Ion Spectroscopy

**Figure 6:**
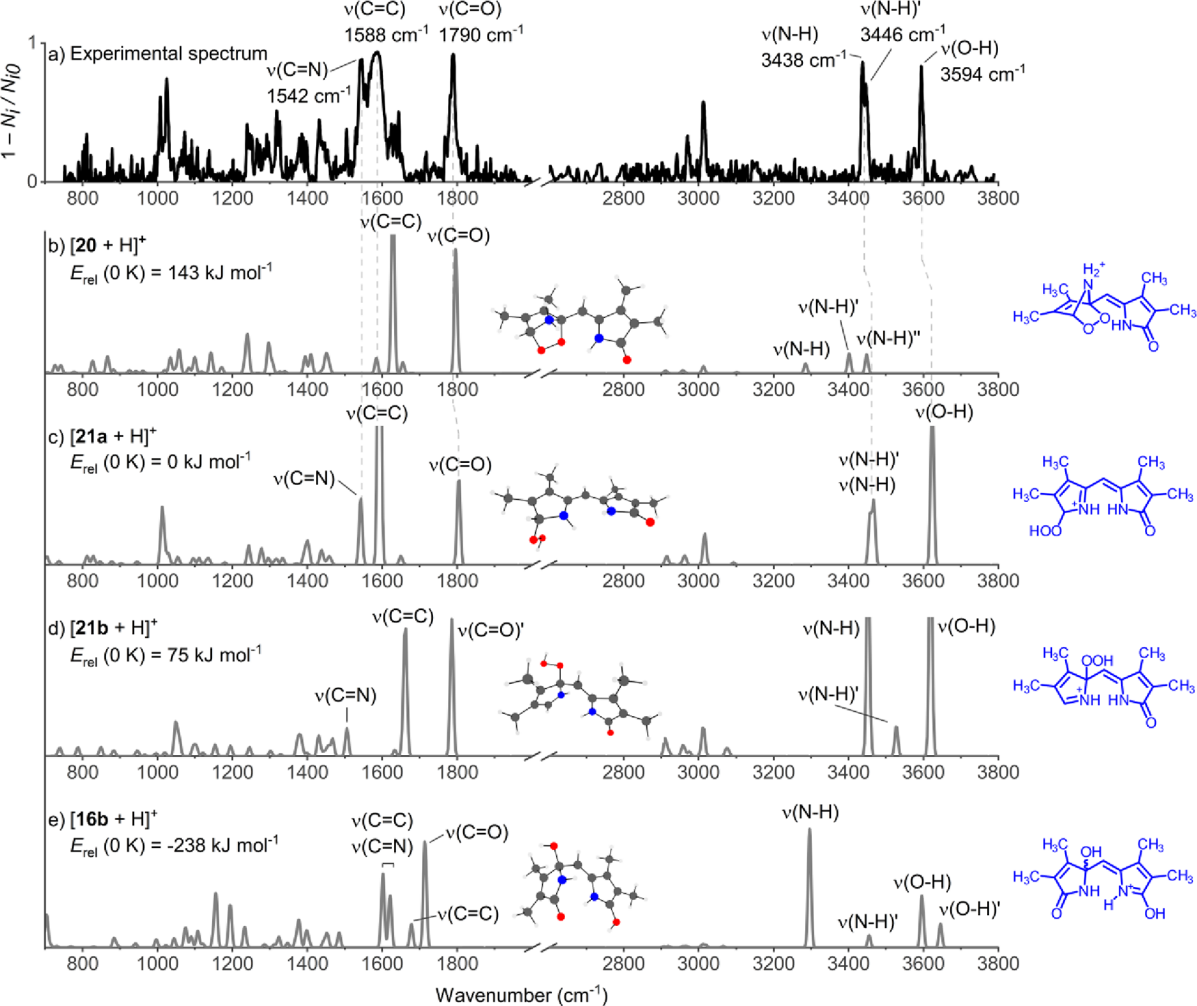
(a) Comparison of the experimental helium tagging IRPD spectrum of an ion with *m*/*z* 249 with the theoretical IR spectra of (b) [**20** + H]^+^, (c) [**21a** + H]^+^, (d) [**21b** + H]^+^, and (e) [**16b** + H]^+^. The theoretical IR spectra were calculated using B3LYP/6-311+G**, GD3BJ and the scaling factor of 0.98 for *v* < 2000 cm^–1^ and 0.96 for *v* > 2000 cm^–1^.

Helium tagging infrared photodissociation (IRPD) spectroscopy^84,85^ was used for the final assignment of the detected ions with *m/*z 249 ([**16b**/**20**/**21** + H]^+^). The experiments were performed with the Infrared Spectroscopy of Reaction Intermediates (ISORI) instrument equipped with an electrospray ionization (ESI) source.^84,85^ A methanol solution containing *Z*-**13** and RB (*c*_*Z*-**13**_ = 27 μmol L^–1^, *c*_RB_ = 3 μmol L^–1^) was infused into the ESI source using a transparent glass capillary while irradiating with 518 nm LED (see Figure S56), corresponding to the reaction time on the order of 10 s or seconds. The monitored ions were mass isolated and trapped in a cryogenic trap operating at 3 K using a helium buffer gas. The trapped ions were thermalized and formed complexes with helium atoms, subsequently used to record IRPD spectra. The obtained experimental spectrum of the ions with *m*/*z* 249 was compared with the predicted IR spectra^77^ of protonated isomers **20**, **21a**, **21b**, and **16b** (Figure 6). The IRPD spectrum matches excellently the theoretical IR spectrum of [**21a** + H]^+^. Namely, we detected an oversaturated peak at 1588 cm^–1^, corresponding to the intense C = C stretching vibration between the pyrrole rings of the [**21a** + H]^+^. In addition, we detected the characteristic carbonyl vibration ν(C = O) at 1790 cm^–1^, the ν(C = N) stretch at 1542 cm^–1^, two ν(N–H) stretches at 3438 and 3446 cm^–1^, and the ν(O–H) stretch at 3594 cm^–1^ (Figure 6a,c), all matching the pattern in the theoretical spectrum of [**21a** + H]^+^. The theoretical spectrum of the [**21b** + H]^+^ isomer (Figure 6d) differs in the C = C stretching vibration, which is slightly blue-shifted (1662 cm^–1^) with respect to the experimental value, and the N–H stretching vibrations that are distinctly more separated than those detected in the experiment. The calculated IR spectra of other ions (**20** and **16b**) differed significantly in most of the detected bands (Figure 6a). For instance, the ion [**16b** + H]^+^ (Figure 6d) was found to have distinct vibrational mode of N–H group at 3296 cm^–1^, while [**20** + H]^+^ (Figure 6b) showed no stretching bands of the O–H group.

### Reaction Kinetics in CD_3_OD

**Figure 7:**
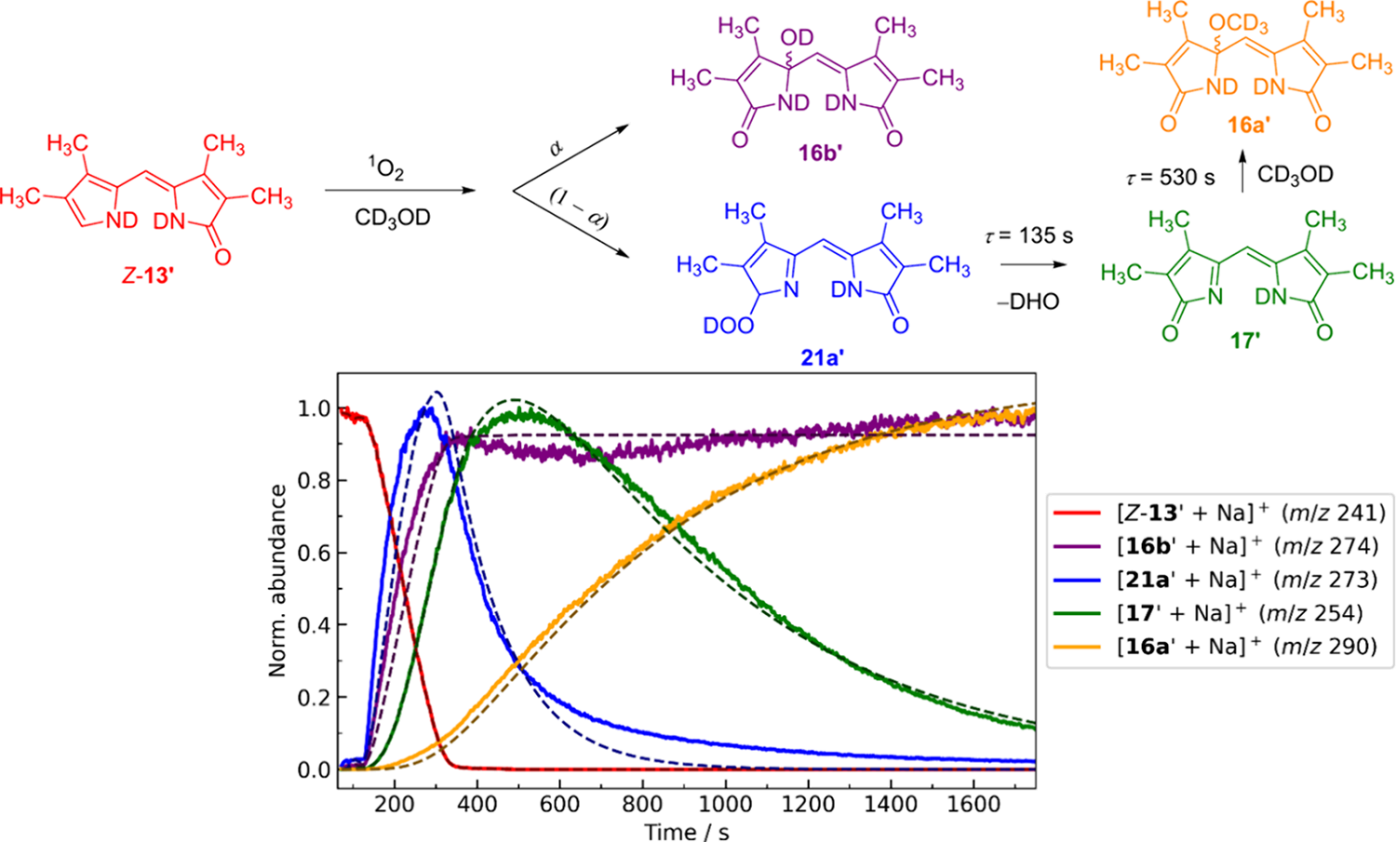
Kinetic traces (solid lines) of the ions formed during the dye-sensitized photooxidation of *Z*-**13** in CD_3_OD. The dashed lines are the fittings to a proposed reaction scheme depicted at the top of the figure. See the extended discussion on the mathematical model on page S64 of Supporting Information.

For further insight into reaction kinetics, we monitored the time evolution of the detected ions. Using CD_3_OD as solvent allowed us to take advantage of the different number of acidic hydrogen atoms in **16b** and **21a**, which, after the H/D exchange, appeared at different *m*/*z* values (Figure 7; with deuterated structures denoted by prime symbols). The ESI-MS spectra at different time points are shown in Figure S51. The extracted kinetic traces of different ions in Figure 7 demonstrate the successful separation of the proposed intermediate **21’** ([**21’** + Na]^+^, *m*/*z* 273, blue line) from water-propentdyopent **16b’** ([**16b’** + Na]^+^, *m*/*z* 274, purple line). The traces of ions *m*/*z* 274 and *m*/*z* 273 clearly show that both ions are produced as initial, observable intermediates from the starting material ([*Z*-**13’** + Na]^+^, *m*/*z* 241, red line). The intensity of *m*/*z* 274 remains practically constant at the late stage of the kinetics (>250 s, purple line, Figure 7), indicating that the major population of *m*/*z* 274 cannot be produced from ion *m*/*z* 273 (blue line). The rise of imine ([**17’** + Na]^+^, *m*/*z* 254, green line) is clearly delayed compared to both ions *m*/*z* 274 and *m*/*z* 273. The fact that the kinetic trace of *m*/*z* 273 decays and *m*/*z* 254 rises suggests that *m*/*z* 254 is produced from *m*/*z* 273 ion (blue line, Figure 7). Finally, as *m*/*z* 254 decays, the signal from methanol-propentdyopent ([**16a’** + Na]^+^ (*m*/*z* 290, orange line) rises. These trends were also observed in methanol (Figure S49). The results of CID experiments for the ions generated from the CD_3_OD solution are discussed in the Supporting Information (see Figures S52 and S53, page S60). In addition, an analogous IRPD experiment was conducted in CD_3_OD for *m*/*z* 252 ion ([**21a’** + D]^+^), supporting our assignment of **21a’** (Figure S55).

The time-resolved data described above were assembled into a reaction scheme (Figure 7 top) and fitted to the experimental traces (the fits are depicted as dashed lines). The choice of branching ratio α (Figure 7 top) is irrelevant because the kinetics traces are normalized (Supporting Information, page S64). The fitting qualitatively agrees with the experimental data. The lifetimes of hydroperoxide **21a’** and **17’** were determined to be 135 and 530 s, respectively. Minor discrepancies may arise due to different ionization of substances in a mixture with a constantly changing composition, or more complex kinetics that cannot be explained by the model we used (see extended discussion on page S66). In addition, an alternative model was tested, which postulates that **16b’** originates from hydroperoxide **21a’** and not directly from *Z*-**13’**. However, in this case, significant deviations were observed between the fitting and the experimental data (Figure S57). The kinetic model (Figure 7) was also applied to fit globally the previously measured kinetics of the photooxidation of *Z*-**13** in methanol using UV–vis spectroscopy (see extended discussion on pages S67–68 and Figure S58, Supporting Information).

From the recorded kinetics, it is clear that both **16b** and **21a** are initial intermediates formed after the initial decay of *Z*-**13**. This is also evidenced by the small peak at ∼277 nm corresponding to **16b** in the initial spectrum during *Z*-**13** photooxidation (orange line, 8s, Figure 4a). This suggests that we should observe signals from both **16b** and **21a** in our IRPD analysis in methanol. However, the signals from **16b** were not observed in the experimental spectrum (Figure 6a, e). We speculate that this absence is due to the significant excess of **21a** compared to **16b**. The population of **21a** is ∼2-fold higher than that of **16b**, as evidenced by the high overall yield of **16a**, which is produced exclusively from **21a** via **17**, and the small overall yield of **16b** (Table 2).

### Reaction Kinetics of *Z*-13 with Thermally Produced Singlet Oxygen in Methanol

**Figure 8:**
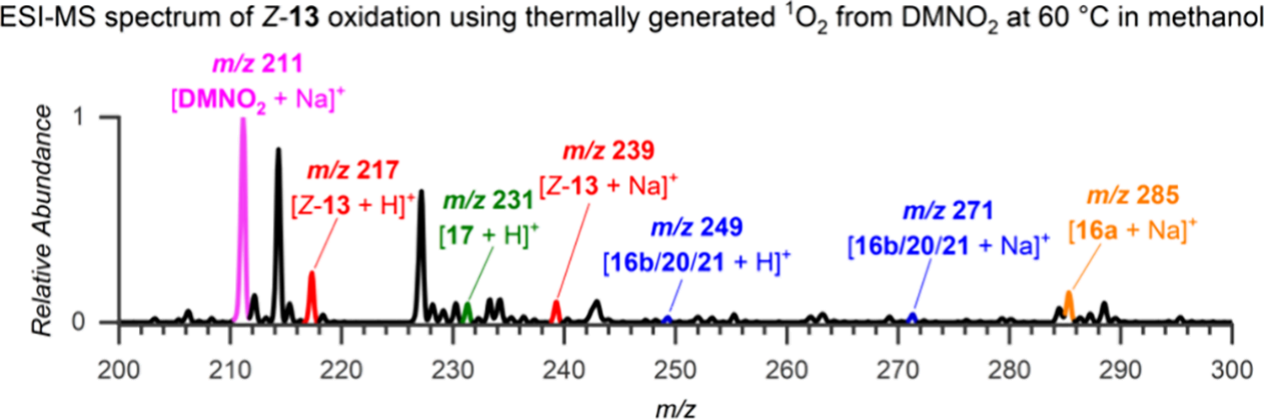
ESI-MS spectrum of *Z*-**13** oxidation using thermally generated ^1^O_2_ from **DMNO**_**2**_ at 60 °C in methanol. See the Experimental Part for details.

Oxidation of *Z*-**13** by thermally produced ^1^O_2_ from **DMNO**_**2**_ was much slower than the sensitized reaction. Therefore, it was necessary to adjust the ratio of *Z*-**13** and **DMNO**_**2**_ for the ESI-MS experiment (Figure 8) to achieve optimal results. When a higher concentration of **DMNO**_**2**_ was used, its strong signal dominated the signals of the *Z***-13** adducts with proton and sodium, as well as the resulting intermediates and products, making their accurate detection and analysis difficult. On the other hand, the oxidation process was too slow at low **DMNO**_**2**_ concentrations, resulting in poor signal intensity.

Although the signals obtained were weaker compared to those from dye-sensitized photooxidation (Figure 5), we were able to detect *m*/*z* 249 (Figure 8) corresponding to the [**21a/16b** + H]^+^ ion and record its fragmentation (Figure S54). These results were consistent with the CID experiments with the ions obtained from the sensitized reaction. The kinetics of *Z*-**13** oxidation by **DMNO**_**2**_ was also followed by UV–vis spectroscopy at 30 °C; however, due to the low rate, the intermediates **21a** and **17** were not detected (Figure S44). Rates of the reaction and quenching of ^1^O_2_ by *Z*-**13** in methanol were determined using **DMNO**_**2**_ (Figure S59, Table S3); the rate constant of 1.0 × 10^9^ M^–1^ s^–1^ was lower than that of quenching (2.3 × 10^9^ M^–1^ s^–1^, see the Supporting Information for details). Such values were also found in the case of other dipyrrinones (*k*_*r*_ + *k*_*q*_ = 0.2–4.2 × 10^9^ M^–1^ s^–1^)^33^ and bilirubin.^86^

The ability to thermally produce singlet oxygen makes it possible to test whether ^1^O_2_ is able to react with *Z*-**13** by an electron transfer reaction to form superoxide, since electron transfer reactions cannot simply be avoided during a dye-sensitized reaction. To test whether free superoxide (pathway *iii* in Scheme 2) is produced to some extent during the process, we used Nitro Blue Tetrazolium (NBT), which is selectively reduced by O_2_^•–^ to monoformazan, detectable at ∼530 nm.^87^ However, the results from our experiments were ambiguous, and we could not draw any conclusions from them (see discussion on page S54 of Supporting Information).

### Proposed Mechanism and Discussion

**Scheme 5:**
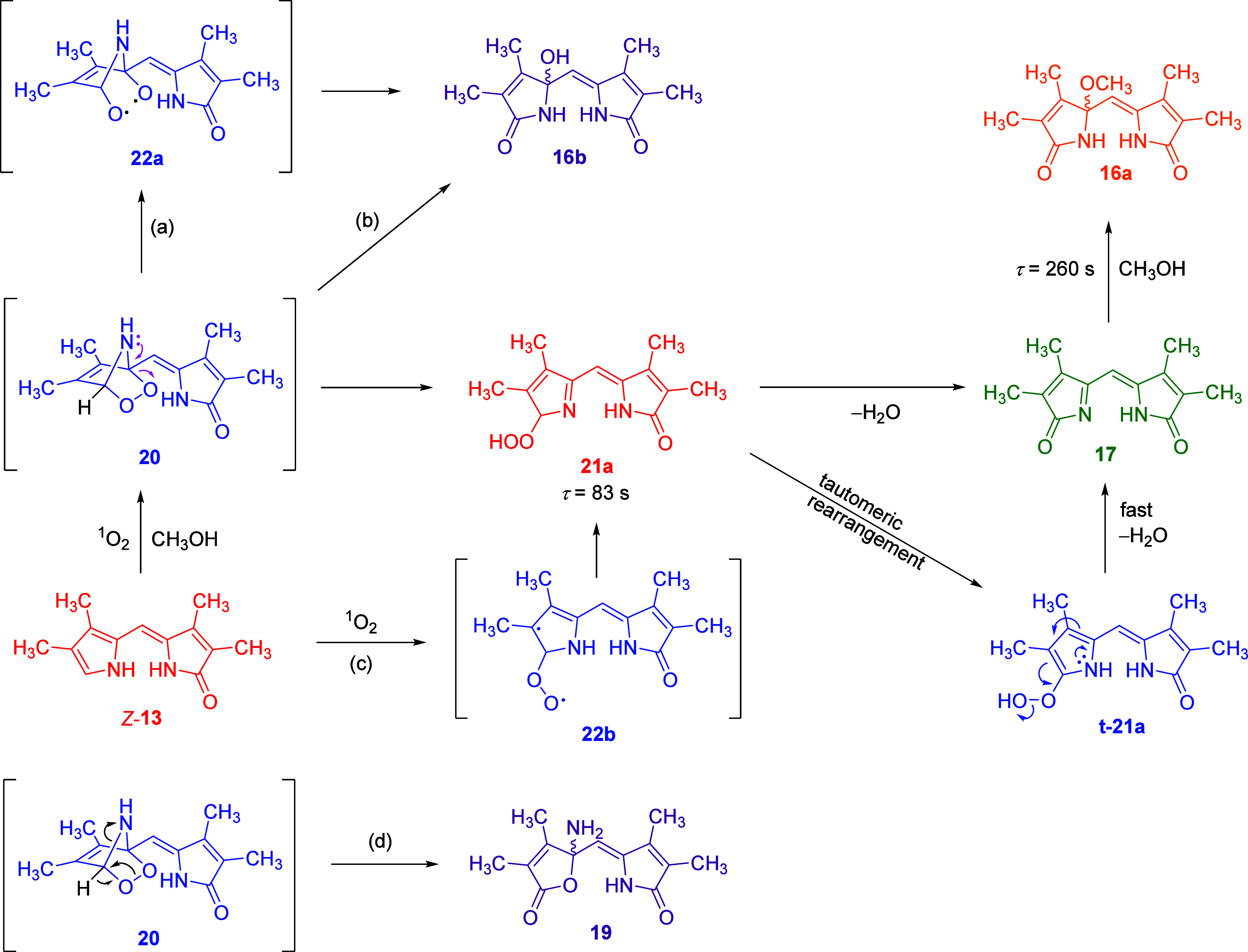
Proposed Mechanism of the Major Reaction of *Z*-**13** with ^1^O_2_ in Methanol

From the IRPD results, it is evident that the short-lived intermediate is hydroperoxide **21a**. We have never directly observed the proposed endoperoxide **20**. The only indirect evidence for its formation from our data is the kinetic observation that **16b** is obtained from *Z*-**13** (Figure 7). It is highly unlikely that **16b** would form in a single transformation between ^1^O_2_ and *Z*-**13**. Therefore, a short-lived endoperoxide **20**, whose rate of disappearance is higher than its formation rate, would explain such an observation. It is likely that **16b** is formed by the rearrangement of **20**, probably via biradical intermediate **22a** (path a, Scheme 5), or by base- or solvent-assisted rearrangement of endoperoxide **20** (path b, Scheme 5), as suggested previously.^53,59,62^ The observed hydroperoxide **21a** can be produced directly by the ene-reaction of ^1^O_2_ and *Z*-**13** (path c, Scheme 5) or by the ring opening of endoperoxide **20** (Scheme 5). From our data, we cannot conclusively determine which pathway takes place. However, as mentioned in the previous discussion, the ene-reaction is a two-step process involving biradical intermediate **22b** (Scheme 5) with higher activation energy than the [4 + 2] addition to yield endoperoxide.^54^ Therefore, we propose that **21a** is formed by the rearrangement of endoperoxide **20** (Scheme 5).

Subsequently, compound **21a** undergoes dehydration to produce imine **17** (Scheme 5), as found in our kinetic experiments. The reaction is accelerated by the presence of base and acid (Figures 4b and S42). We hypothesized that the reaction could occur directly or via tautomer **t-21a** (Scheme 5), which would be prone to dehydration due to the electron-donating effect of pyrrole moiety. With typical substrates (aliphatic hydroperoxides), direct dehydration of hydroperoxides to ketones does not occur.^88^ The closest equivalent reaction is the Kornblum–DeLaMare rearrangement,^89^ which produces ketones from diaryl/dialkyl peroxides under basic or acidic catalysis. However, this rearrangement does not proceed for hydroperoxides. Therefore, hydroperoxides should be stable under the conditions studied. It is also possible that the tautomerization of **21a** is the rate-determining step. Unfortunately, tautomerization rates of the conversion of 2*H*-pyrrole to 1*H*-pyrrole have not been reported. Furthermore, pyrrole **t-21a** would have significant absorption in the visible region and should be clearly observed in the UV–vis data. We did not observe such signals; therefore, we propose that the most plausible mechanism is a solvent-assisted one-step process from **21a** to **17**.

Amine **19** was observed as a minor product during photooxidation reactions in methanol (∼10%) and acetonitrile (∼20%, Table 2). This yield increased significantly at −78 °C in methanol (entry 6, Table 2), suggesting that a homolytic pathway of endoperoxide decomposition via biradical **22** (Scheme 5) leading to **19** is unlikely. We propose that a solvent-assisted rearrangement of endoperoxide **20** (path d, Scheme 5) could lead to product **19**. As discussed earlier, given that eT reaction affording superoxide and radical cation of pyrrole (Scheme 2) may be feasible and can occur to some extent, product **19** and/or some of the unidentified reaction products could also be formed by eT mechanism.

**Scheme 6:**
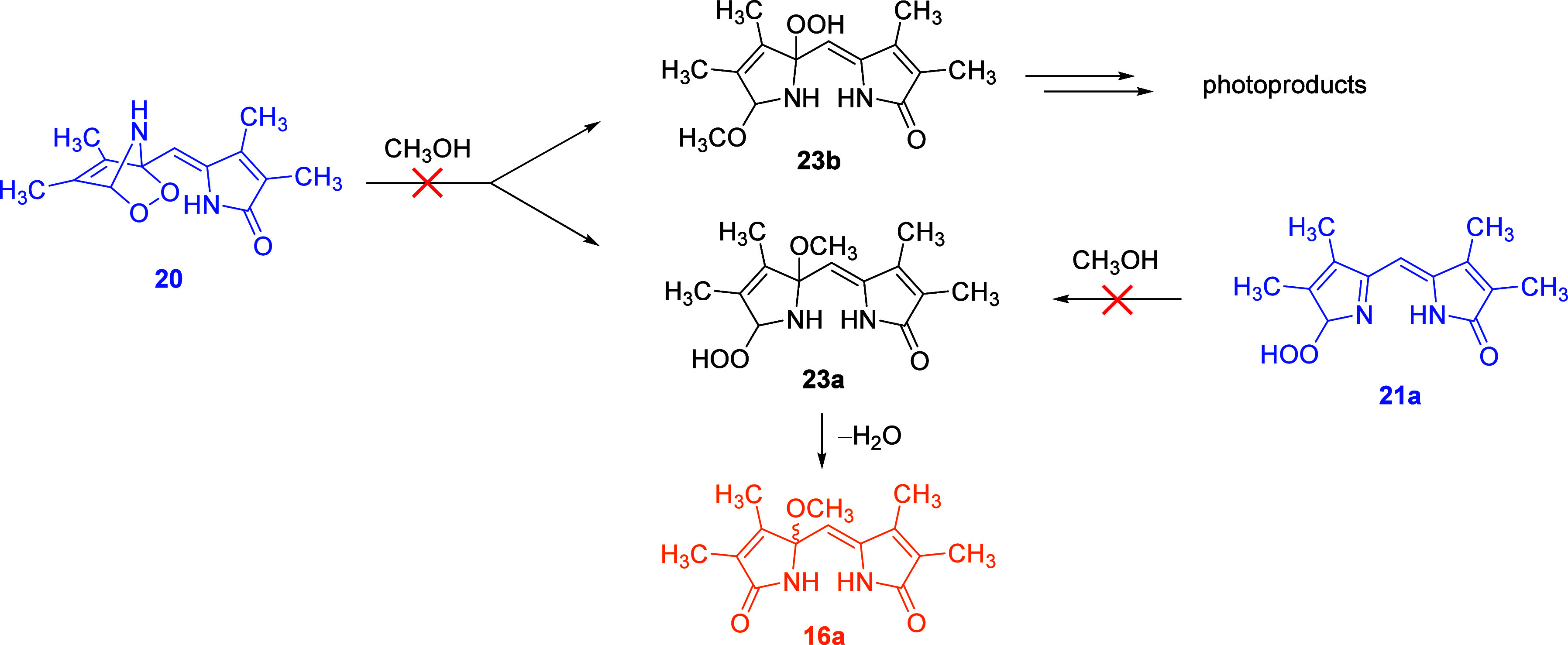
Addition of Methanol to Endoperoxide **20** and Hydroperoxide **21a** That Does Not Occur

In the earlier literature,^35,53,59,62,90^ it was suggested that pyrrole endoperoxides such as **20** can also undergo solvolysis (here, methanolysis) to hydroperoxides such as **23a** and **b** (Scheme 6). Similarly, nucleophilic attack of methanol on **21a** could afford **23a** (Scheme 6). **23a** could then directly produce **16a** (Scheme 6), similar to how **21a** can provide **17** (Figure 7). We never observed ions corresponding to compounds **23a** and **b**; moreover, as evident from our kinetic experiments (Figure 7), **17** is the only precursor for **16a**. Therefore, we rule out the pathways depicted in Scheme 6, although this conclusion may not hold for the photooxidation of other pyrrole derivatives.

As described in the Introduction, 2-methyl substituted dipyrrinones do not give propentdyopents as major products. Instead, the monopyrrolic fragments **3** and **4** (Scheme 1), thought to originate from a dioxetane intermediate, are observed as the major products. We suggest that upon the reaction of ^1^O_2_ with 2-methyl-substituted dipyrrinone such as xanthobilirubic acid, the major intermediate could be a hydroperoxide analogous to that of **21a**; however, a hydroperoxide bearing the 2-methyl group should be stable as it cannot simply undergo solvent-assisted dehydration. The photooxidation of xanthobilirubic acid was autocatalytic and possibly involved hydroperoxide as the catalytic species.^30^ Based on our results, the hydroperoxide analogous to **21a** would be the catalyst that would dissociate into radicals upon irradiation and catalyze the radical pathway for the production of **3** and **4-**like products as suggested by Strope and co-workers^30^ and supported by other evidence.^21,34^

## Conclusions

In this work, we investigated the ^1^O_2_ oxidation of the model dipyrrinone *Z*-**13** using UV–vis spectroscopy and ESI-MS kinetics. Despite the fact that oxidation of pyrrole with singlet oxygen usually leads to complex mixtures of products, in methanol, we identified propentdyopents **16a** and **16b** as the main products, along with hydroperoxide **21a**, confirmed by the IRPD experiments, and imine **17**, which are significant but previously unreported intermediates. Furthermore, we successfully fitted the kinetic model of the proposed mechanism to our ESI-MS kinetic data. We found that both **21a** and **16b** are probably produced from endoperoxide **20**, which we proposed as a common intermediate directly formed by reaction of *Z*-**13** with ^1^O_2_. We did not observe products formed by the addition of methanol to endoperoxide **20** and hydroperoxide **21a** (Scheme 6) as proposed earlier. Irradiation of *Z*-**13** in aprotic nonpolar solvents such as toluene led to the formation of covalent dimers due to the self-association of *Z*-**13**.

The implications for the oxidation of bilirubin or other bilirubin models, especially those containing a 2-methyl group, are striking. We believe that the first step of the mechanism (formation of hydroperoxide) should occur also during the oxidation of these substrates. Another open question is also the mechanism of oxidation upon direct irradiation, where other oxidation mechanisms besides the singlet oxygen reaction may occur. These issues need to be addressed in further studies.

## Experimental Section

### Materials and Methods

NMR spectra were obtained on 126 MHz (^13^C) and 300 and 500 MHz (^1^H) spectrometers in chloroform-*d*, dichloromethane-*d*_2_, dimethylsulfoxide-*d*_6_, CF_3_CO_2_D, and they were referenced^91^ to the residual peak of the solvent. All NMR measurements were conducted at 25 °C, unless specified. IR spectra were recorded on a Fourier transform spectrometer using solid samples. HRMS spectra were obtained in positive modes on a triple quadrupole ESI/APCI mass spectrometer. Mass spectrometric experiments were recorded using a mass spectrometer equipped with an ESI source. IRPD spectra were collected with the ISORI instrument.^84,85^ HPLC (C-18 column) analyses were performed by gradient elution using water and methanol. UV–vis spectra were measured in 10.0 mm quartz fluorescence cuvettes on a UV–vis spectrometer. Emission spectra were recorded on a luminescence spectrometer in 10.0 mm quartz fluorescence cuvettes or in special rounded cuvettes places in a cryostat when measured at 77 K. Column chromatography procedures were performed using columns packed with silica gel (63–200 μm) or on reverse phase (C18) columns. Thin-layer chromatography (TLC) was performed using silica gel plates (0.2 mm thickness) and visualized under a UV lamp (254 nm). All solvents and chemicals were used as purchased or purified/dried by standard procedures when necessary. Unless otherwise specified, all procedures were carried out under a nitrogen atmosphere. The manipulation with photosensitive compounds was performed under dim light to prevent photoisomerization. Reactions requiring heating were heated using position heating blocks. Structural assignments were made with additional information from gCOSY, gHSQC, and gHMBC experiments.

### Fluorescence Quantum Yields

Fluorescence quantum yields (Φ_*F*_) of propentdyopents **16a** and **16b** in methanol were determined as the absolute values using an integration sphere with the excitation wavelength of 266 nm (Δλ = 12 nm). The quantum yields were measured three times and then averaged for each sample. The concentrations of the solutions were kept low (*A* < 0.1).

### Photoisomerization Quantum Yields

A freshly prepared methanolic solution of a dipyrrinone isomer (*Z*-**13** or *E*-**13**; ∼ 50 μmol L^–1^, 3.0 mL) in a fluorescence cuvette equipped with a stir bar was inserted into a UV–vis spectrometer equipped with a 3D printed holder consisting of a LED source, camera lense, cooling fan, and calibrated photodiode.^36^ This solution was irradiated with LEDs, and time-dependent absorption spectra were recorded at room temperature for the given time under stirring. The incident photon flux was determined by a photodiode head using a pure solvent in the cuvette. The measurements performed at the same irradiation wavelength were repeated 4 times for each isomer. The set of experiments was performed for two different irradiation sources (400 and 420 nm; see spectra in Figure S64).

### UV–Vis Experiments

#### Irradiation Experiments

A stock solution of dipyrrinones (*Z*-**13** or *E*-**13**) or propentdyopents **16a** and **16b** (30–80 μmol L^–1^) was prepared in a given solvent (methanol, acetonitrile, or toluene). 2.0–3.0 mL of solution was added to the fluorescence cuvette equipped with a cap and stir bar. A cuvette with the sample was irradiated with LEDs (Figure S64). In the case of sensitization experiments, a stock solution of sensitizer (RB or MB; ∼ 180 μmol L^–1^) was prepared in the same solvent, and a small aliquot (30–100 μL) was added to the cuvette. When required, the cuvette was purged with O_2_ for 5 min. For degassed solutions, the stock solution was pipetted into a Schlenk flask fused with a fluorescence cuvette and degassed by three freeze–pump–thaw cycles.

#### Kinetics in the Dark

A solution of imine **17** in dichloromethane (*c* ∼ 0.5 mmol L^–1^) was prepared, and a small aliquot (100 uL) was quickly added to the cuvette containing methanol (2.5 mL) and equipped with a stir bar, placed in the absorption spectrometer. After the addition, the solution was quickly mixed with a spatula, and the absorption spectra were recorded under stirring.

A fresh stock solution of **DMNO**_**2**_ (*c* = 10.5 mmol L^–1^), *Z*-**13** (*c* ∼ 60 μmol L^–1^), and NBT (*c* = 0.72 mmol L^–1^) in methanol were prepared. Methanol (2.5 mL) or solution of *Z*-**13** was added to the cuvette equipped with a stir bar, followed by an endoperoxide stock solution (100 μL) and an NBT solution (20 μL). The cuvette was placed in a temperature-controlled cuvette holder preheated to 30 °C, and the kinetics was recorded for at least 2 h while maintaining 30 °C under stirring.

#### Reaction and Quenching Rate Constants of **13** with Singlet Oxygen

A stock solution of *Z*-**13** or *E*-**13** (30–80 μmol L^–1^) and **DMNO**_**2**_ (*c* ∼ 11 mmol L^–1^) was prepared in a given solvent (methanol or acetonitrile). A solution of **13** (2.5 mL) was added to the cuvette equipped with a stir bar, followed by an endoperoxide stock solution (200 or 400 μL). The cuvette was placed in temperature-controlled cuvette holder preheated to 25 °C, and the kinetics was recorded for 1 h while maintaining 25 °C under stirring.

### HPLC Irradiation Experiments

Similar to the irradiation experiments described above, a solution of dipyrrinones (*Z*-**13** or *E*-**13**) with a higher concentration (∼0.7 mmol L^–1^) was prepared. 2.0 mL of the stock solution was added to the HPLC transparent vial, followed by the addition of a fresh solution with a sensitizer (30–100 μL) prepared in advance so that the final concentration of the sensitizer was ∼4 μmol L^–1^ (<1 mol % of the dipyrrinone concentration). When required, additional additives such as CF_3_COOH (Table 2, entry 5; 20 μL) or NaOCH_3_ (Table 2, entry 7; 50 μL of 5 mol L^–1^; methanolic solution) were added. The vial was then purged with oxygen while simultaneously irradiated with a LED source (532 nm for RB and 655 nm for MB sensitizers) for 5 min. For entries 10a, b (Table 2), 1.0 mL of the photolysate was pippeted to another HPLC vial. Then, water or methanol (0.5 mL) was added to each vial, which was kept for 2 h at room temperature in the dark. Subsequently, HPLC data were recorded. Table 2 shows only the relative yields calculated from the corresponding areas integrated at 277 nm for propentdyopent-like compounds (**16a**, **16b**, **19**, and unidentified compounds **S4ab**; Supporting Information), 353 nm for unidentified compounds **S5a**,**b**, and 222 nm for **18** (Supporting Information). For some direct irradiation experiments, the vial with the sample was purged for 5 min with O_2_ and then sealed. The vials were irradiated with LEDs in a photoreactor (Figure S62).

### MS Kinetic Experiments

The setup used for the kinetic measurements consisted of a sample introduced through a silica capillary to the ionization source of the mass spectrometer instrument. To study the reactivity of *Z*-**13** with photochemically produced ^1^O_2_, a sample was prepared by dissolving *Z*-**13** and rose bengal in methanol or methanol-*d*_4_ (*c*_*Z*-**13**_ = 27 μmol L^–1^, *c*_RB_ = 3 μmol L^–1^). The diluted sample was placed to a transparent vial, which was continuously irradiated with 540 nm LEDs, while nitrogen overpressure drove the sample to the mass spectrometer for the monitoring of the reaction with ESI(+)-MS (Figure S56a).

The reaction between *Z*-**13** and thermally produced ^1^O_2_ was carried out with the help of the automated setup, which regulates a constant flow to ESI-MS. Two syringes were filled with solution of *Z*-**13** and **DMNO**_**2**_ dissolved in methanol (*c*_*Z*-**13**_ = 110 μmol L^–1^, *c***_DMNO2_** = 550 μmol L^–1^), while the third syringe contained only methanol. These solutions were delivered with the help of a syringe pump into a T-piece, where they mixed in a controlled ratio. The optimal total flow rate for the reaction was determined to be 8 μL min^–1^, allowing sufficient time for the reaction to proceed. This flow was adjusted to 1.8 μL min^–1^ for the *Z*-**13** solution, 1.8 μL min^–1^ for the **DMNO**_**2**_ solution, and 4.4 μL min^–1^ for methanol. The resulting reaction mixture was then transported through a heating element set to 60 °C before entering the mass spectrometer.

The conditions for mass spectrometric measurements using a linear ion trap LTQ were as follows: 5 kV electrospray voltage, 220 °C capillary temperature, 5 V capillary voltage, 40 V tube lens voltage, 5 au sheath gas flow, 1 au auxiliary gas flow, and 1 au sweep gas flow. The CID experiments were carried out using the same instrument and with identical parameters. The collision energy for fragmentation was between 15 and 22%. The data were analyzed using a commercial software.

### IRPD Studies

Infrared photodissociation (IRPD) experiments were performed using an Infrared Spectroscopy of Reaction Intermediates (ISORI) instrument equipped with an electrospray ionization (ESI) source.^84,85^ The ions of interest are mass-selected by a quadrupole mass filter and transferred to a cryogenic ion trap operated at 3 K. The thermalized ions attached helium atoms. These helium-tagged ions were used for spectroscopic measurements. The ions were irradiated in alternative cycles using OPO/OPA pumped by Nd:YAG laser (tuning range 600–4700 cm^–1^, fwhm ∼1.5 cm^–1^, 10 ns pulse length). After irradiation, the ions were extracted, mass analyzed by a quadrupole, and detected by a Daly type detector. The IRPD spectra are constructed as 1–*N*(ν_*i*_)/*N*_0_, where *N*(ν_*i*_) is the number of helium complexes after the irradiation, and *N*_0_ is the number of helium complexes in alternative cycles without the irradiation.

### Data Analyses

Two-dimensional HPLC data were corrected for baseline drift^92^ (caused mainly by a gradient elution method) using an asymmetrically reweighted penalized least-squares (arPLS)^93^ method modified for two-dimensional data (based on robust smoothing using a discrete cosine transform).^94^ Isomerization quantum yields were fitted using an HS-MCR method according to our previous implementation.^36^ Other fitting routines were implemented in Python (see Supporting Information for details about the kinetic models). Nonlinear least-squares minimization was implemented using a Python’s package LMFIT.^95,96^

#### Synthesis

##### 3,4-Dimethylpyrrol

The compound was prepared according to a known procedure^67^ with slight modifications. 500 mL three-necked round-bottomed flask, equipped with a magnetic stirrer and a condenser, was dried and filled with dry nitrogen. Ethyl carbamate (24.3 g, 273 mmol) was added, followed by dry toluene (180 mL). The mixture was cooled in an ice bath to 0 °C. Then, freshly distilled SOCl_2_ (20 mL, 273 mmol) and dry pyridine (2.0 eq, 44 mL, 546 mmol) were added dropwise. The mixture was stirred at 0 °C for 10 min, then warmed to room temperature, and stirred for an additional 1 h. Then, 2,3-dimethyl-1,3-butadiene (31 mL, 273 mmol) was added, and the mixture was heated to 85 °C for 1 h and stirred overnight at room temperature. A white pyridinium hydrochloride precipitate was filtered, and the filter cake was washed with toluene (2 × 50 mL). The filtrates were combined, and the solvent was evaporated under reduced pressure. The crude intermediate (oily residue) was dissolved in a solution of KOH (128 g, 2.28 mol) in methanol (330 mL). The mixture was refluxed under a nitrogen atmosphere for 2.5 h. Methanol was removed by distillation at atmospheric pressure, then an excess of water was added (200 mL), and the mixture was steam-distilled. The distillate was extracted by diethyl ether (3 × 100 mL). Extracts were combined and dried with anhydrous K_2_CO_3_, the solid was filtered, and the solvent was evaporated under reduced pressure. The crude solid was reprecipitated by evaporation from the mixture of hexane and dichloromethane. Yield: 8.93 g (34.3%); pale brown crystalline solid. ^1^H NMR (300 MHz, CDCl_3_): δ (ppm) 7.79 (bs, 1H), 6.53 (s, 2H), 2.06 (s, 6H). The spectroscopic data are consistent with those reported in the literature.^66,97,98^

##### 3,4-Dimethyl-1*H*-pyrrole-2-carbaldehyde (**14**)

The compound was prepared according to a known procedure.^66^ POCl_3_ (1.1 eq, 3.6 mL, 38.5 mmol) was charged into a flask equipped with a magnetic stirrer under a nitrogen atmosphere, and the flask was cooled down with an ice-salt bath. Then, a solution of 3,4-dimethyl-pyrrole (1.0 eq, 3.33 g, 35.0 mmol) and dry dimethylformamide (1.3 eq, 3.5 mL, 45.5 mmol) in dry dichloromethane (50 mL) was added dropwise. The reaction mixture was stirred in an ice-cold bath for 2 h, then allowed to warm to room temperature, and stirred overnight. The solvent was evaporated under reduced pressure, the residue was suspended in water (30 mL), and the mixture was cooled to 0 °C. NaOH (8.60 g, 215 mmol) was slowly added under stirring under a nitrogen atmosphere. Then, the mixture was allowed to warm to room temperature and stirred for 1 h. The yellow precipitate was filtered, and the solid was washed with water (3 × 50 mL). The residue was dissolved in a sufficient amount of dichloromethane (50 mL), and the solution was dried with anhydrous Na_2_SO_4_. The solids were filtered, and the solvent evaporated under reduced pressure. The residue was purified by a short silica-gel column chromatography using ethyl acetate as a mobile phase. Yield: 3.27 g (75.9%); pale-yellow solid. ^1^H NMR (300 MHz, CDCl_3_): δ (ppm) 9.52 (s, 1H), 9.31 (bs, 2H), 6.87 (d, *J* = 3.0 Hz, 1H), 2.28 (s, 3H), 2.02 (s, 3H). Spectroscopic data are consistent with those reported in the literature.^66,99^

##### 3,4-Dimethyl-1,5-dihydro-2*H*-pyrrol-2-one (**15**)

The compound was prepared according to a known procedure.^66^ 3,4-Dimethyl-pyrrole (5.70 g, 59.9 mmol) was dissolved in pyridine (10 mL). Then, hydrogen peroxide (1.9 eq, 30.0%, 12 mL, 113 mmol) was added in one portion, and the mixture was stirred at 50 °C for 2 h. The solvent was removed under reduced pressure, and the crude product was purified by silica-gel column chromatography using gradient elution (0 → 2% methanol in EtOAc). Yield: 4.03 g (60.5%); pale yellow solid. ^1^H NMR (300 MHz, CDCl_3_): δ (ppm) 6.89 (bs, 1H), 3.80 (s, 2H), 1.97 (s, 3H), 1.79 (s, 3H). Spectroscopic data are consistent with those reported in the literature.^66,97^

##### (*Z*)-5-((3,4-Dimethyl-1*H*-pyrrol-2-yl)methylene)-3,4-dimethyl-1,5-dihydro-2*H*-pyrrol-2-one (*Z*-**13**)

The compound was prepared according to a known procedure.^65,66^**14** (1.0 eq, 0.220 g, 1.79 mmol), **15** (1.2 eq, 0.238 g, 2.14 mmol), and KOH (1.5 g) were charged into a flask. The mixture was cooled down to 0 °C in an ice bath, and distilled water (7 mL) was added, followed by methanol (5 mL). The mixture was heated to 65 °C under a nitrogen atmosphere overnight. The mixture was cooled down to room temperature, and distilled water (10 mL) was added. Precipitate was filtered and washed with distilled water (3 × 10 mL) and methanol (2 × 10 mL). The title compound was dried under reduced pressure. Yield: 0.190 g (49.2%); yellow powder. Mp: 250–256 °C decomp. (lit. 254–256 °C, decomp.).^65^^1^H NMR (500 MHz, DMSO-*d*_6_): δ (ppm) 10.43 (s, 1H), 9.66 (s, 1H), 6.73 (d, *J* = 2.2 Hz, 1H), 5.95 (s, 1H), 2.06 (d, *J* = 0.8 Hz, 3H), 2.02 (s, 3H), 1.94 (s, 3H), 1.77 (s, 3H). ^13^C{^1^H} NMR (126 MHz, DMSO-*d*_6_): δ (ppm) 171.9, 141.5, 129.9, 124.1, 123.7, 121.7, 119.8, 118.1, 97.8, 10.0, 9.5, 9.0, 8.3. FTIR (neat, cm^–1^): 3351, 3160, 2912, 1654, 1639, 1086. HRMS (APCI^+^) *m*/*z*: [M + H]^+^ calcd for C_13_H_17_N_2_O^+^ 217.1335; found 217.1337. UV–vis (CH_3_OH): λ_max_(ε) = 396 (32 300) nm (L mol^–1^ cm^–1^). Fluorescence (CH_3_OH): λ_max_ = 454 nm, Φ_*F*_ < 0.01. Spectroscopic data are consistent with those reported in the literature.^66,100^

##### (*E*)-5-((3,4-Dimethyl-1*H*-pyrrol-2-yl)methylene)-3,4-dimethyl-1,5-dihydro-2*H*-pyrrol-2-one (*E*-**13**)

*Z*-**13** (55.0 mg, 254 μmol) was dissolved in methanol (80 mL), and triethylamine (1.0 mL) was added. The solution was irradiated at 400 nm (LEDs; see the emission spectrum in Figure S64) under stirring and under a nitrogen atmosphere for 90 min. The reaction progress was monitored by HPLC. The reaction mixture was evaporated under reduced pressure, and the residue was purified twice by reverse-phase flash chromatography with gradient elution (50 → 100% methanol in 0.05% NH_3_ in water). Yield: 27 mg (49.1%); bright yellow powder. The purity of 95.4% was determined by HPLC at 419 nm (at which both *Z* and *E* isomers possess the same molar absorption coefficient). Mp: 235–240 °C decomp. ^1^H NMR (500 MHz, DMSO-*d*_6_): δ (ppm) 10.17 (s, 1H), 9.66 (s, 1H), 6.55 (s, 1H), 6.16 (s, 1H), 1.93 (s, 3H), 1.87 (s, 3H), 1.84 (s, 3H), 1.75 (s, 3H). ^13^C{^1^H} NMR (126 MHz, DMSO-*d*_6_): δ (ppm) 170.0, 138.2, 137.1, 130.0, 122.5, 118.2, 117.5, 117.0, 102.8, 11.8, 10.2, 9.5, 8.4. FTIR (neat, cm^–1^): 3259, 3137, 2913, 1652, 1614. HRMS (APCI^+^) *m*/*z*: [M + H]^+^ calcd for C_13_H_17_N_2_O^+^ 217.1335, found 217.1338. UV–vis (CH_3_OH): λ_max_(ε) = 400 (23 000) nm (L mol^–1^ cm^–1^).

##### (*Z*)-5-((3,4-Dimethyl-5-oxo-1,5-dihydro-2*H*-pyrrol-2-ylidene)methyl)-5-methoxy-3,4-dimethyl-1,5-dihydro-2*H*-pyrrol-2-one (**16a**)

*Z*-**13** (357 mg, 1.65 mmol) was suspended in methanol (220 mL), and rose bengal (∼2 mg) was added. The mixture was sonicated for 10 min to make a homogeneous suspension. Then, NaOCH_3_ (a 0.5 M solution in methanol, 600 μL, 300.0 μmol) was added. The mixture was then irradiated with 532 nm LEDs (Figure S64) while purging with oxygen for 110 min and under vigorous stirring. The reaction progress was monitored by TLC (5% methanol in dichloromethane, v/v). The suspension was gradually dissolved to form a clear red solution. The solution was quenched by adding a few chips of dry ice. The solvent was evaporated under reduced pressure, and the residue was purified by silica-gel column chromatography using gradient elution (4 → 8% methanol in dichloromethane, v/v). Yield: 420 mg (97%); off-white powder. Mp: 186–188 °C, decomp. (lit. 198–204 °C decomp.).^35^^1^H NMR (500 MHz, CD_2_Cl_2_): δ (ppm) 8.14 (s, 1H), 5.86 (s, 1H), 4.72 (s, 1H), 3.14 (s, 3H), 1.93 (d, *J* = 0.8 Hz, 3H), 1.83 (s, 3H), 1.80 (d, *J* = 0.8 Hz, 3H), 1.77 (d, *J* = 0.8 Hz, 3H). ^13^C{^1^H} NMR (126 MHz, CD_2_Cl_2_): δ (ppm) 172.9, 172.0, 151.2, 142.1, 141.2, 131.4, 129.4, 104.2, 93.0, 50.0, 10.5, 10.1, 8.7, 8.6. FTIR (neat, cm^–1^): 3352, 3220, 3085, 2918, 1693, 1655. HRMS (APCI^+^) *m*/*z*: [M + H]^+^ calcd for C_14_H_19_N_2_O_3_^+^ 263.1390, not found. The molecular peak was not observed, however, iminium **17H**^+^ ([**17** + H]^+^, *m*/*z* 231.1130), a proton adduct of its dimer ([**17** + **17** + H]^+^, *m*/*z* 461.2178) and a proton adduct of **16a** and **17** [**16a** + **17** + H]^+^, *m*/*z* 493.2446) were detected (see Figure S60). UV–vis (CH_3_OH): λ_max_(ε) = 277 (23 200) nm (L mol^–1^ cm^–1^). Fluorescence (CH_3_OH): λ_max_ = 421 nm, Φ_*F*_ < 0.01. Spectroscopic data are consistent with those in the literature.^35,72^

##### (*Z*)-5-((3,4-Dimethyl-5-oxo-1,5-dihydro-2*H*-pyrrol-2-ylidene)methyl)-5-hydroxy-3,4-dimethyl-1,5-dihydro-2*H*-pyrrol-2-one (**16b**)

Propentdyopent **16a** (50.1 mg, 191 μmol) was dissolved in trifluoroacetic acid (5.0 mL). Then, ice-cold distilled water (50 mL) was added in one portion under vigorous stirring. The mixture was then neutralized with saturated aq NaHCO_3_ to neutral pH (pH paper), and the solution was extracted with dichloromethane (5 × 25 mL). The extracts were combined, and the solvent eas evaporated under reduced pressure to get the title products, which was dried under reduced pressure Yield: 35 mg (74%); off-white solid. Mp: 205–208 °C, decomp. ^1^H NMR (500 MHz, DMSO-*d*_6_): δ (ppm) 8.67 (s, 1H), 8.54 (s, 1H), 6.57 (d, *J* = 1.2 Hz, 1H, – O*H*), 4.84 (d, *J* = 1.1 Hz, 1H, ≥ C*H*), 1.93 (d, *J* = 1.0 Hz, 3H), 1.76 (s, 6H), 1.65 (d, *J* = 1.1 Hz, 3H). ^13^C{^1^H} NMR (126 MHz, DMSO-*d*_6_): δ (ppm) 171.8, 170.5, 152.4, 140.6, 139.9, 127.5, 126.8, 106.9, 87.8, 9.7, 9.4, 8.1, 8.1. FTIR (neat, cm^–1^): 3343, 3199, 2918, 1693, 1663. HRMS (APCI^+^) *m*/*z*: [M + H]^+^ calcd for C_13_H_17_N_2_O_3_^+^ 249.1234, found 249.1232. UV–vis (CH_3_OH): λ_max_(ε) = 277 (20 000) nm (L mol^–1^ cm^–1^). Fluorescence (CH_3_OH): λ_max_ = 424 nm, Φ_*F*_ < 0.01. Spectroscopic data are consistent with those in the literature.^35,72^

##### (*Z*)-5-((3,4-Dimethyl-5-oxo-1,5-dihydro-2*H*-pyrrol-2-ylidene)methyl)-3,4-dimethyl-2-oxo-2*H*-pyrrol-1-ium chloride (**17·HCl**)

The compound was prepared according to a slightly modified literature procedure.^35^ Propentdyopent **16a** (50.0 mg, 191 μmol) was dissolved in glacial acetic acid (2 mL) and under stirring, and an excess of diethyl ether saturated with dry HCl gas was added (20 mL). Immediately after addition, a bright yellow precipitate formed. The suspension was then kept in a refrigerator for 1 h and then filtered and washed with dry ether (2 × 10 mL). The solid was dried under reduced pressure. Yield: 47 mg (90%); bright orange-yellow powder. Mp: no melting point was observed, decomp. (darkening of a sample) at 160 °C. ^1^H NMR (300 MHz, CF_3_CO_2_D): δ (ppm) 6.30 (bs, 1H), 2.28 (bm, 12H). ^1^H NMR (300 MHz, 0.5 mL CDCl_3_ + 10 μL CF_3_CO_2_H): δ (ppm) 5.04, 2.18, 2.16, 2.16, 2.03, 2.03, 1.99, 1.95, 1.92, 1.91, 1.91, 1.86, 1.83, 1.83. FTIR (neat, cm^–1^): 2838, 2758, 2646, 1753, 1600, 1032. HRMS (APCI^+^) *m*/*z*: [M – Cl]^+^ calcd for C_13_H_15_N_2_O_2_^+^ 231.1128, found 231.1131. UV–vis (CF_3_CO_2_H): λ_max_(ε) = 400 (not determined) nm (L mol^–1^ cm^–1^). ^1^H NMR spectrum recorded in CDCl_3_ (with CF_3_CO_2_H) probably contains many rotamers (see Figure S22); increasing the temperature did not improve the spectra and led to sample degradation. ^13^C NMR could not be obtained. Spectroscopic data (^1^H NMR data) are consistent with those in the literature.^35^

##### (*Z*)-5-((3,4-Dimethyl-5-oxo-1,5-dihydro-2*H*-pyrrol-2-ylidene)methyl)-3,4-dimethyl-2-oxo-2*H*-pyrrol (**17**)

Iminium chloride **17·HCl** (9.60 mg, 36.0 μmol) was suspended in dry dichloromethane (10 mL). Then, triethylamine (4.0 eq, 20 μL, 144 μmol) was added, which caused the sudden dissolution of the suspension to give a red solution. The mixture was washed with ice-cold distilled water (2 × 30 mL) to remove the triethylamine hydrochloride, and the organic phase was dried with anhydrous Na_2_SO_4_. The solids were filtered, and the solvent was removed under reduced pressure. Yield: 7 mg (84%); brick red solid. Mp: 205–208 °C, decomp. ^1^H NMR (500 MHz, CD_2_Cl_2_): δ (ppm) 10.27 (bs, 1H), 5.79 (s, 1H), 2.08 (q, *J* = 1.4 Hz, 6H), 1.91 (q, *J* = 1.4 Hz, 6H). ^13^C{^1^H} NMR (126 MHz, CD_2_Cl_2_): δ (ppm) 180.5, 171.9, 143.4, 134.4, 92.1, 10.3, 9.1. FTIR (neat, cm^–1^): 1711, 1604, 1060. HRMS (APCI^+^) *m*/*z*: [M + H]^+^ calcd for C_13_H_15_N_2_O_2_^+^ 231.1128, found 231.1128. UV–vis (CH_2_Cl_2_): λ_max_(ε) = 360 (14 800) nm (L mol^–1^ cm^–1^). This compound was reported before, however, only elemental analysis and melting points were reported.^101,102^

##### (*Z*)-5-((2-Amino-3,4-dimethyl-5-oxo-2,5-dihydrofuran-2-yl)methylene)-3,4-dimethyl-1,5-dihydro-2H-pyrrol-2-one (**19**)

*Z*-**13** (58.0 mg, 268 μmol) was dissolved in dry dichloromethane (60 mL), the mixture was cooled to −15 °C, and then 3-chloroperbenzoic acid (*m*-CPBA, 77.0%, 109 mg, 485 μmol) was added in one portion. The mixture was stirred for 30 min, while the temperature was allowed to rise to 0 °C. Then, the mixture was washed with aq. NaHCO_3_ (1%, 2 × 120 mL), dried with anhydrous Na_2_SO_4_, and the solvent was removed under reduced pressure to give a pale-yellow solid (40 mg). The crude residue was purified two times by reverse-phase flash chromatography with gradient elution (20 → 80% methanol in aq ammonia, 0.05% NH_3_). Due to the close retention factors of the reaction products, it was not possible to fully separate the product from propentdyopent **16b**, also produced in the reaction. Yield: 13 mg; white solid. The mixture contains the title compound **19** and **16b** as main products in the molar ratio of ∼2:1. See the NMR data in Figures S15–S20, HPLC data in Figure S35 and HPLC-MS data in Figure S38. ^1^H NMR (500 MHz, DMSO-*d*_*6*_): δ (ppm) 8.90 (s, 1H, > N*H*), 5.00 (s, 1H, ≥ C*H*), 3.57 (s, 2H, – N*H*_*2*_), 1.94 (s, 3H), 1.86 (s, 3H), 1.77 (s, 3H), 1.72 (s, 3H). ^13^C{^1^H}NMR (126 MHz, DMSO-*d*_*6*_): δ (ppm) 171.9, 170.6, 158.6, 140.8, 140.6, 128.3, 123.5, 105.5, 98.3, 11.0, 9.4, 8.6, 8.2. HRMS (APCI^+^) *m*/*z*: [M + H]^+^ calcd for C_13_H_17_N_2_O_3_^+^ 249.1234, found 249.1231 (see Figure S61).

##### 1,4-Dimethyl-1,4-dihydro-1,4-epidioxynaphthalene (DMNO_2_)

1,4-Dimethylnaphthalene (2.10 g, 13.4 mmol, NMR/1) was charged in a long cylindrical flask with a mixture of dichloromethane and methanol (250 mL, 3:1, v/v). Then, rose bengal (∼5 mg) or methylene blue (∼5 mg) as a photosensitizer was predissolved in methanol (10 mL) and added to the mixture to obtain a deep violet solution. Then, the solution was irradiated with three 100 W white LED reflectors under vigorous stirring while purging the solution with oxygen gas and cooling the bottom part in an ethanol/dry ice bath. The working setup is displayed in Figure S63. The temperature of the reaction mixture was monitored and maintained between 2–15 °C. TLC was recorded regularly (50% dichloromethane in hexane, v/v). The mixture was irradiated until almost full conversion (TLC) was reached (8 h). The solvent was evaporated under reduced pressure, while maintaining the temperature of the heating bath at ∼18 °C. The residue was purified by silica-gel column chromatography using a precooled mobile phase (∼0 °C) to prevent thermal decomposition (50 → 100% dichloromethane in hexane, v/v). The residue was reprecipitated from the mixture of dichloromethane and hexane (1:1, v/v) on an evaporator to produce a white crystalline fluffy powder, which was filtered and washed with cold hexane. The product was quickly (5 min) dried under reduced pressure to remove hexane and then stored in the freezer. Yield: 1.26 g (49.8%); fine crystalline powder. ^1^H NMR (300 MHz, CDCl_3_): δ (ppm) 7.36–7.23 (m, 4H), 6.69 (s, 2H), 1.88 (s, 6H). ^13^C{^1^H} NMR (75 MHz, CDCl_3_): δ (ppm) 141.1, 139.3, 126.8, 120.2, 78.8, 16.3. Spectroscopic data are consistent with those in the literature.^103^

## Data Availability

The data underlying this study is available in the published article and Supporting Information.

## Supporting Information Available

Quantum yield measurements; NMR and optical spectra; HPLC, HPLC-MS, HPLC-UV–vis and HRMS data; kinetic data; experimental setups; optimized geometries (PDF)
