# Supplementary material for: Photooxidation of Dipyrrinones: Reaction with Singlet Oxygen and Characterization of Reaction Intermediates
Source: J Org Chem. 2025 Feb 5;90(6):2403–20. doi: 10.1021/acs.joc.4c02954 (PMC11833878; doi:10.1021/acs.joc.4c02954)
Supplement: Supplementary file 1 — jo4c02954_si_001.pdf [file jo4c02954_si_001.pdf]

Supporting Information

Photooxidation of Dipyrrinones: Reaction with Singlet Oxygen and Characterization of Reaction Intermediates

Dominik Madea,[†,] Júlia Peňáková,[†] Jaya Mehara,[‡] Rikuo Akisaka,[†] Marek Martínek,[†] Jana Roithová [‡,*] and Petr Klán[†,*]*

[†] Department of Chemistry and RECETOX, Faculty of Science, Masaryk University, Kamenice 5, 625 00, Brno, Czech Republic.

[‡] Faculty of Science, Radboud University, Heyendaalseweg 135, 6525 AJ Nijmegen, Netherlands.

* dominik.madea@seznam.cz, j.roithova@science.ru.nl, klan@sci.muni.cz

Contents

Isomerization Quantum Yields of **13** S2
NMR Data S3
HPLC, HPLC-MS and UV-Vis Data S29
Mass Spectroscopy Data S55
Kinetic Model Data S64
Determination of Quenching Rates S70
HRMS Data S72
Experimental Setup and LED Spectra S74
Optimized Geometries S77

Configurational Isomerization and Quantum Yields. As anticipated,[1-3] irradiation of either (*Z*) or (*E*)- isomer of **13** in organic solvents of different polarity (MeOH, MeCN, toluene, or hexane) led to efficient isomerization and formation of the photostationary state, which consisted of the mixture of both isomers. Upon irradiation at 400 nm, the composition of both isomers was ~1:1. We determined the isomerization quantum yields (Φ_{ZE} and Φ_{EZ}) in methanol at 400 and 420 nm LEDs (see Figure S64) according to our previous method.[1] Both Φ_{ZE} and Φ_{EZ} were in the range of 0.23–0.28, comparable to the isomerization quantum yield of the bilirubin subunits studied before.[1,3]

Table S1. Wavelength-Dependent Quantum Yields of Isomers of **13** in Methanol[a]

λ_{irr} [b]/ nm	Φ_{ZE}	Φ_{EZ}
400	0.239 ± 0.013	0.253 ± 0.013
420	0.256 ± 0.019	0.279 ± 0.017

[a] Quantum yields obtained from global fitting using an HS-MCR method. Uncertainties are reported as one standard deviation. [b] The wavelengths corresponding to the maximum intensities in the emission spectra for the given LED source (Figure S64).

Figure S1. ^{1}H NMR (300 MHz, $CDCl_3$): 3,4-dimethylpyrrole.

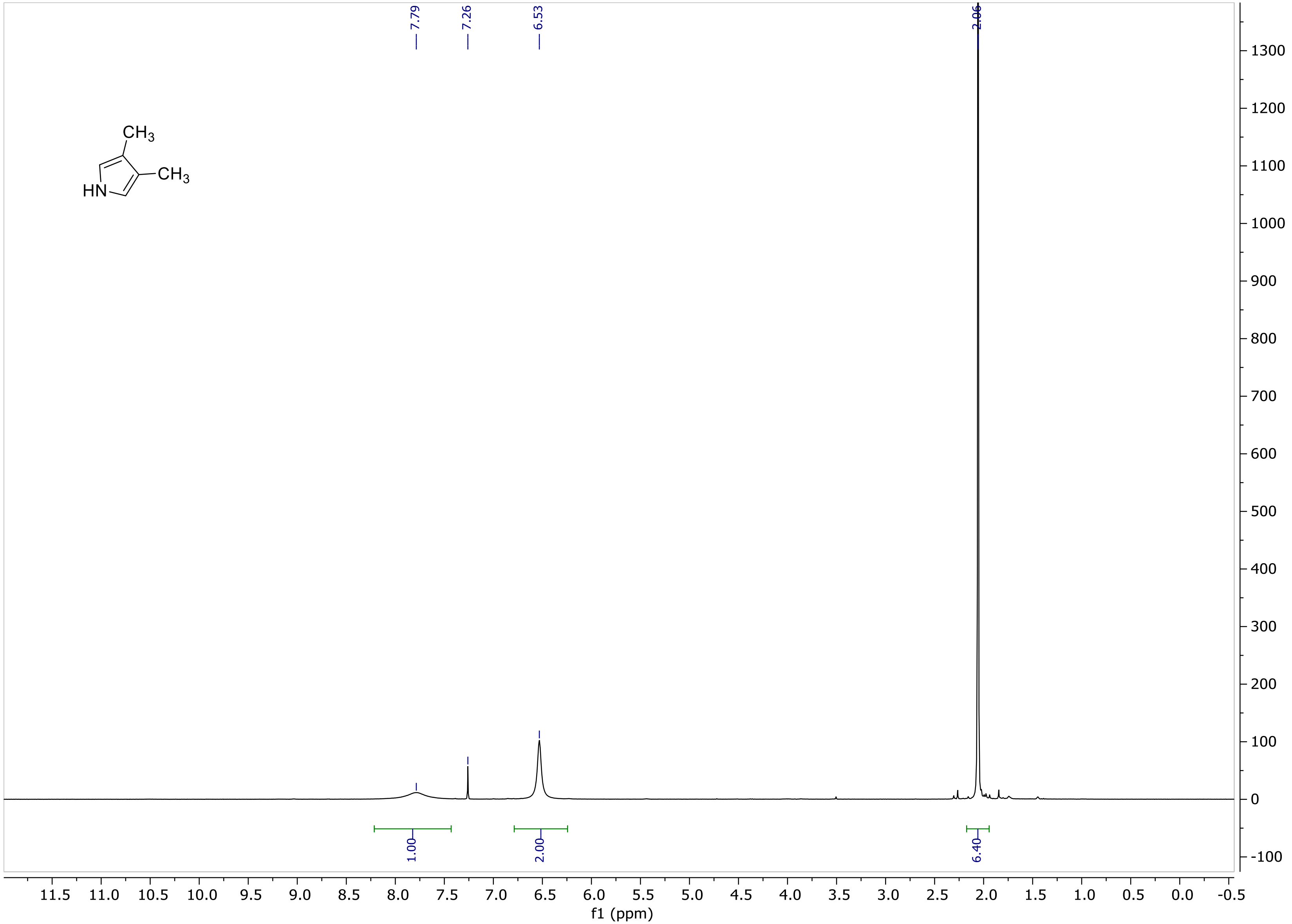

Figure S2. ^{1}H NMR (300 MHz, $CDCl_3$): 3,4-Dimethyl-1*H*-pyrrole-2-carbaldehyde (**14**).

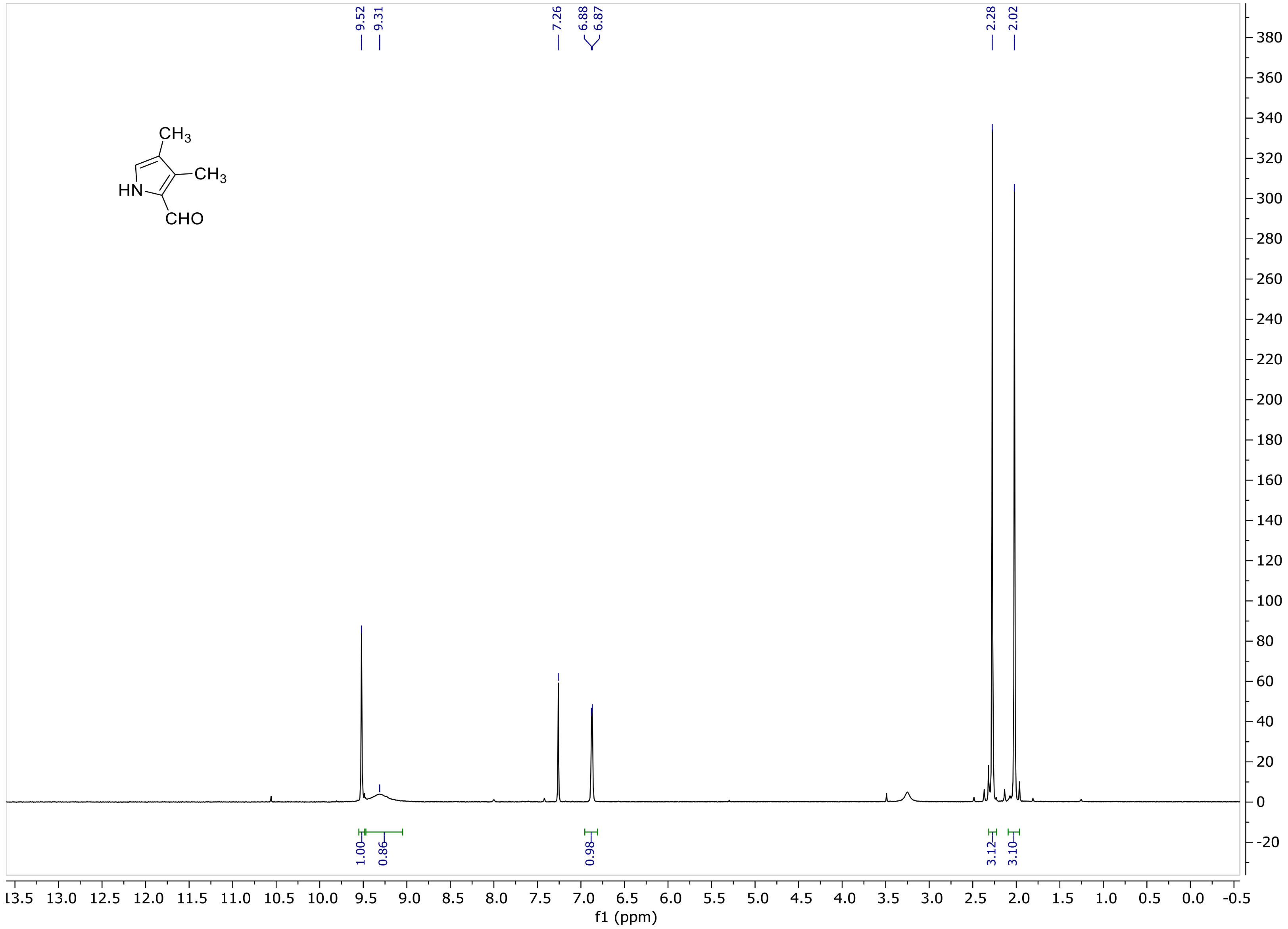

Figure S3. ^{1}H NMR (300 MHz, $CDCl_3$): 3,4-Dimethyl-1,5-dihydro-2*H*-pyrrol-2-one (**15**).

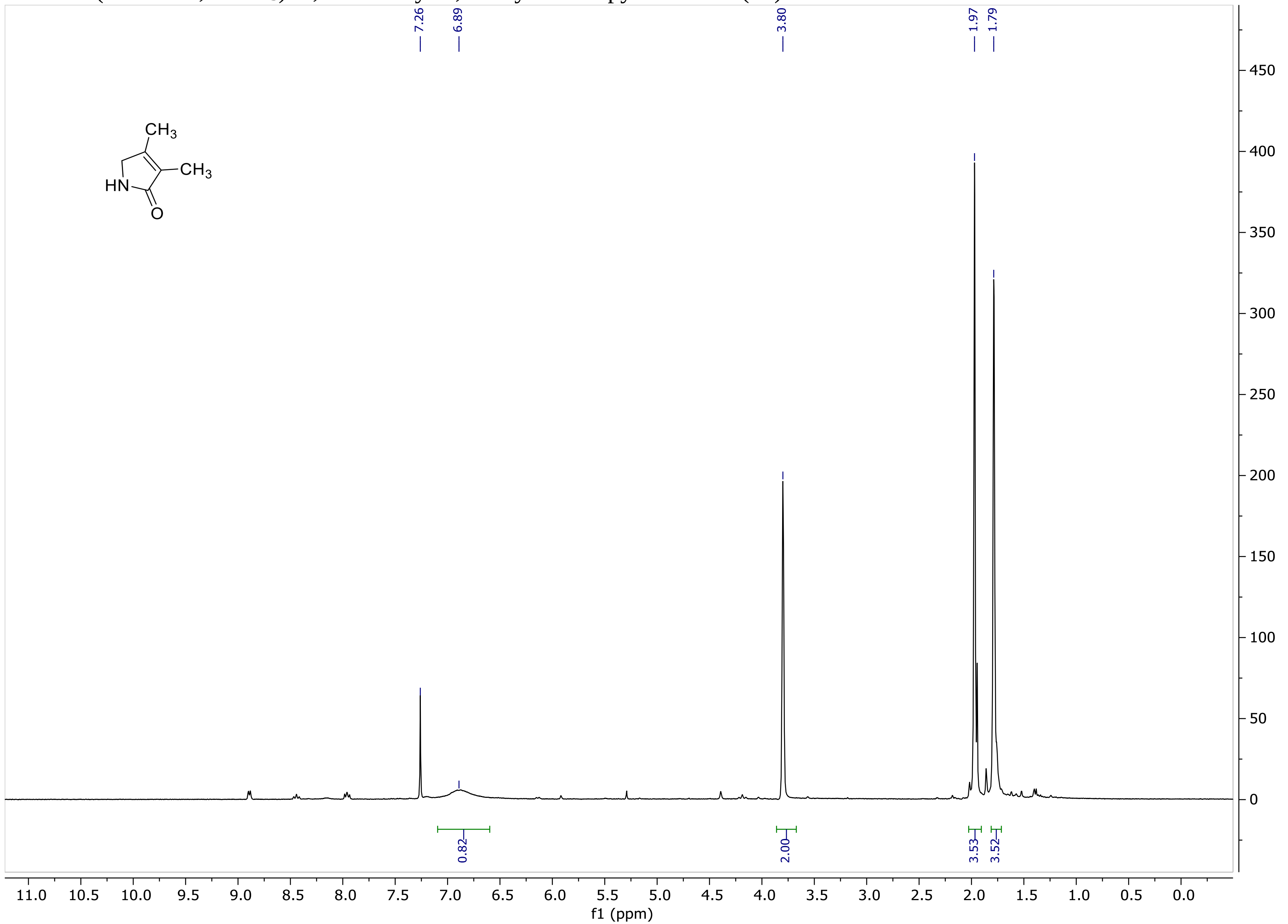

Figure S4. ^{1}H NMR (500 MHz, DMSO-d_6): *Z*-**13**.

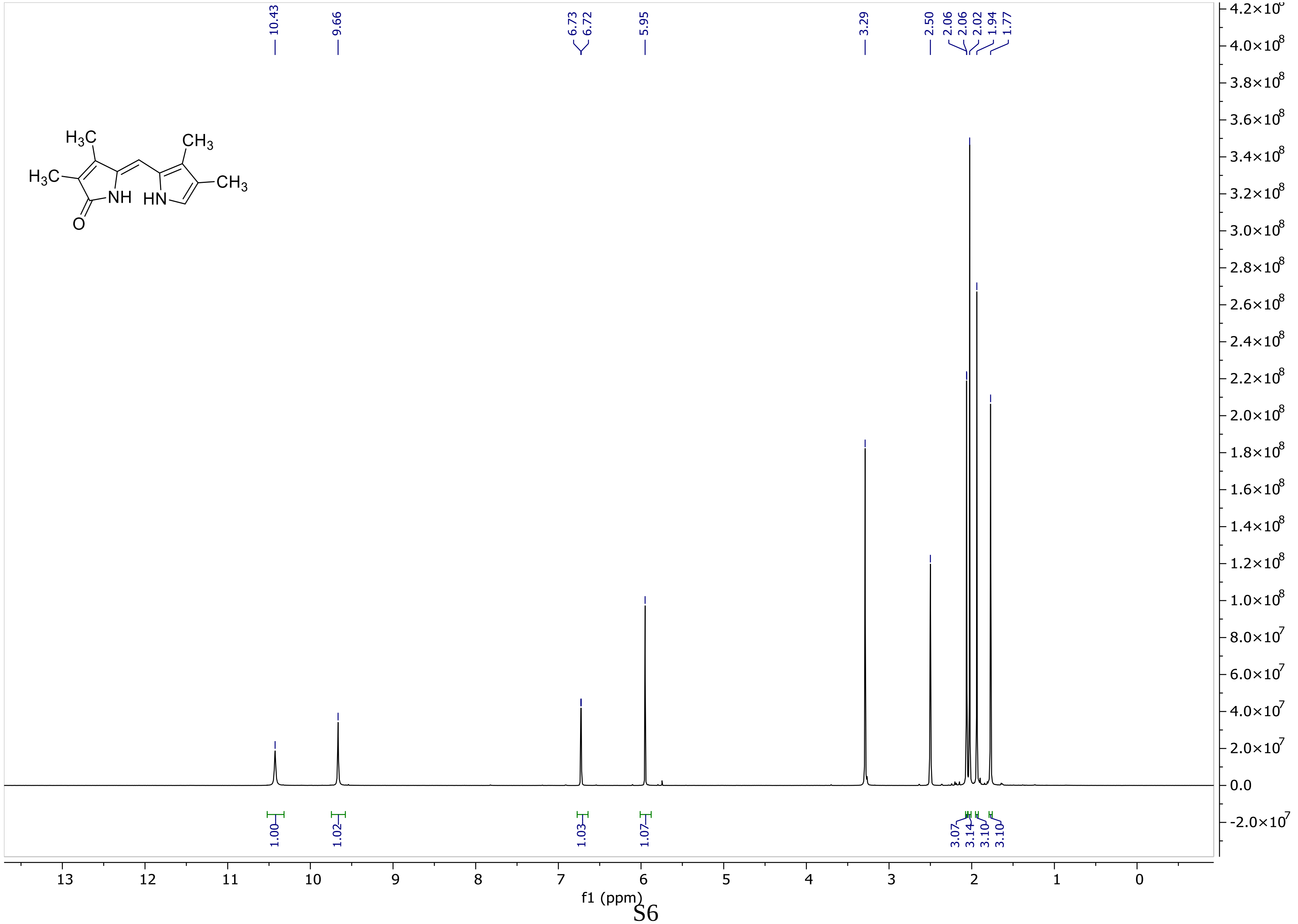

Figure S5. ${}^{13}C\{{}^{1}H\}$ NMR (126 MHz, DMSO-d_6): *Z*-**13**.

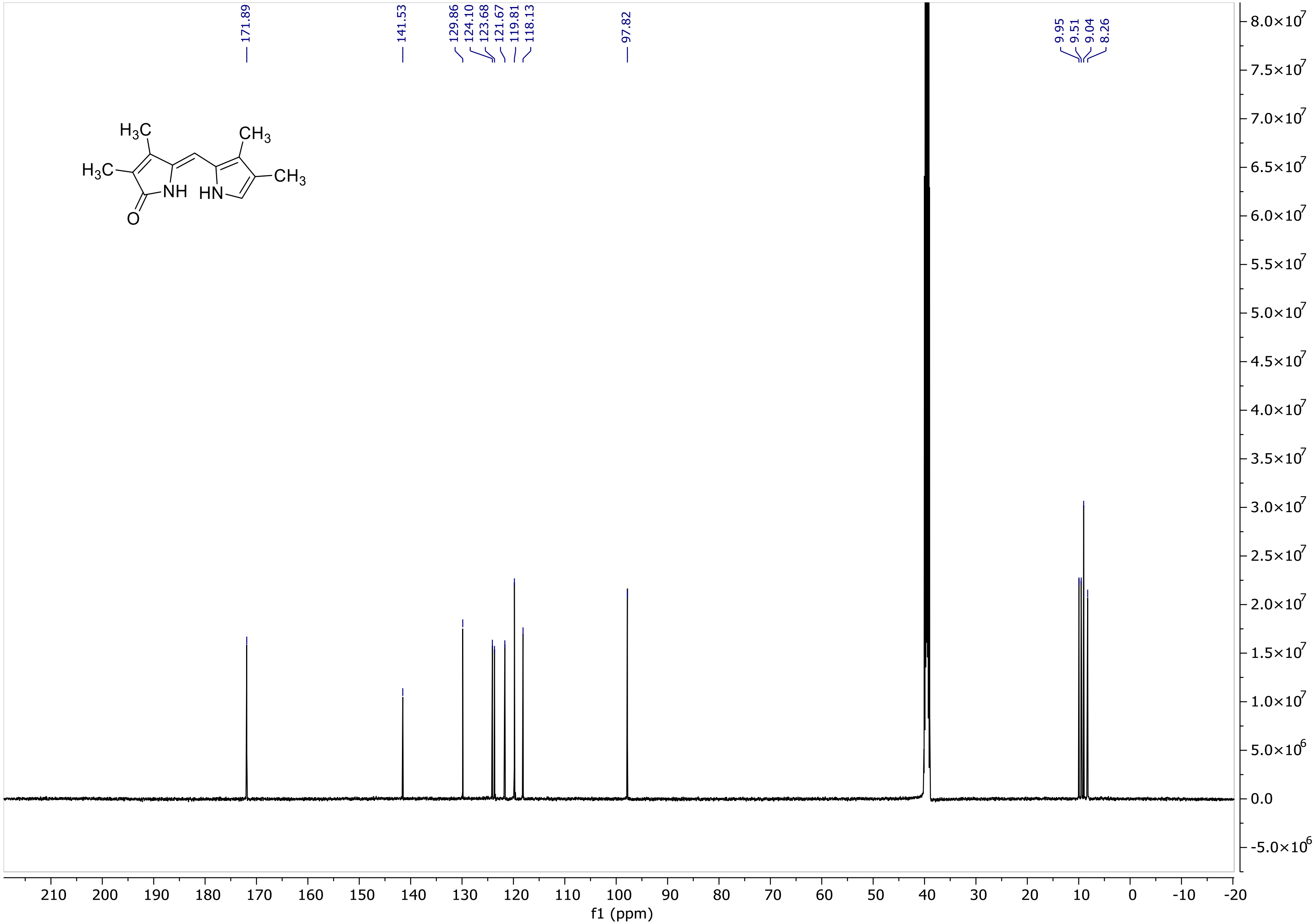

Figure S6. ^{1}H NMR (500 MHz, DMSO-d_6): *E*-**13**.

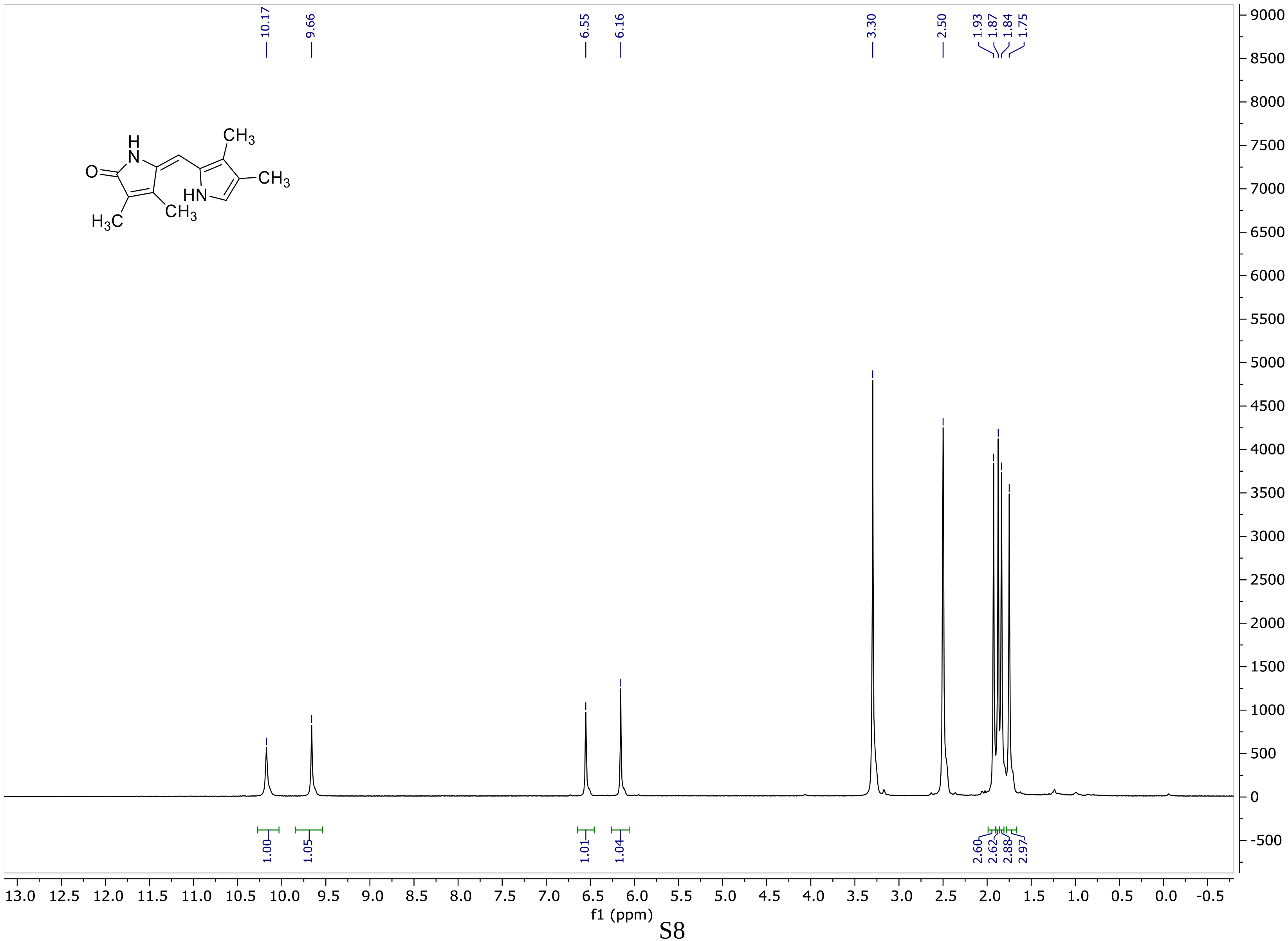

Figure S7. $^{13}C\{^{1}H\}$ NMR (126 MHz, DMSO-d_6): *E*-**13**.

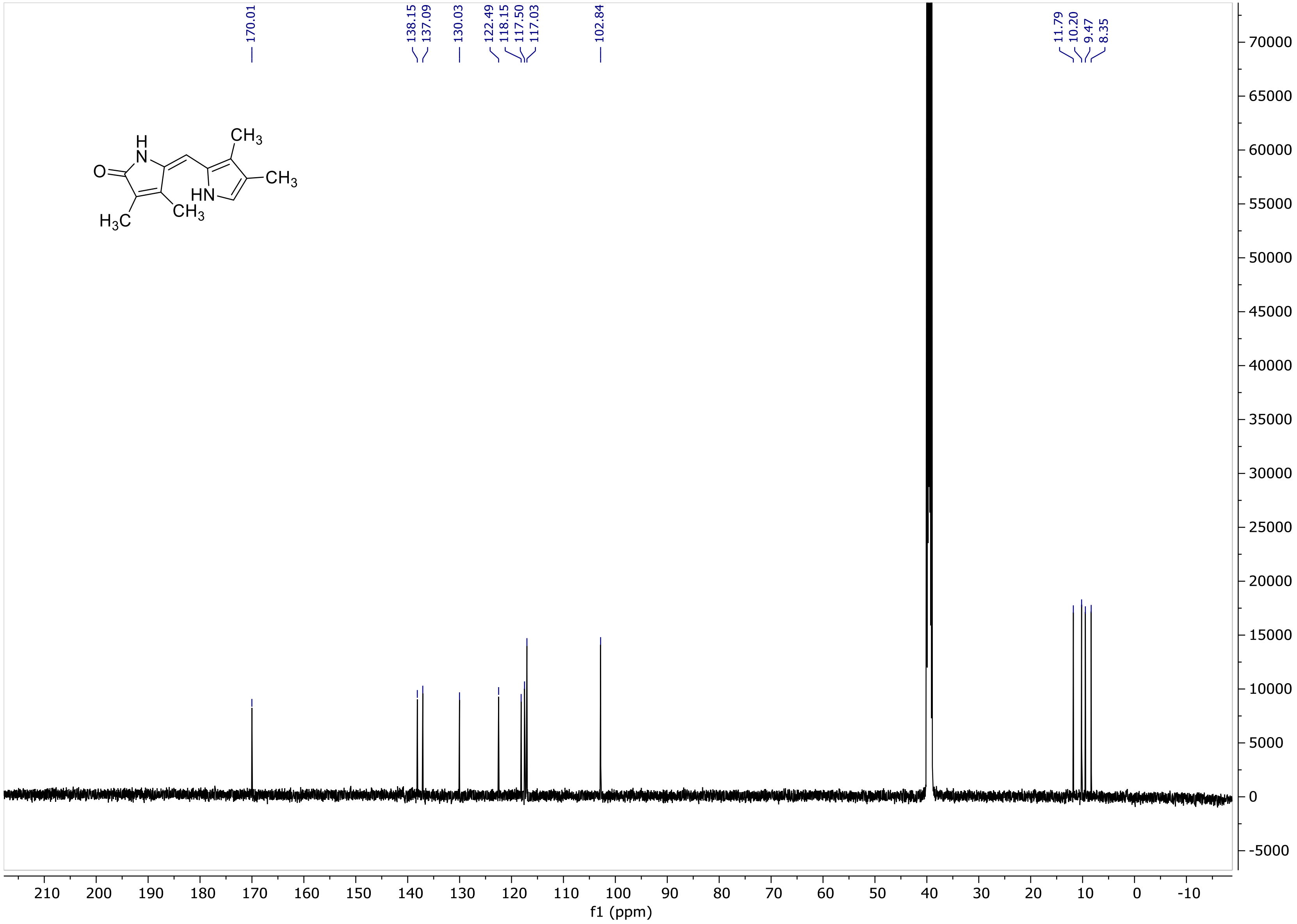

Figure S8. ^{1}H NMR (500 MHz, CD_2Cl_2): **16a**.

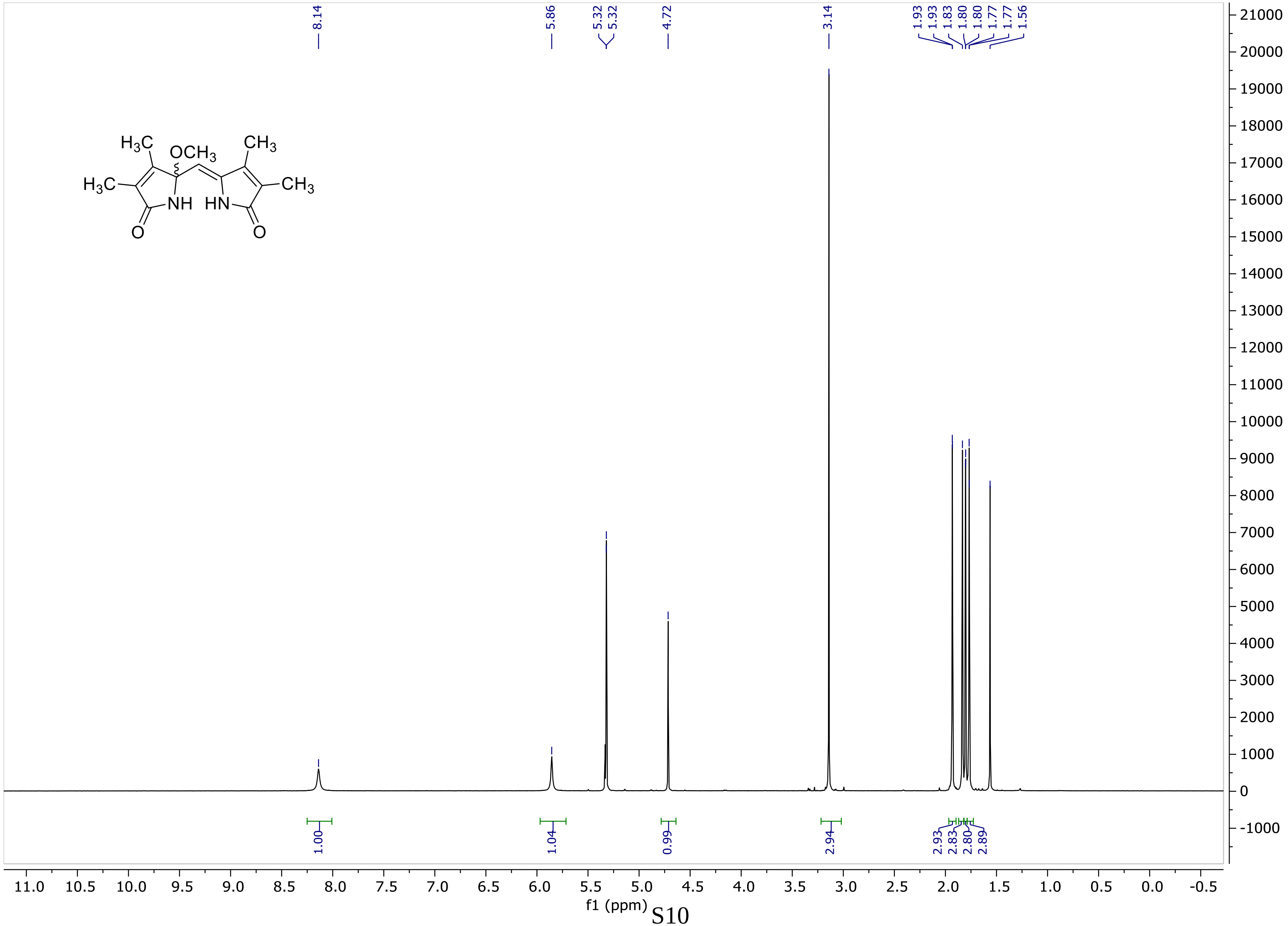

Figure S9. $^{13}C\{^{1}H\}$ NMR (126 MHz, CD_2Cl_2): **16a**.

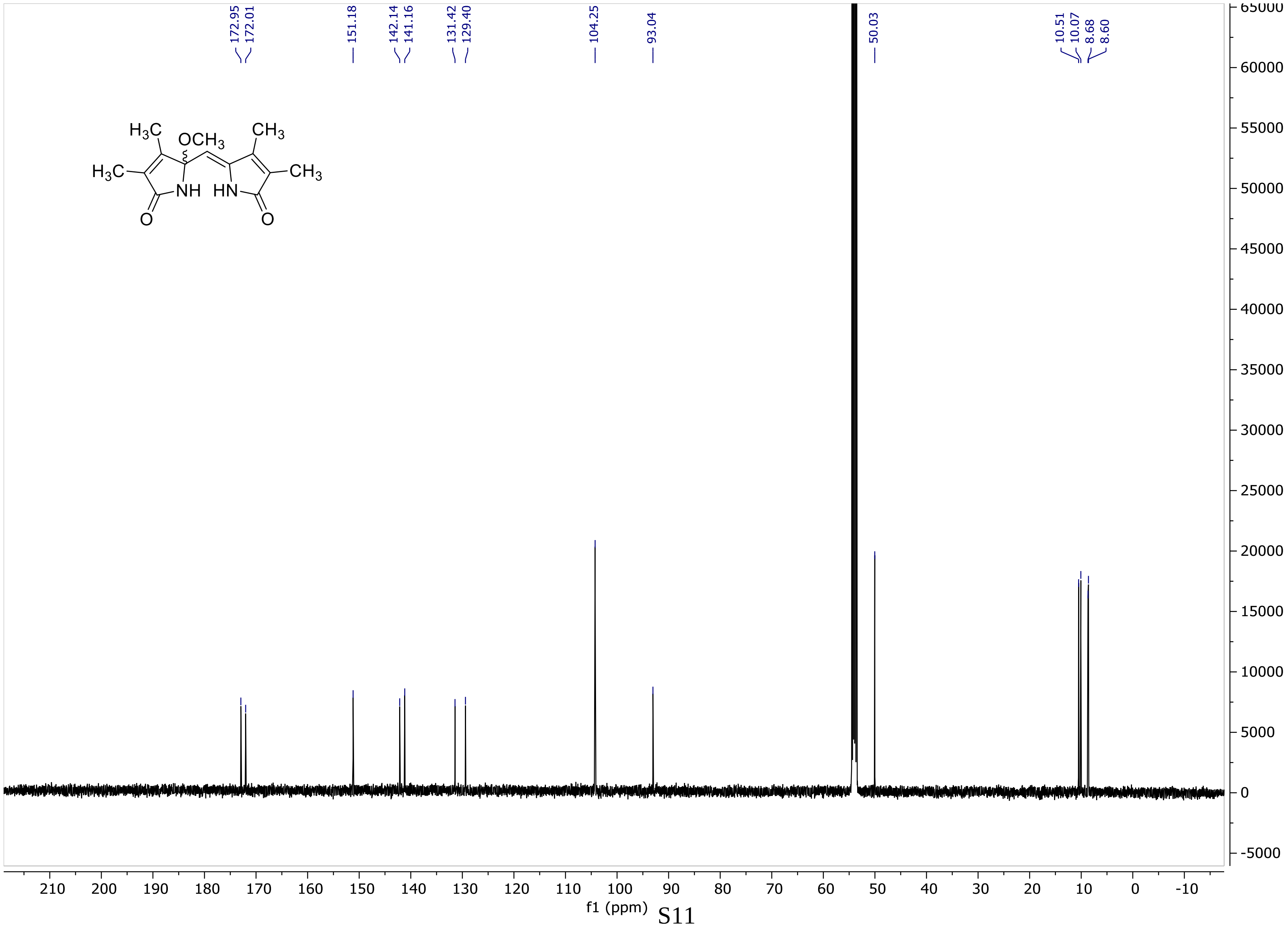

Figure S10. ^{1}H NMR (500 MHz, DMSO-d_6): **16b**.

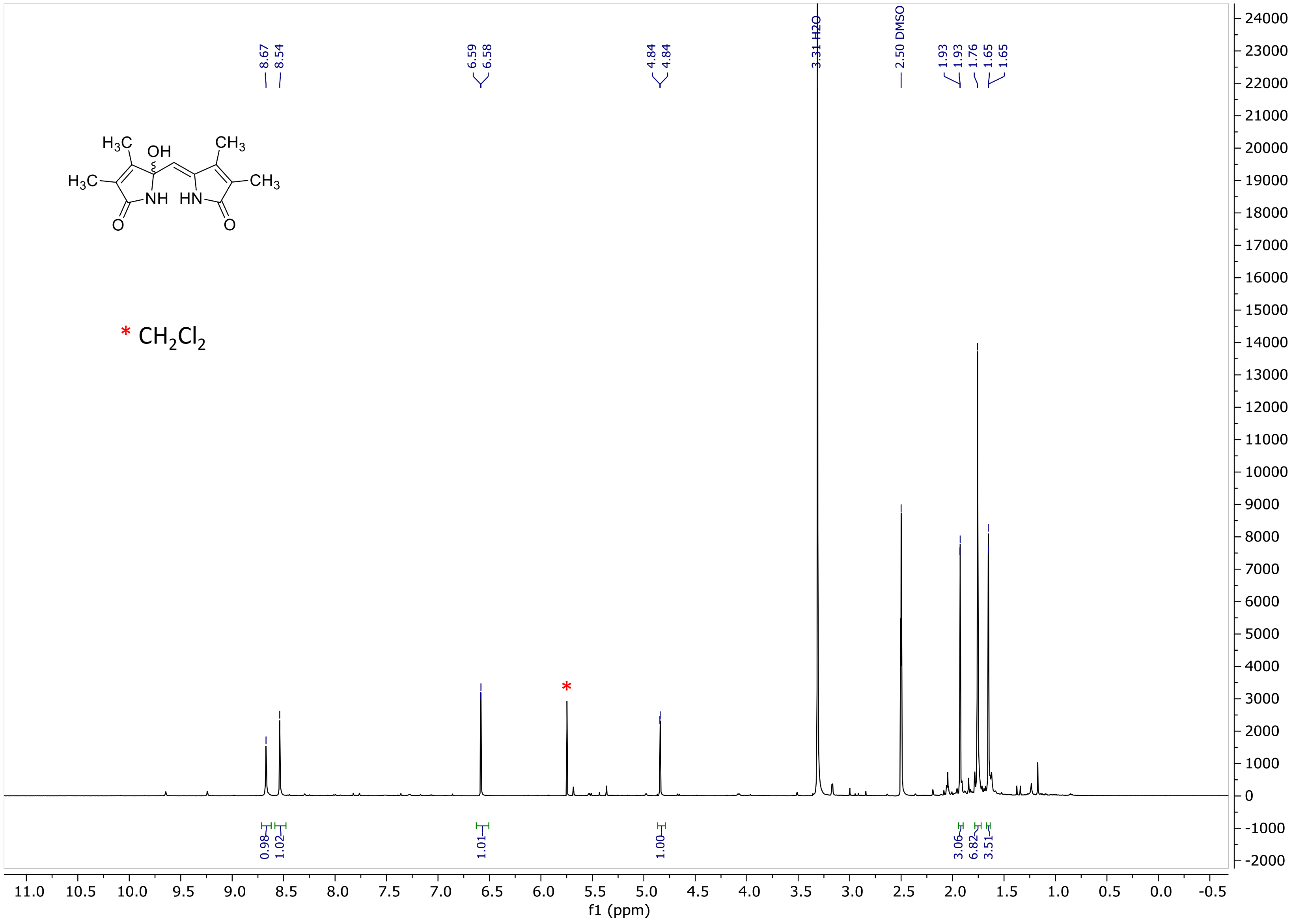

Figure S11. $^{13}C\{^{1}H\}$ NMR (126 MHz, DMSO-d_6): **16b**.

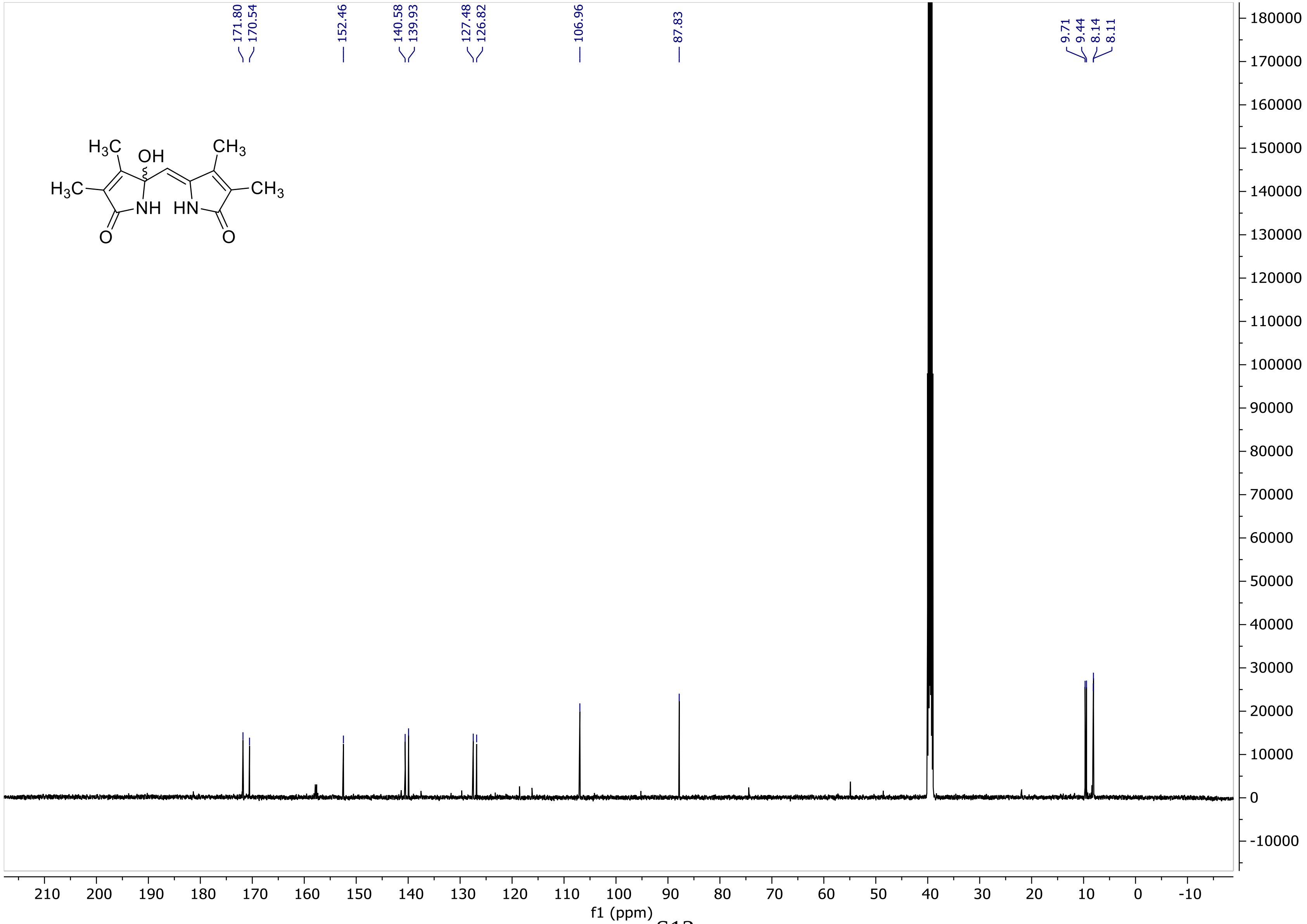

Figure S12. ^{1}H–^{1}H COSY (500 MHz, DMSO-d_6): **16b**.

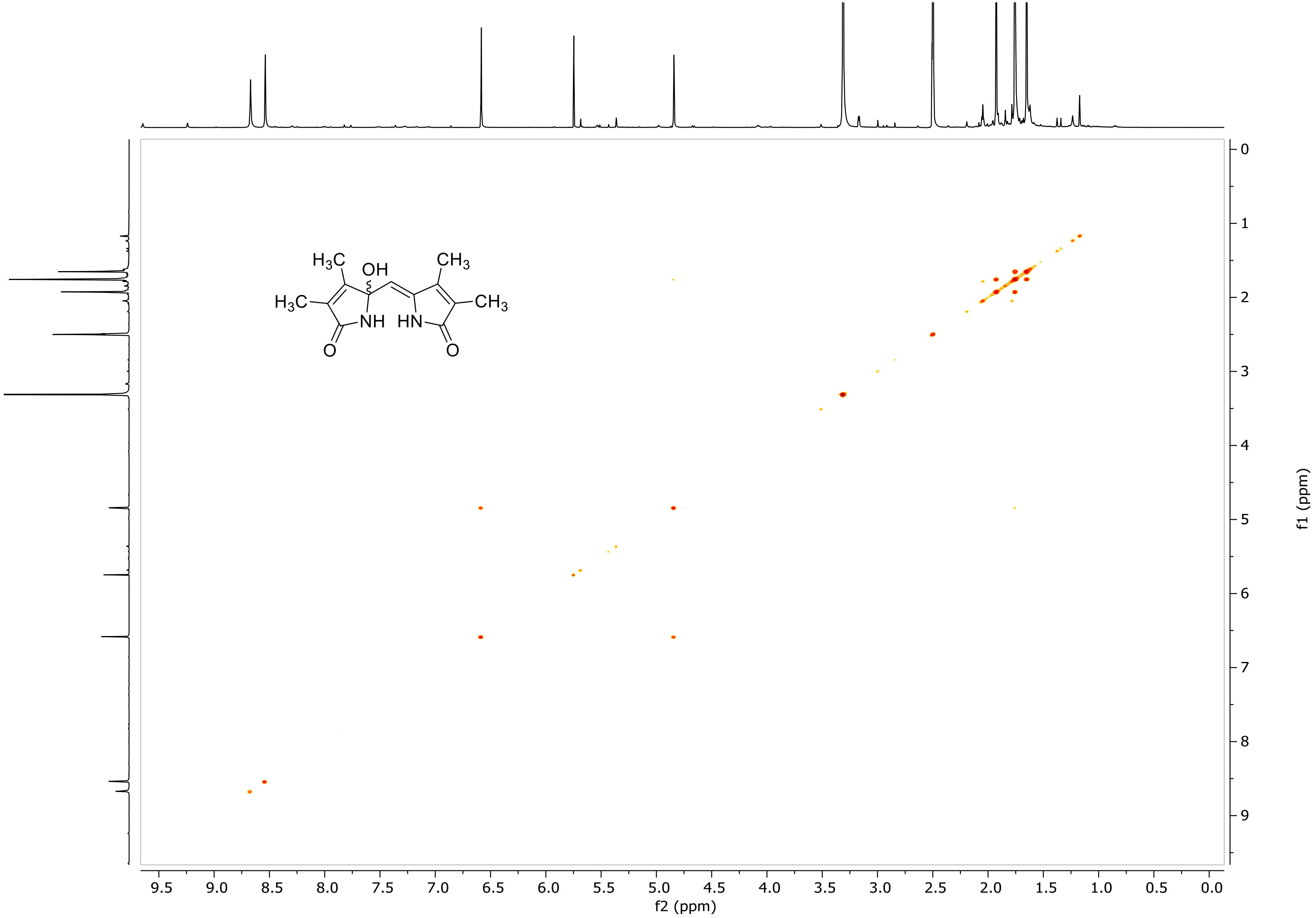

Figure S13. ^{1}H–^{13}C HSQC (500 MHz, DMSO-d_6): **16b**.

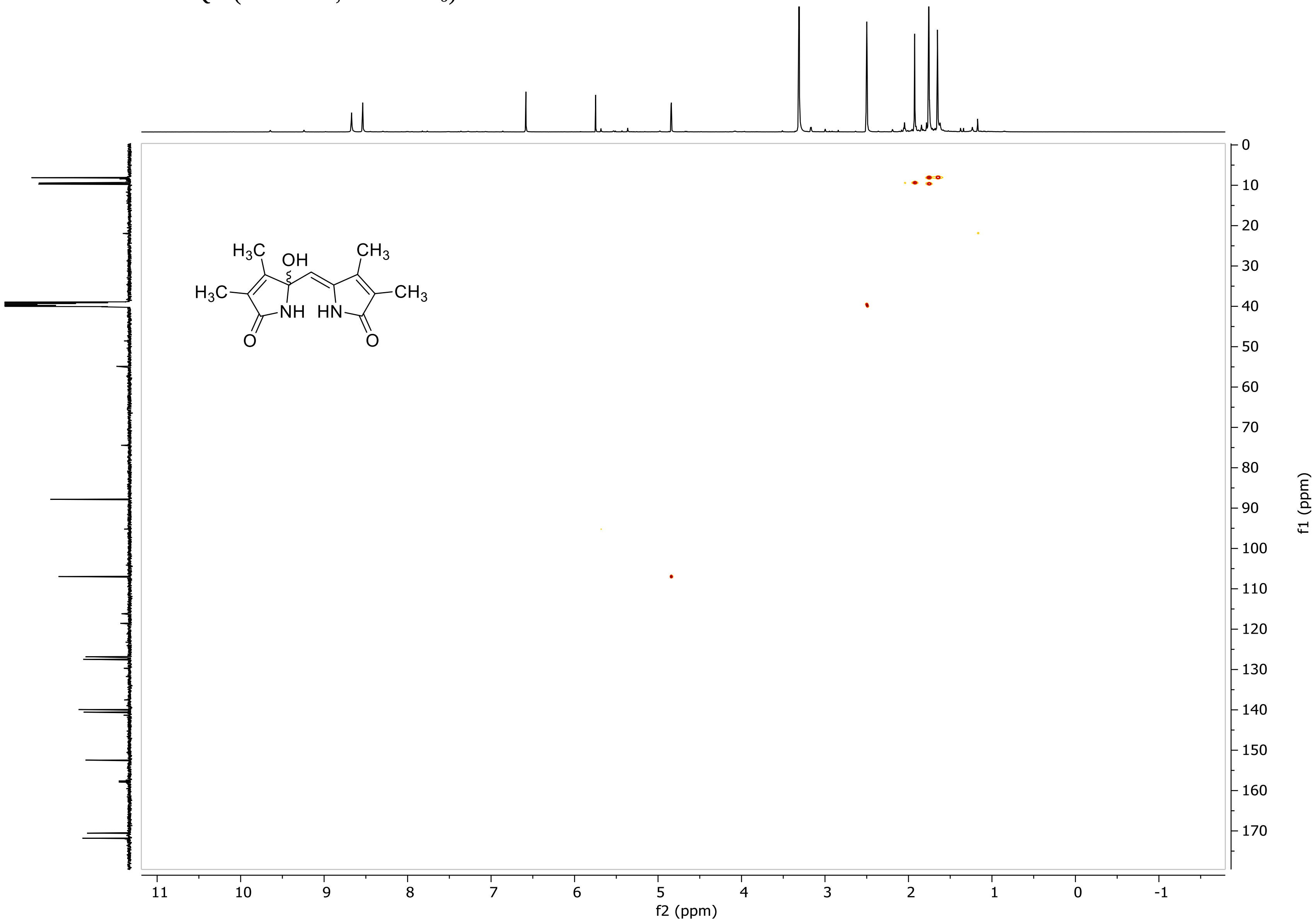

Figure S14. ^{1}H–^{13}C HMBC (500 MHz, DMSO-d_6): **16b**.

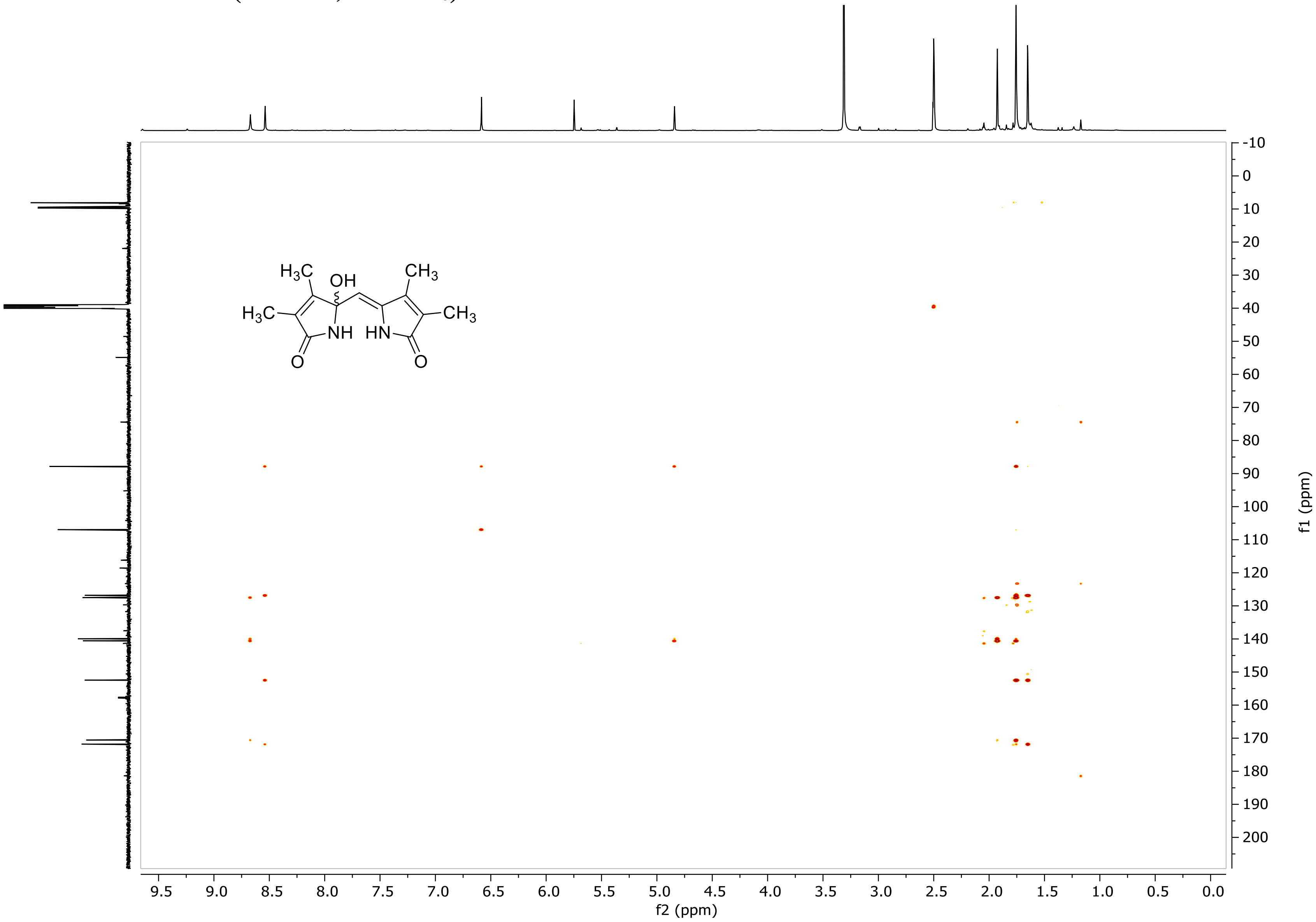

Figure S15. ^{1}H NMR (500 MHz, DMSO-d_6): A mixture of **19** and **16b** in the ratio of ~2:1. The mixture also contains a small amount of unknown product **S4b**; see Figure S35 for HPLC data.

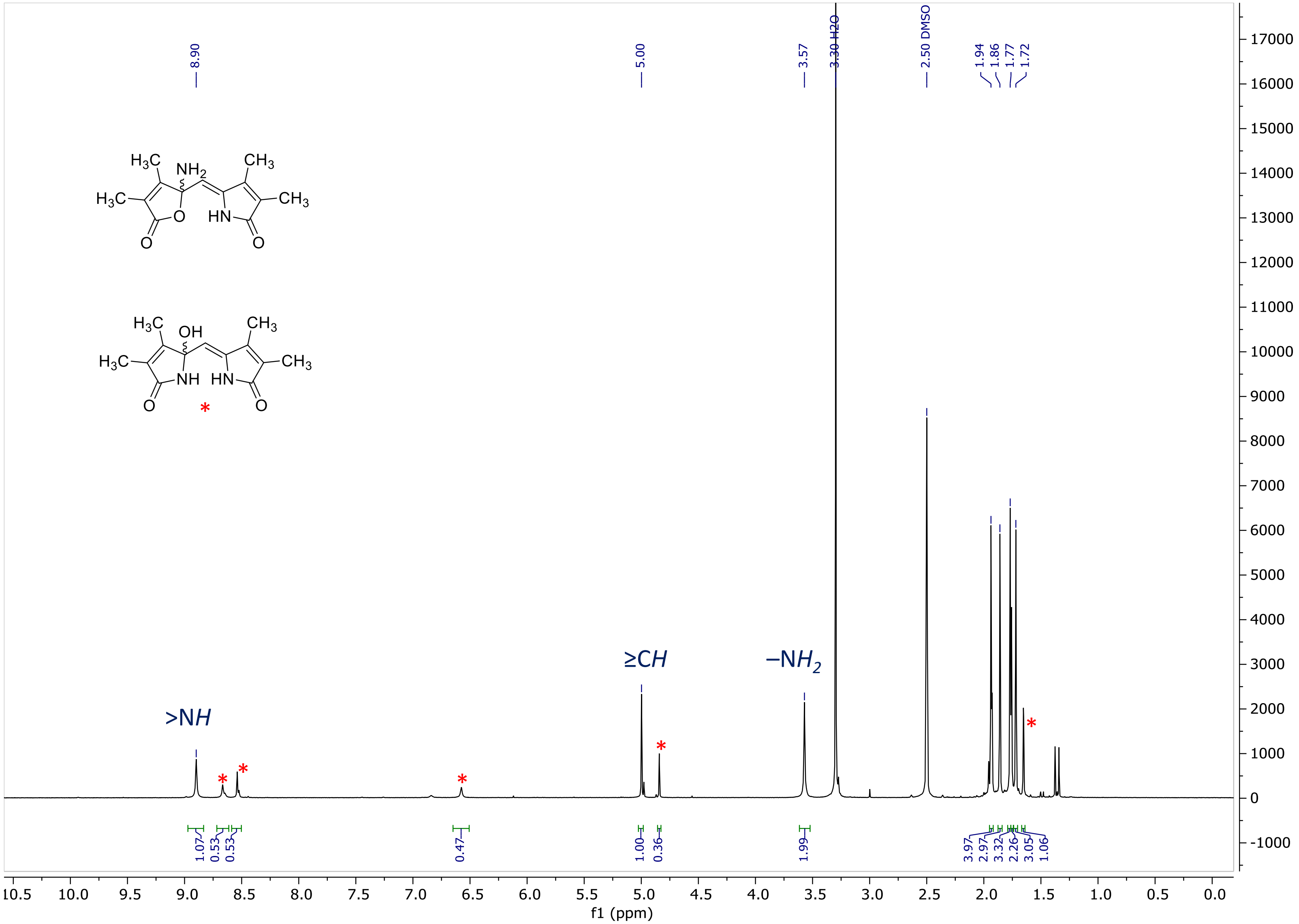

Figure S16. $^{13}C\{^1H\}$ NMR (126 MHz, DMSO-d_6): A mixture of **19** and **16b** in the ratio of ~2:1. The mixture also contains a small amount of unknown product **S4b**; see Figure S35 for HPLC data.

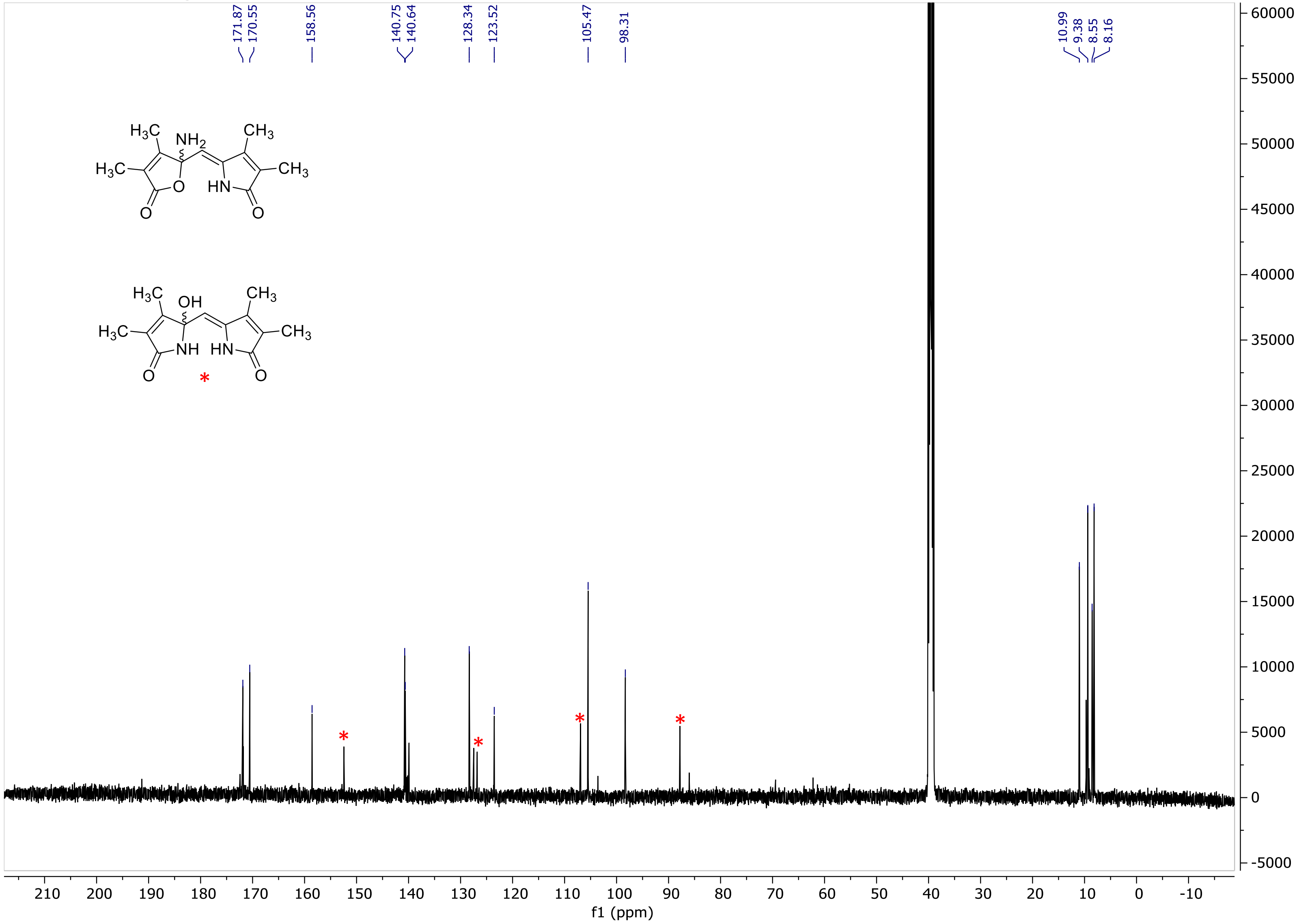

Figure S17. ^1H–^1H COSY (500 MHz, DMSO-d_6): A mixture of **19** and **16b** in the ratio of ~2:1.

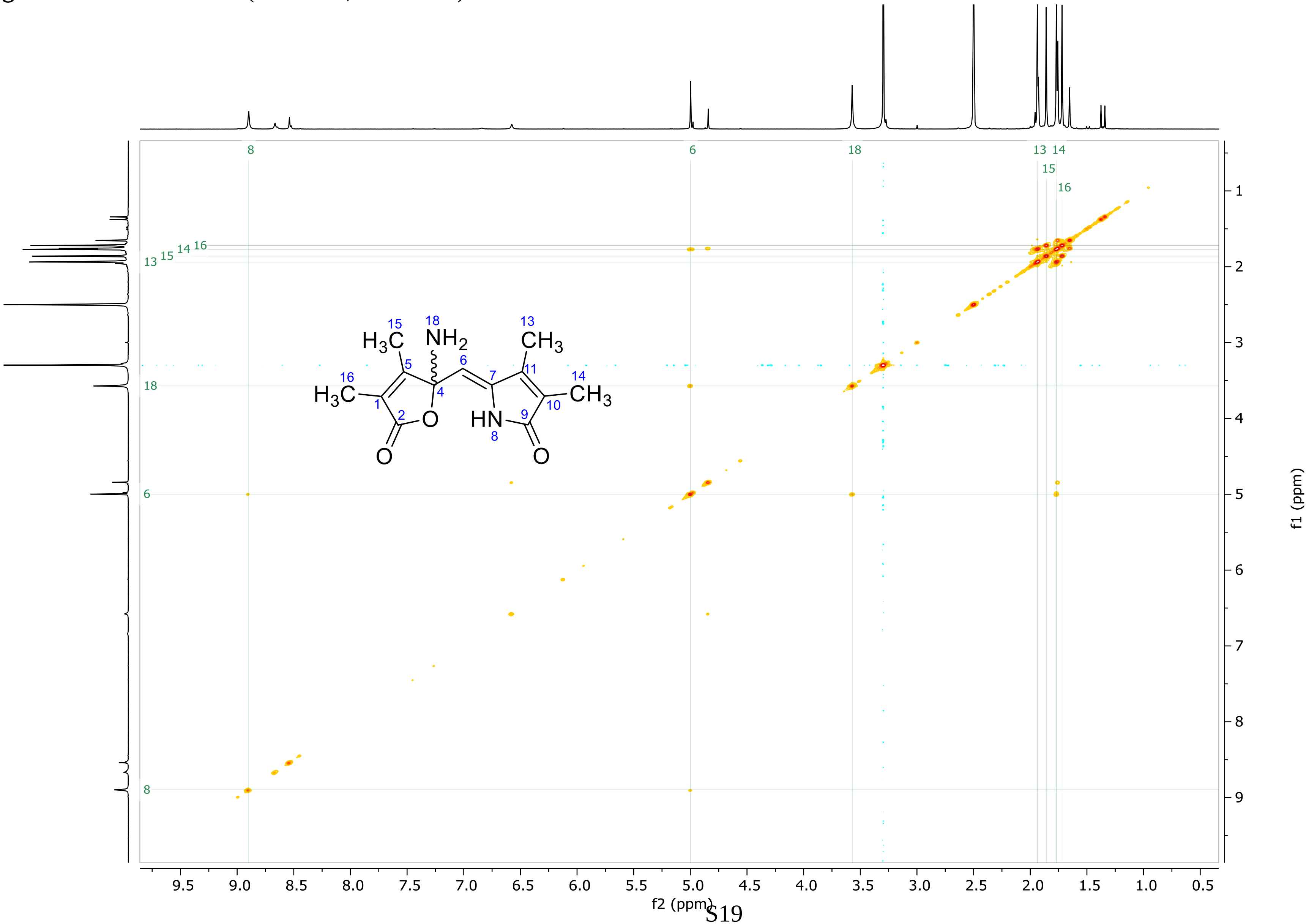

Figure S18. ^{1}H–^{1}H NOESY (500 MHz, DMSO-d_6): A mixture of **19** and **16b** in the ratio of ~2:1.

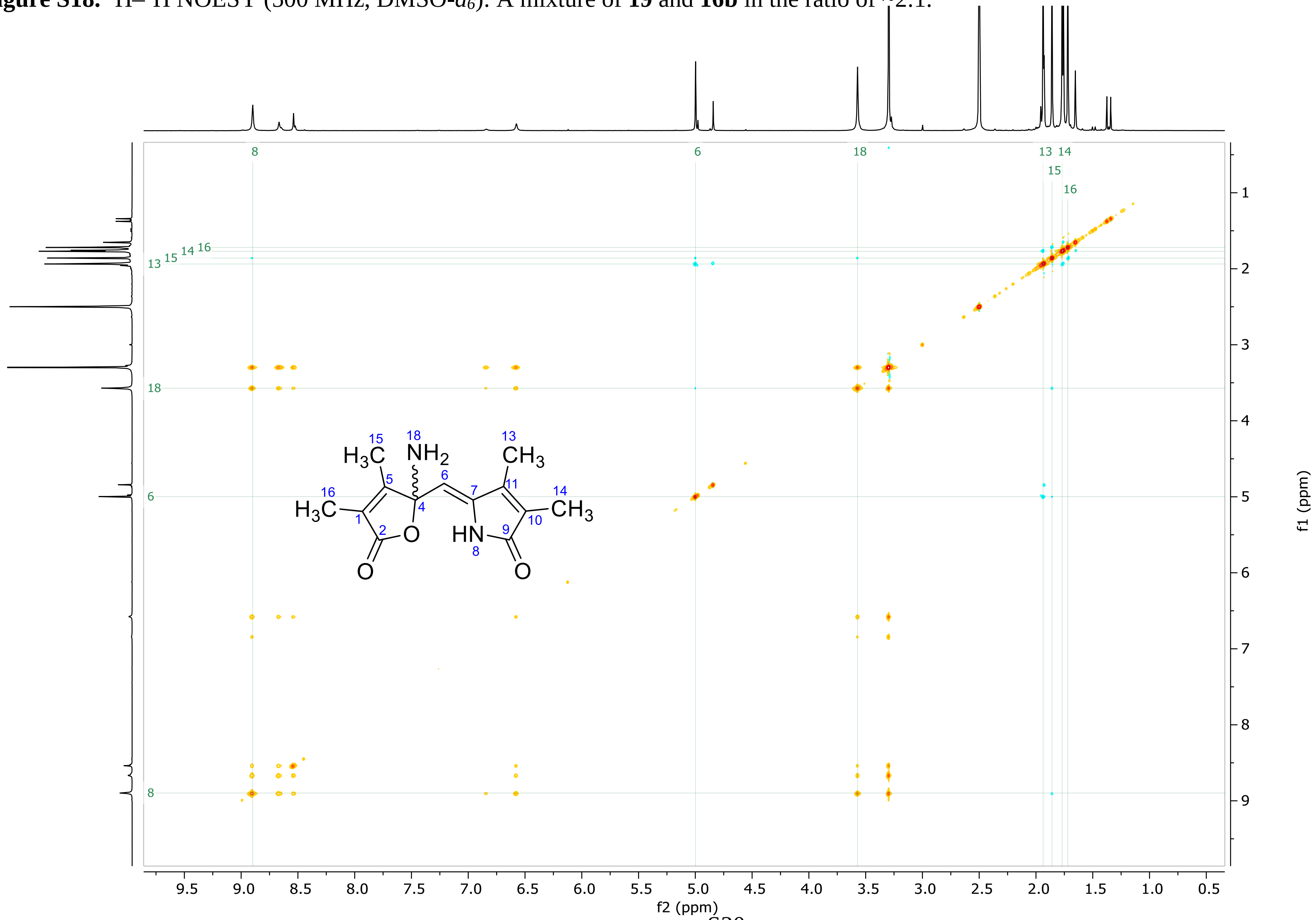

Figure S19. ^{1}H–^{13}C HSQC (500 MHz, DMSO-d_6): A mixture of **19** and **16b** in the ratio of ~2:1.

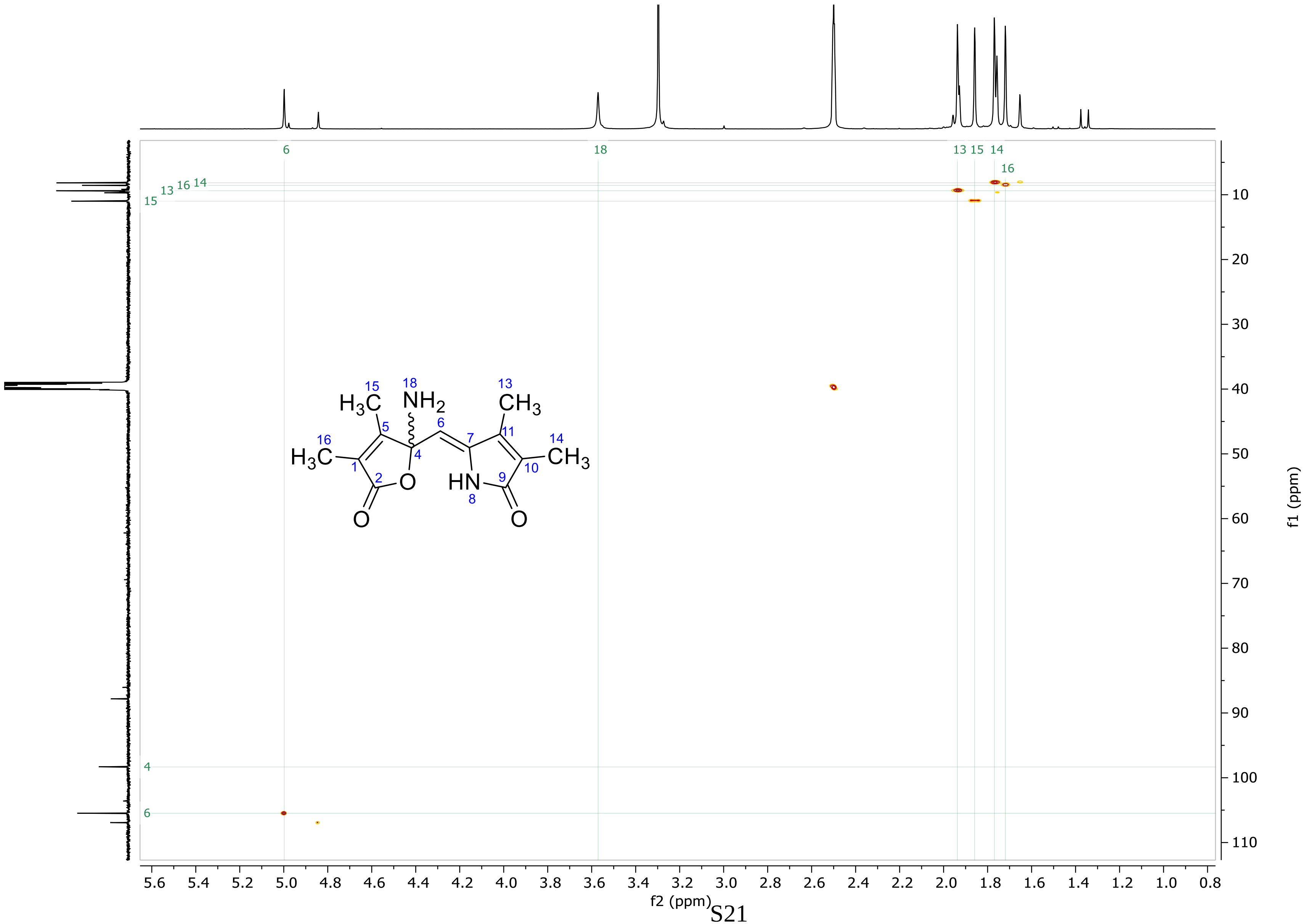

Figure S20. ^{1}H–^{13}C HMBC (500 MHz, DMSO-d_6): A mixture of **19** and **16b** in the ratio of ~2:1.

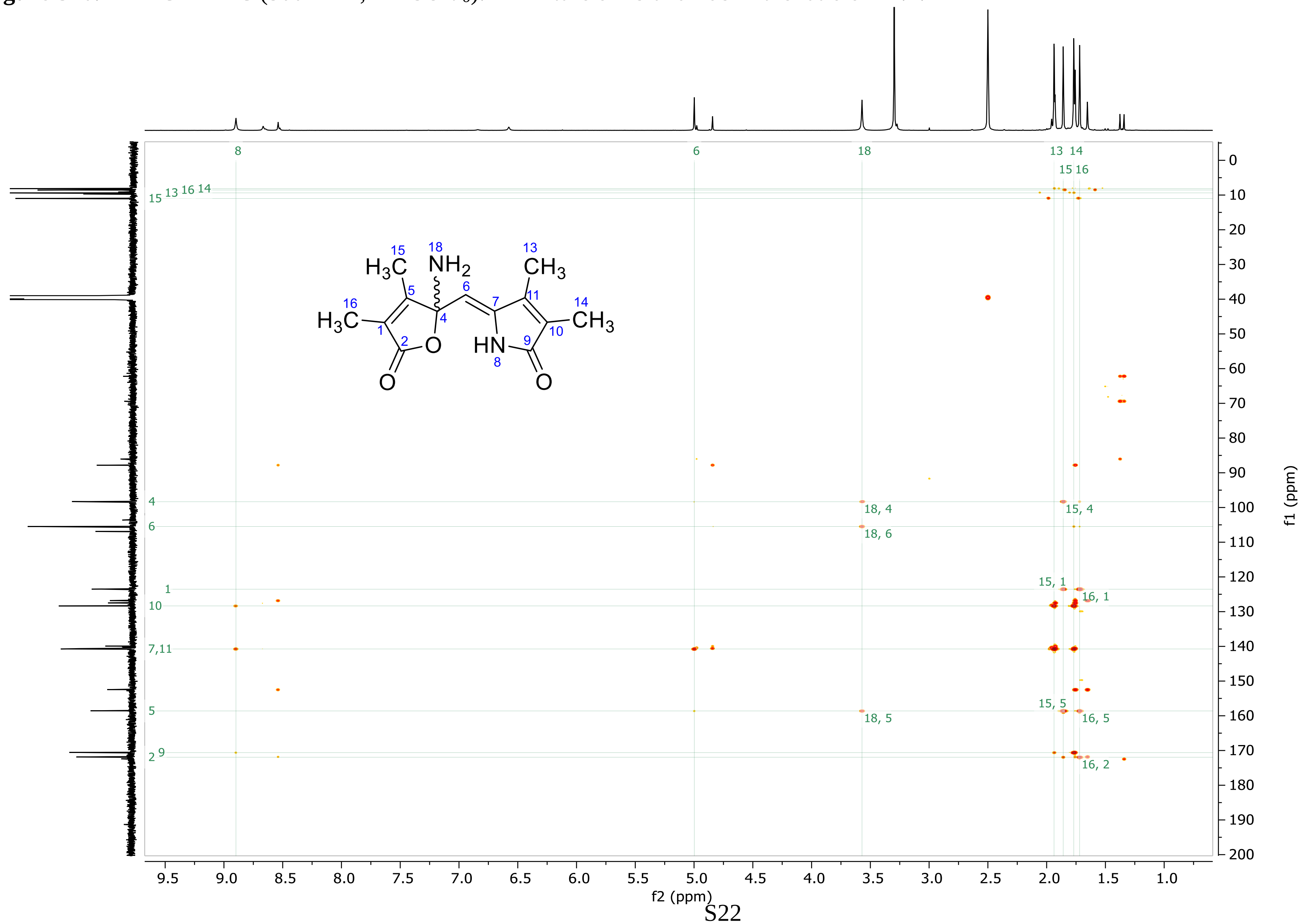

Figure S21. ^{1}H NMR (300 MHz, CF_3CO_2D): **17H⁺**.

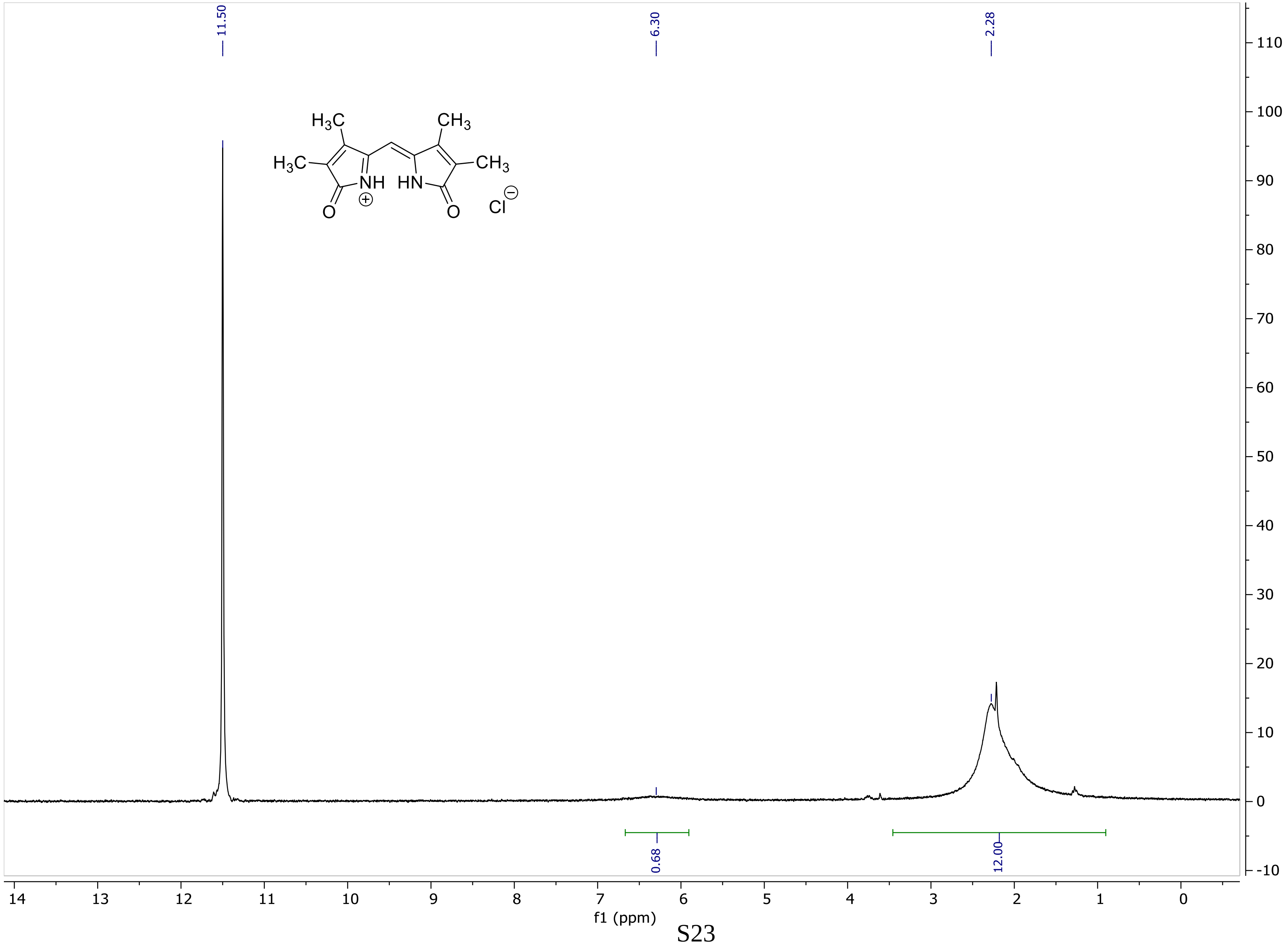

Figure S22. ^{13}C NMR (300 MHz, 0.5 mL $CDCl_3$ + 10μL CF_3CO_2H): **17H⁺**.

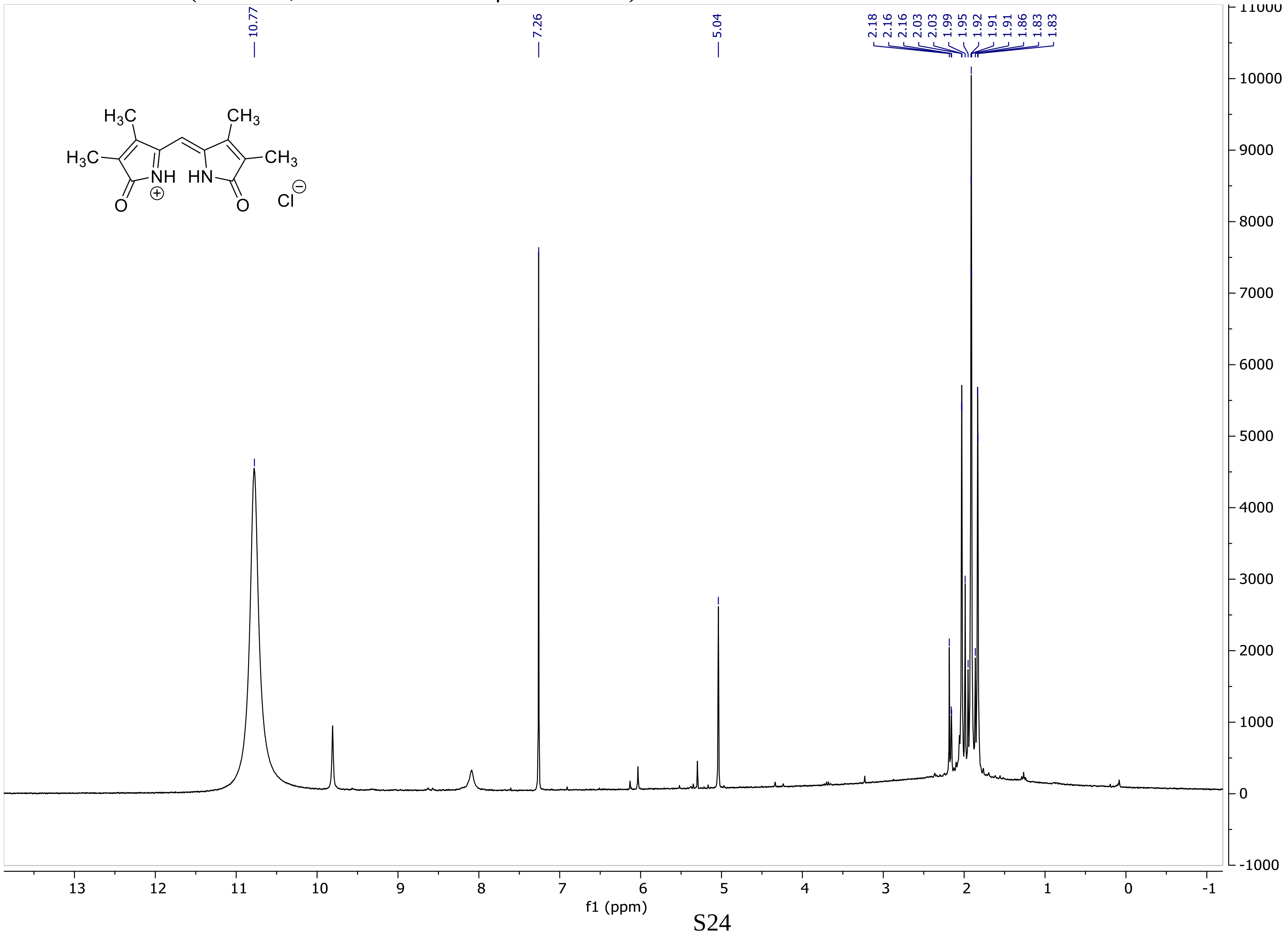

Figure S23. ^{1}H NMR (500 MHz, CD_2Cl_2): **17**.

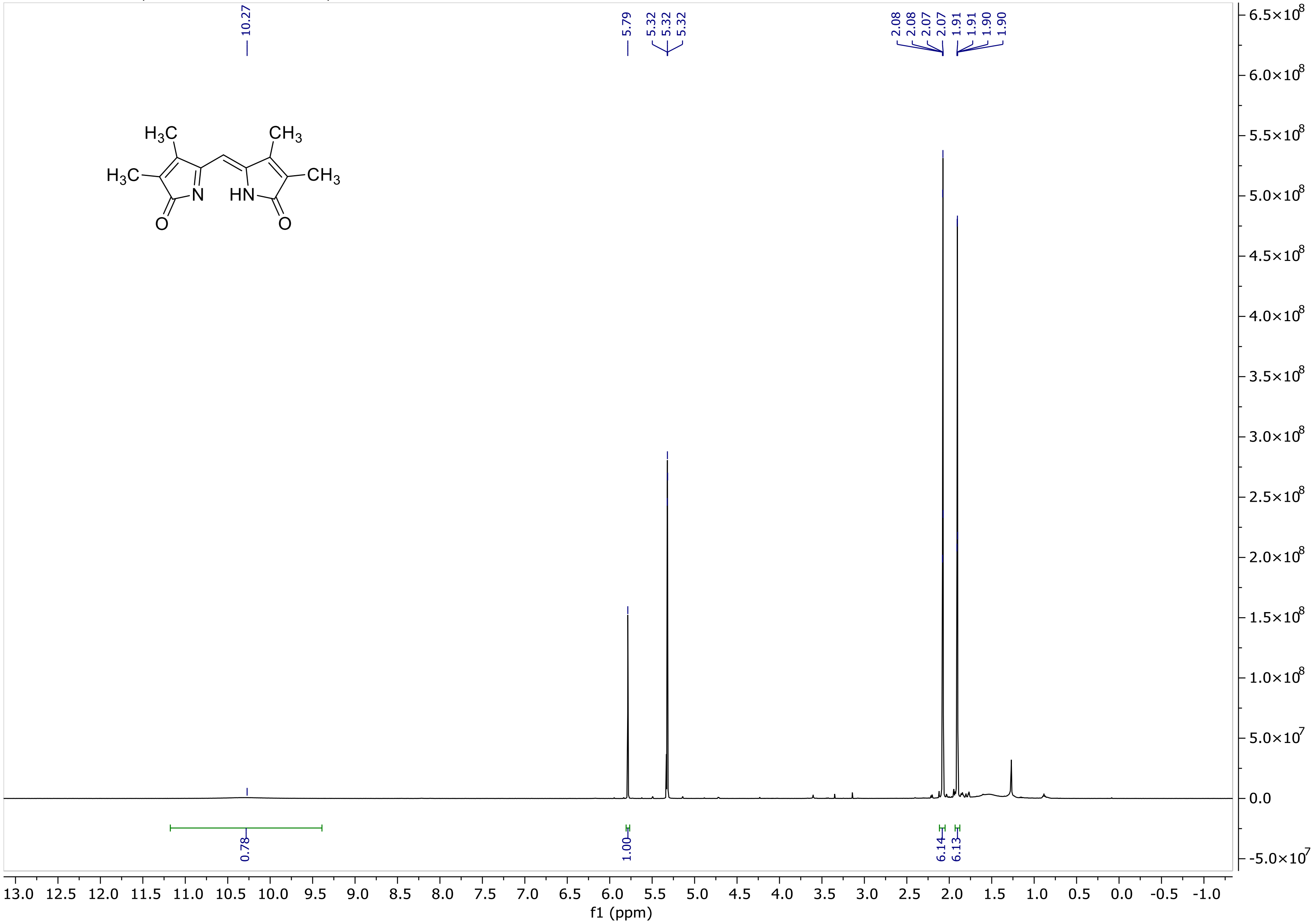

Figure S24. $^{13}C\{^1H\}$ NMR (126 MHz, CD_2Cl_2): **17**.

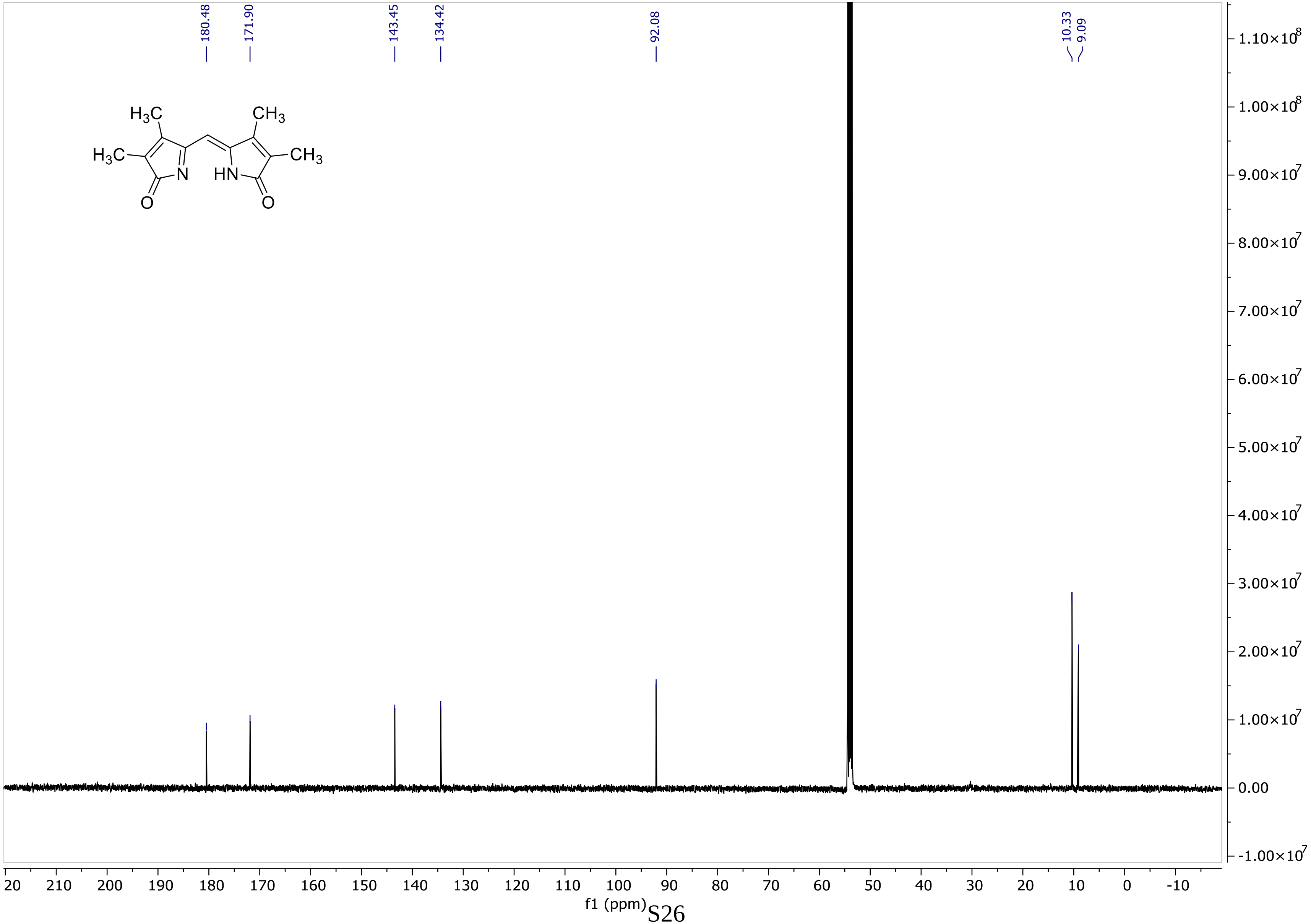

Figure S25. ^{1}H NMR (300 MHz, $CDCl_3$): 1,4-Dimethyl-1,4-dihydro-1,4-epidioxynaphthalene (**DMNO₂**).

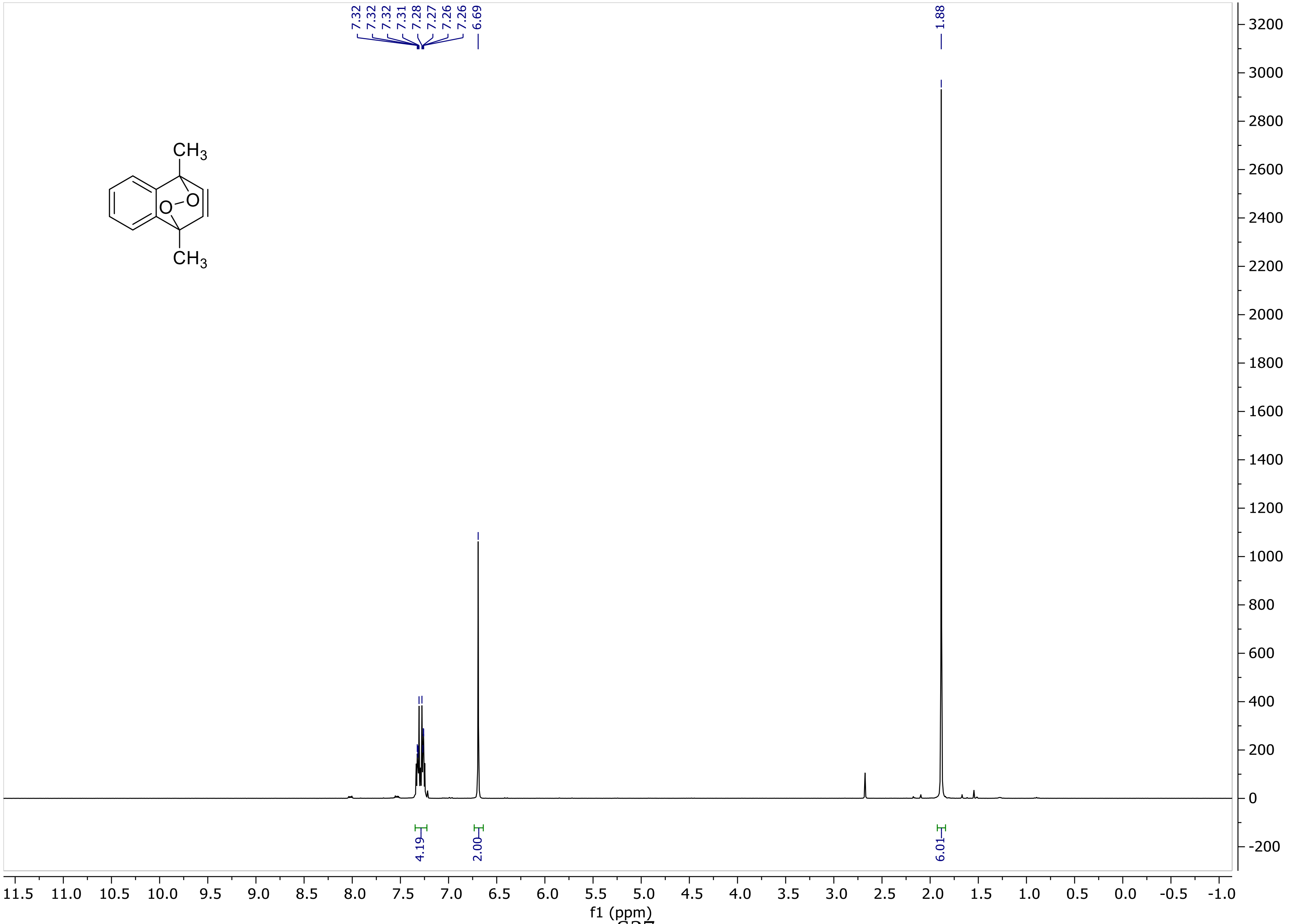

Figure S26. $^{13}C\{^{1}H\}$ NMR (75 MHz, $CDCl_3$): 1,4-Dimethyl-1,4-dihydro-1,4-epidioxynaphthalene (**DMNO$_2$**).

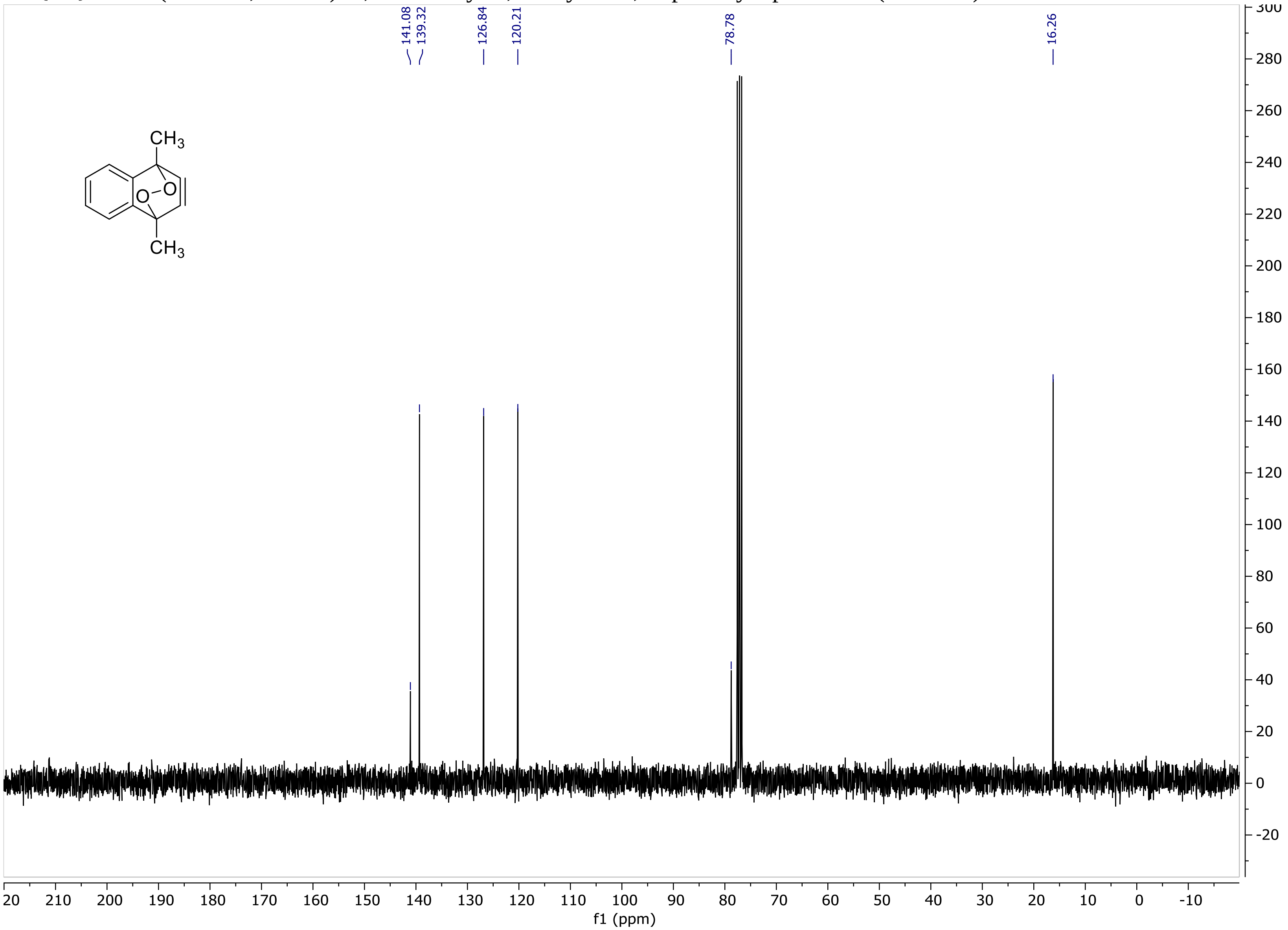

Figure S27. (a) Gated emission spectra of *Z*-**13** (c = 350 μmol L^{-1}) in frozen methanol solution at 77 K at different delay times upon excitation at 400 nm. A 450 nm long pass filter was used. The increase of intensity at longer wavelengths (>700 nm) is caused by the instrument. (b) The comparison of an instrument response function (at 400 nm) and emission trace at 480 nm upon excitation of a frozen solution of *Z*-**13**.

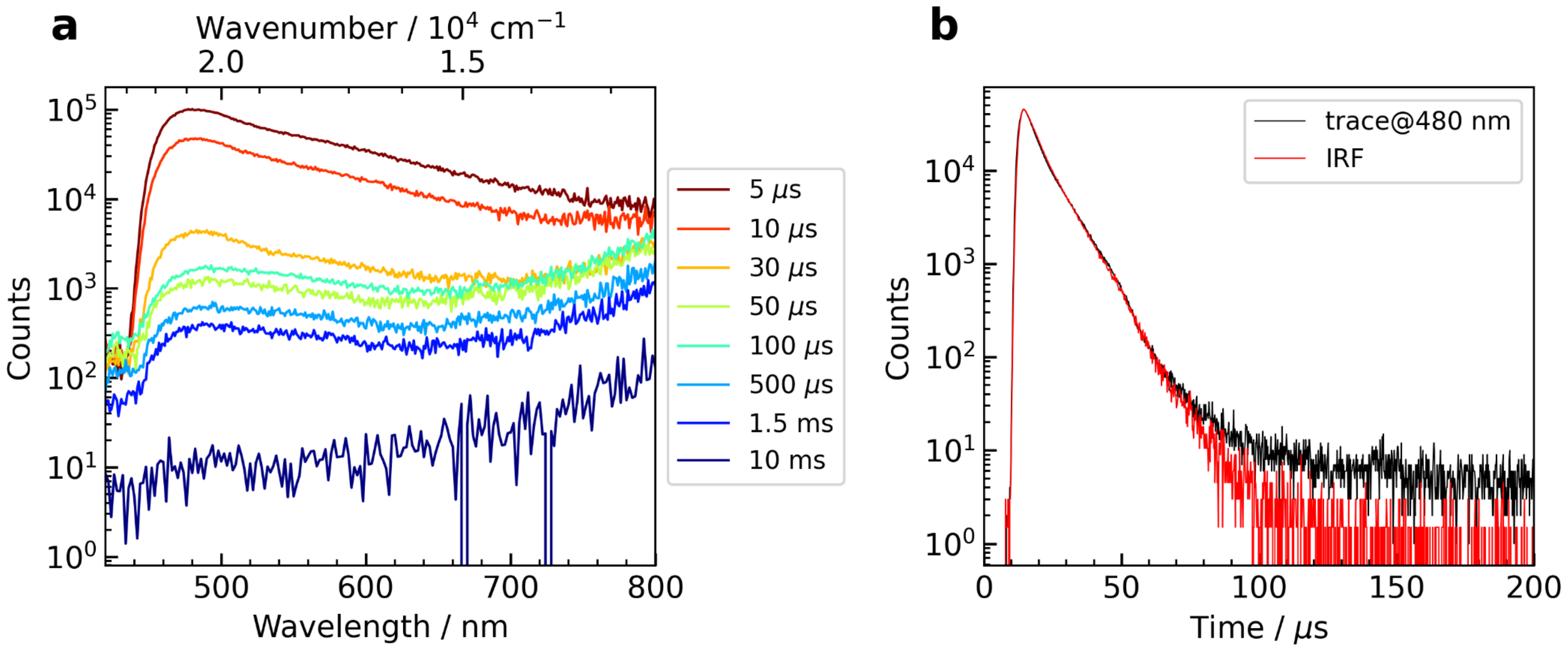

Figure S28. (a) Time-resolved absorption spectra of solvolysis of imine **17** in air-saturated methanol at room temperature in the dark. (b) Recorded (open circles) and fitted traces (solid lines) recorded at different wavelengths. The lifetime of **17** was determined to be 5.6 min by global analysis (two species, single exponential).

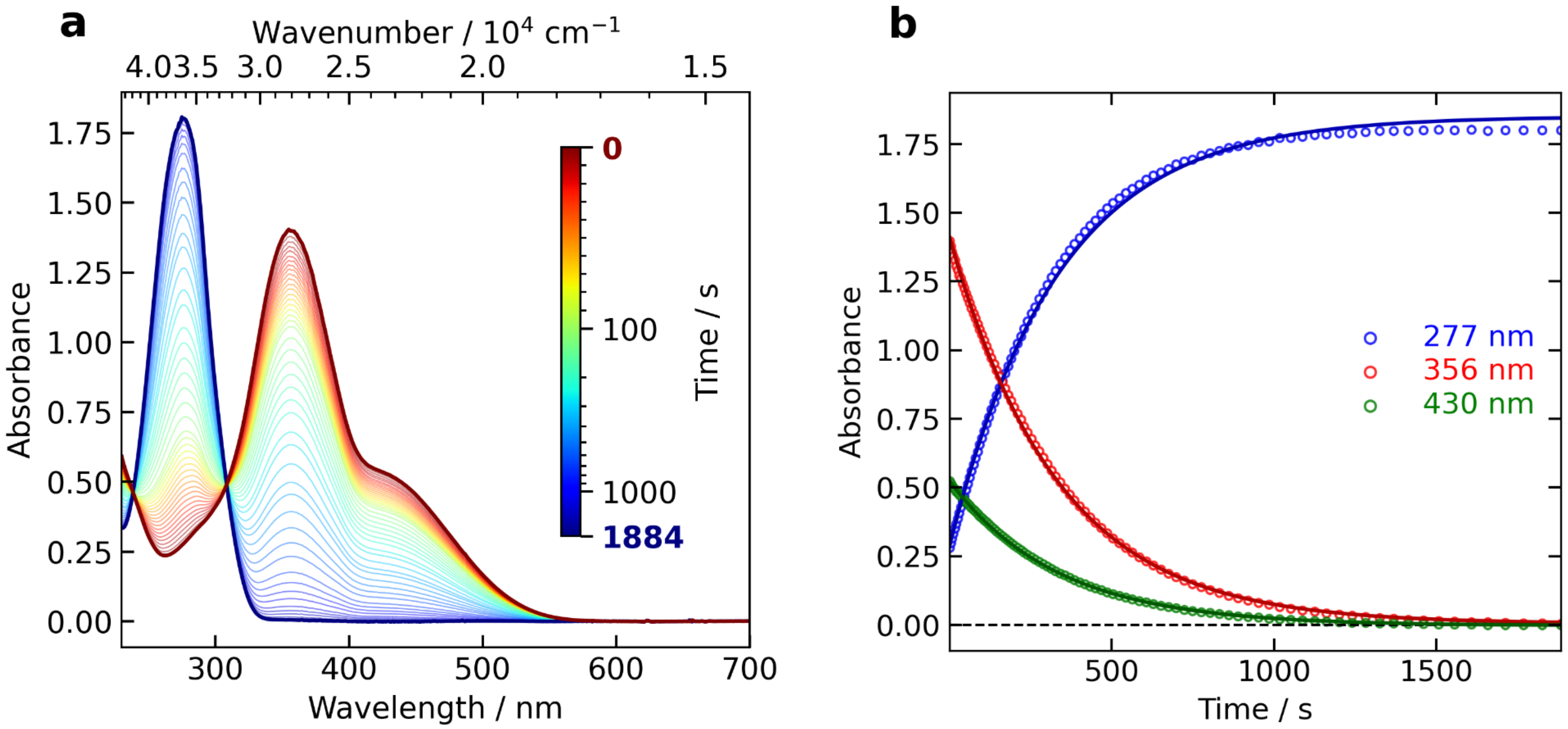

Figure S29. Time-resolved spectra from irradiation of *Z*-**13** with 409 nm LED in Ar-purged (a) toluene and (b) 10% toluene in hexane (v/v).

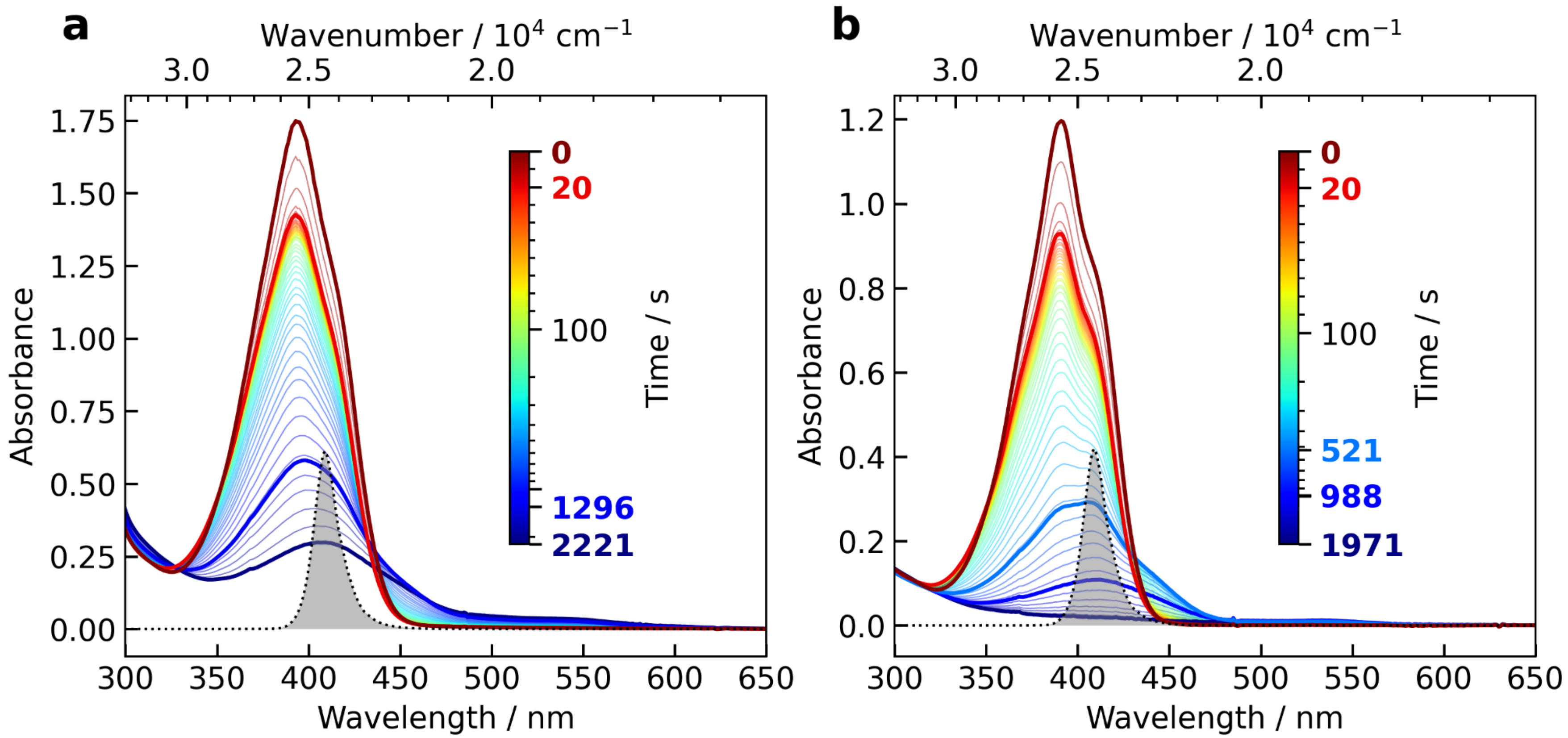

Figure S30. (a) The concentration dependence of *Z*-**13** in toluene solution. (b) Normalized absorption spectra shown on the left.

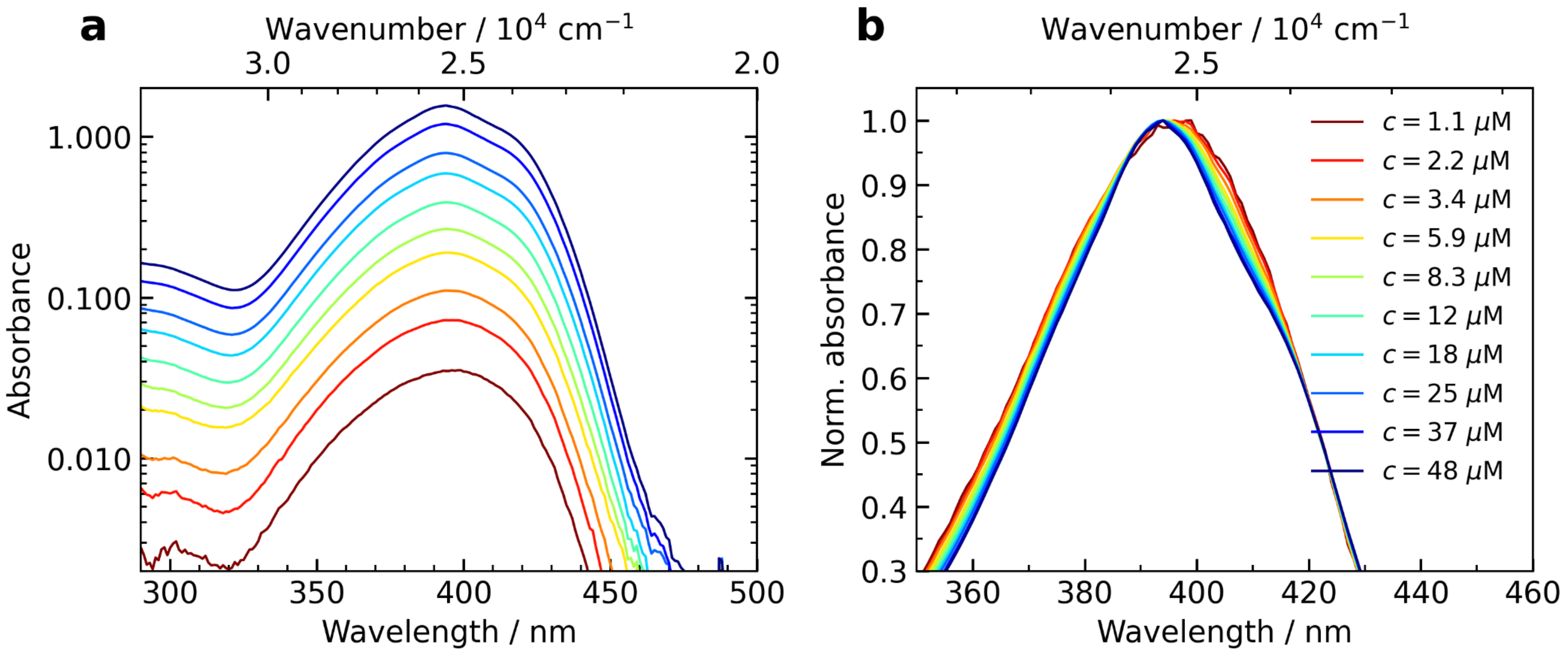

Figure S31. Irradiation of *Z*-**13** (c ~ 80 μmol L^{-1}) in toluene under degassed condition. (a) HPLC traces and extracted (b) absorption and (c) negative ESI-MS spectra from HPLC-MS data. Apart from isomerization, the formation of unknown products **S1**, **S2**, and **S3** was observed during the photoreaction. (d) Possible structures of unknown products **S1**, **S2**, and **S3** displayed in an anionic form.

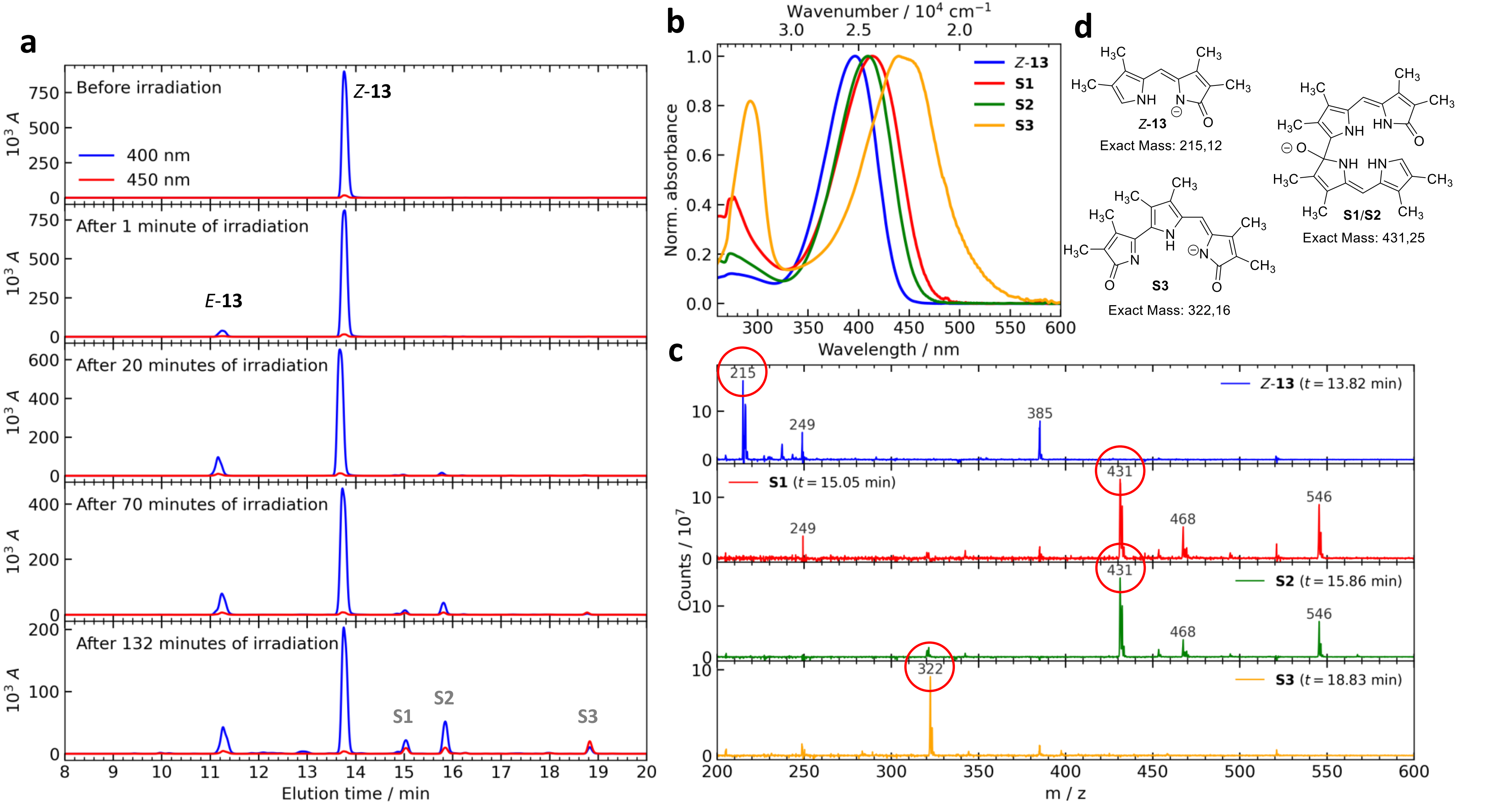

Figure S32. Overview of the photooxidation products obtained after treatment of *Z*-**13** (c = 520 μmol L^{-1}) in methanol with **DMNO₂** (c = 2.7 mmol L^{-1}, 72 hours in dark at room temperature). HPLC chromatograms developed in (a) neutral (water and methanol) and (b) acidic mobile phase (0.05% aqueous CF_3CO_2H and methanol). Unknown products are **S5b**, **S5a**, and **S4a**. Notice that amine **19** was converted to **16b** when eluted in an acidic mobile phase. 1,4-Dimethylnaphthalene is thermally produced from **DMNO₂**.

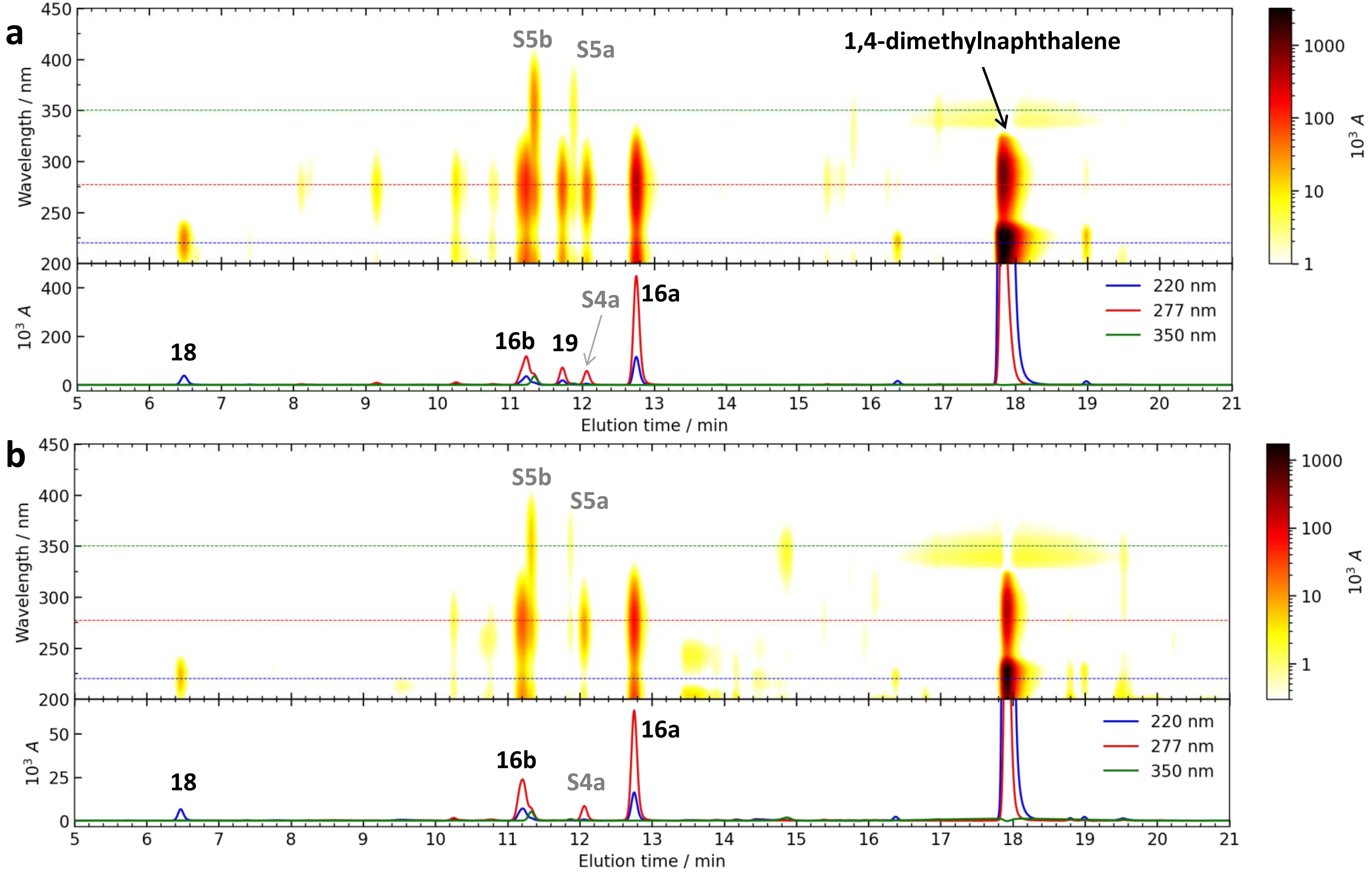

Figure S33. Overview of the photooxidation products after singlet-oxygen sensitization (535 nm LED) of *Z*-**13** (c = 200 µmol L^{-1}) with rose bengal (c = 1.2 µmol L^{-1}) for 4 min in dry acetonitrile under an oxygen atmosphere. HPLC chromatograms developed in (a) neutral (water and methanol) and (b) acidic mobile phase (0.05% aqueous CF_3CO_2H and methanol). Unknown products are **S4a**, **S4b**, **S5b**, and **S7**. Notice that amine **19** was converted to **16b** when eluted in an acidic mobile phase. Propentdyopent **16a** is formed by the reaction of imine **17** with the mobile phase during elution.

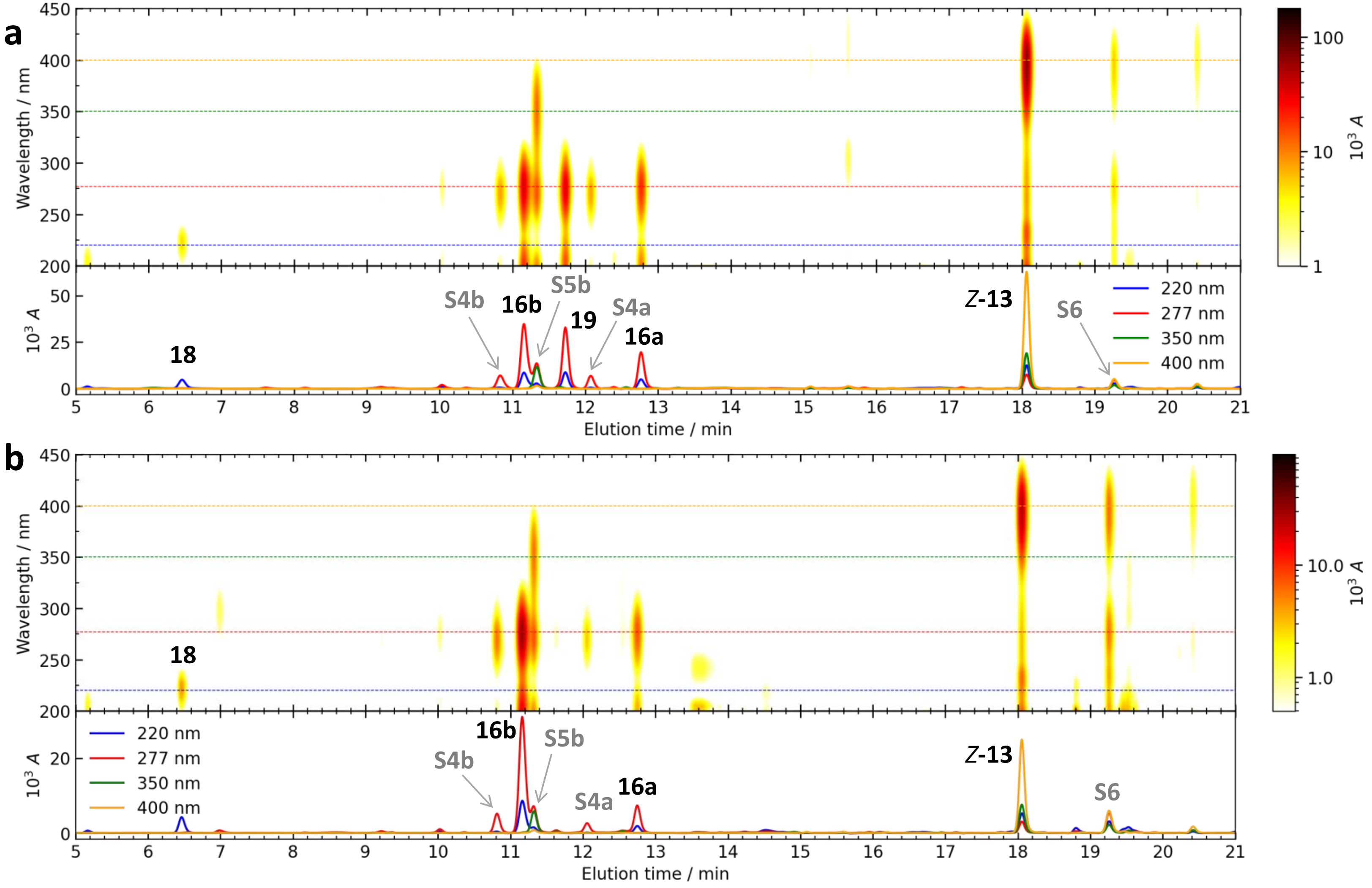

Figure S34. Overview of the photooxidation products after singlet-oxygen sensitization (535 nm LED) of *Z*-**13** (c = 190 µmol L^{-1}) with rose bengal (c = 3.6 µmol L^{-1}) for 5 min in dry acetonitrile under oxygen atmosphere. Chromatograms were recorded from the reaction mixtures obtained after 2 h incubation of the photolysate (1.0 mL) at room temperature with (a) methanol (0.5 mL) or (b) distilled water (0.5 mL). Chromatograms were developed in the neutral (water and methanol) mobile phase. Unknown products are **S4a**, **S4b**, **S5b**, and **S7**.

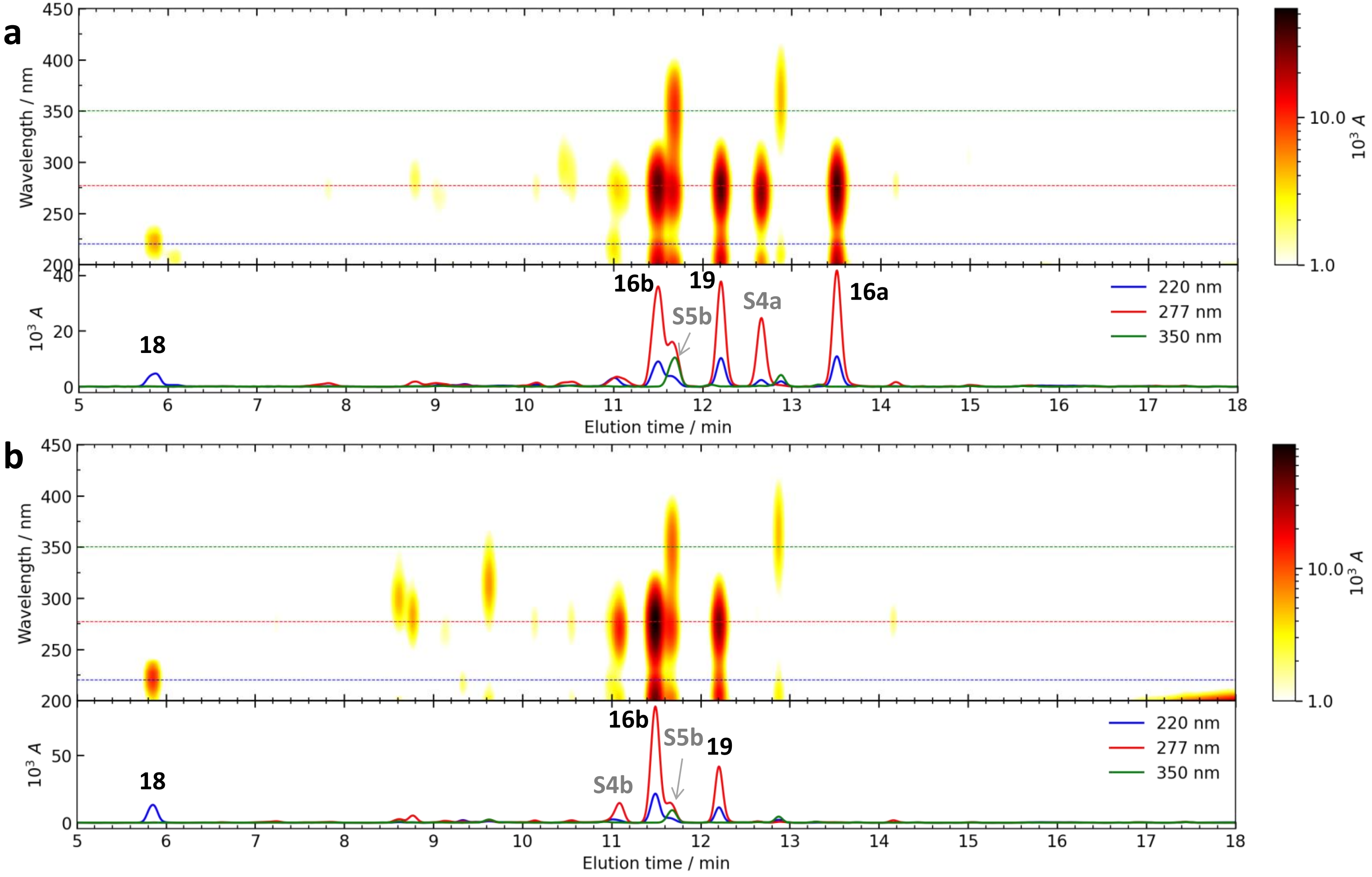

Figure S35. HPLC chromatogram of purified amine **19**. The sample also contained propentdyopent **16b** and unknown product **S4b.** See NMR spectra in Figures S15–S20.

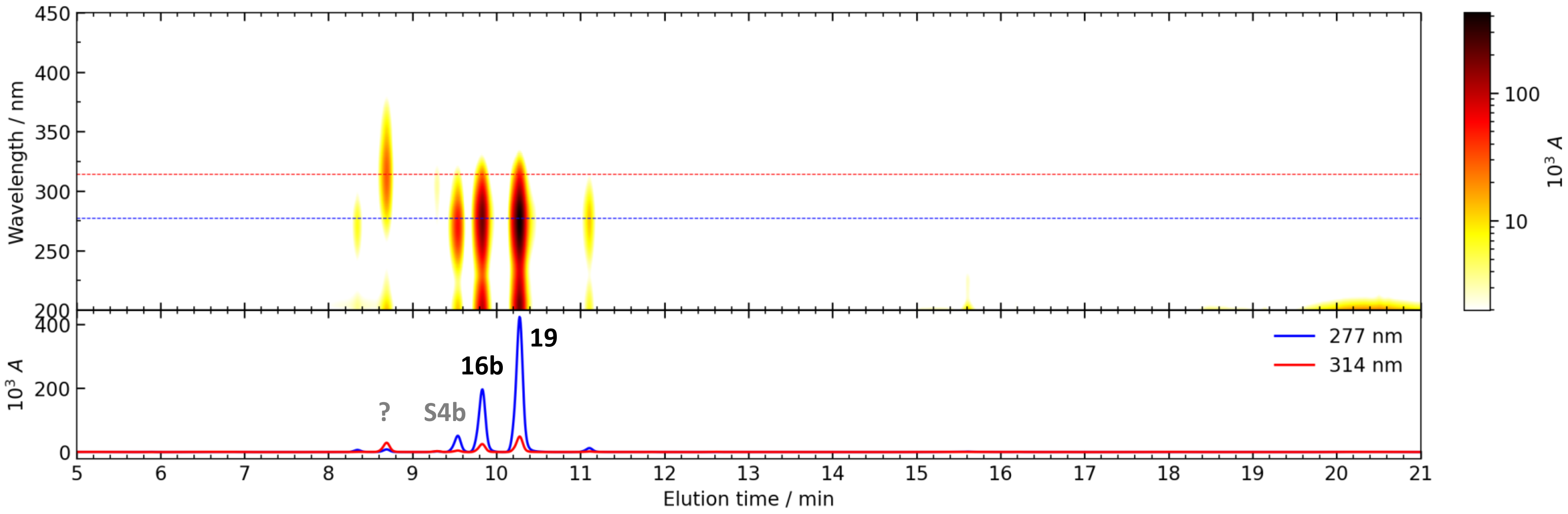

Figure S36. HPLC kinetics of direct irradiation of *Z*-**13** under air in methanol at 409 nm. Relative populations of all observed species were calculated based on integrated areas of corresponding peaks (isomers **13** and **S6** at 396 nm, **16ab**, **19**, **S4ab** at 277 nm, **18** at 222 nm and **14** at 303 nm, molar absorption coefficients were not considered). (a) Full and (b) enlarged figure. Products **S4b**, **S5a**, **S5b** and **S6** were not detected.

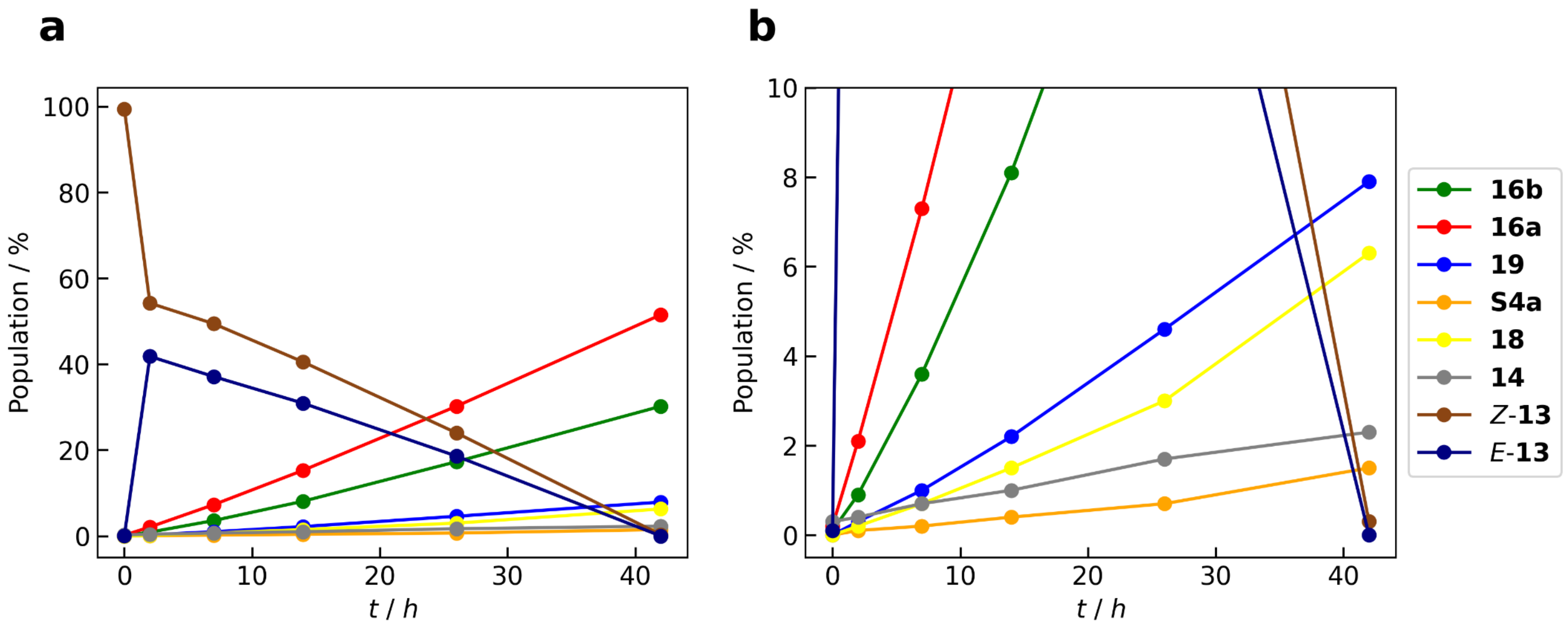

Figure S37. HPLC kinetics of direct irradiation of *Z*-**13** under oxygen in methanol at 443 nm. Relative populations of all observed species calculated based on integrated areas of corresponding peaks (isomers **13** and **S6** at 396 nm, **16ab**, **19**, **S4ab** at 277 nm, **18** at 222 nm and **14** at 303 nm, molar absorption coefficients were not considered). (a) Full and (b) enlarged figure. Products **S4b** and **14** were not detected.

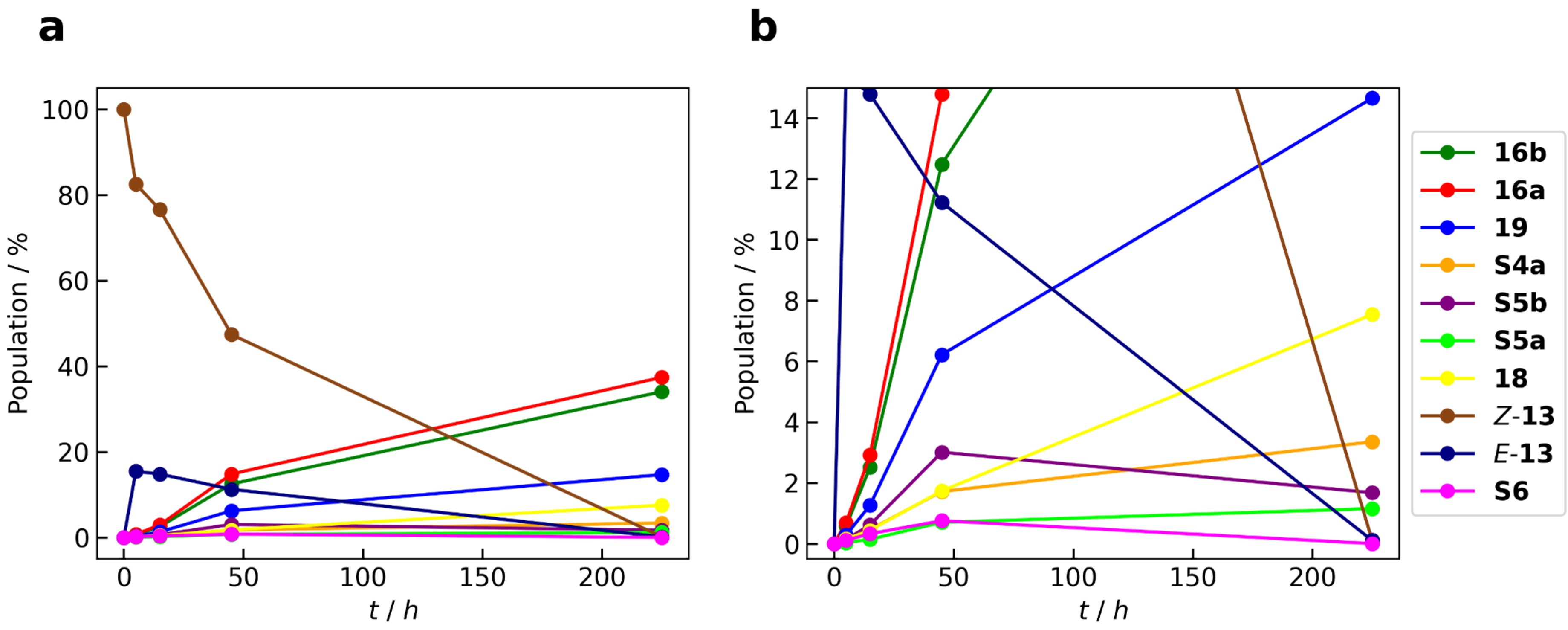

Table S2. Extension of Table 2 in the main text with unidentified photoproducts **S4a**, **S4b**, **S5a** and **S5b**.

entry	solvent	T / °C	conditions [b]	**S4a** [c]	**S4b** [c]	**S5a** [c]	**S5b** [c]
1	CH_3OH [e]	22	direct irr. (409 nm)	1.7	-	-	-
2	CH_3OH [f]	22	direct irr. (443 nm)	4.2	-	1.7	7.4
3	CH_3OH	22	RB	8.3	-	0.8	5.1
4	CH_3OH	22	MB	6.6	-	0.4	3.4
5	CH_3OH (with TFA)	22	MB	6.3	-	0.4	-
6	CH_3OH	–78 °C	RB	9.2	-	1.2	9.5
7	CH_3OH (with $NaOCH_3$)	22	RB	-	-	-	-
8	CH_3OH (*E*-**13**)	22	MB	7.8	-	1.1	5.5
9	CH_3OH	22	$DMNO_2$	7.1	-	0.7	4.3
10a [g]	CH_3CN [h]	22	RB	9.9	-	-	-
10b [g]	CH_3CN [i]	22	RB	-	8.6	-	-

[a] *Z*-**13** was used as the starting material in all experiments except entry 8, where *E*-**13** was used. [b] Direct irr. (λ/nm) is direct irradiation at a specified wavelength; in other cases, sensitizers, such as rose bengal (RB) or methylene blue (MB), or a singlet oxygen generator 1,4-dimethylnaphthalene endoperoxide ($DMNO_2$) were used. [c] Chemical yields estimated from the HPLC data (UV-vis detector; molar absorption coefficients were not considered). [e] An aerated sample was irradiated at 409 nm for 42 h at room temperature (Figure S36). [f] An oxygenated sample was irradiated at 443 nm for 225 min at room temperature (Figure S37). [g] These entries relate to a single experiment conducted with two distinct workup procedures. [h] After the reaction was completed, methanol was added, and the solution was incubated for 2 hours at room temperature to convert the imine **17** to **16a**. The yield of **16a** was then used to calculate the yield of **17**. [i] Same as in the case *h*, but excess water was added, and the yield of **17** was calculated based on the yield of **16b** (entries 10a, b; HPLC chromatograms are shown in Figure S34).

Figure S38. ESI-MS spectra of known and unknown compounds extracted from HPLC-MS data from oxidation experiments in (a) a negative and (b) positive mode. See the next page for the extracted absorption spectra.

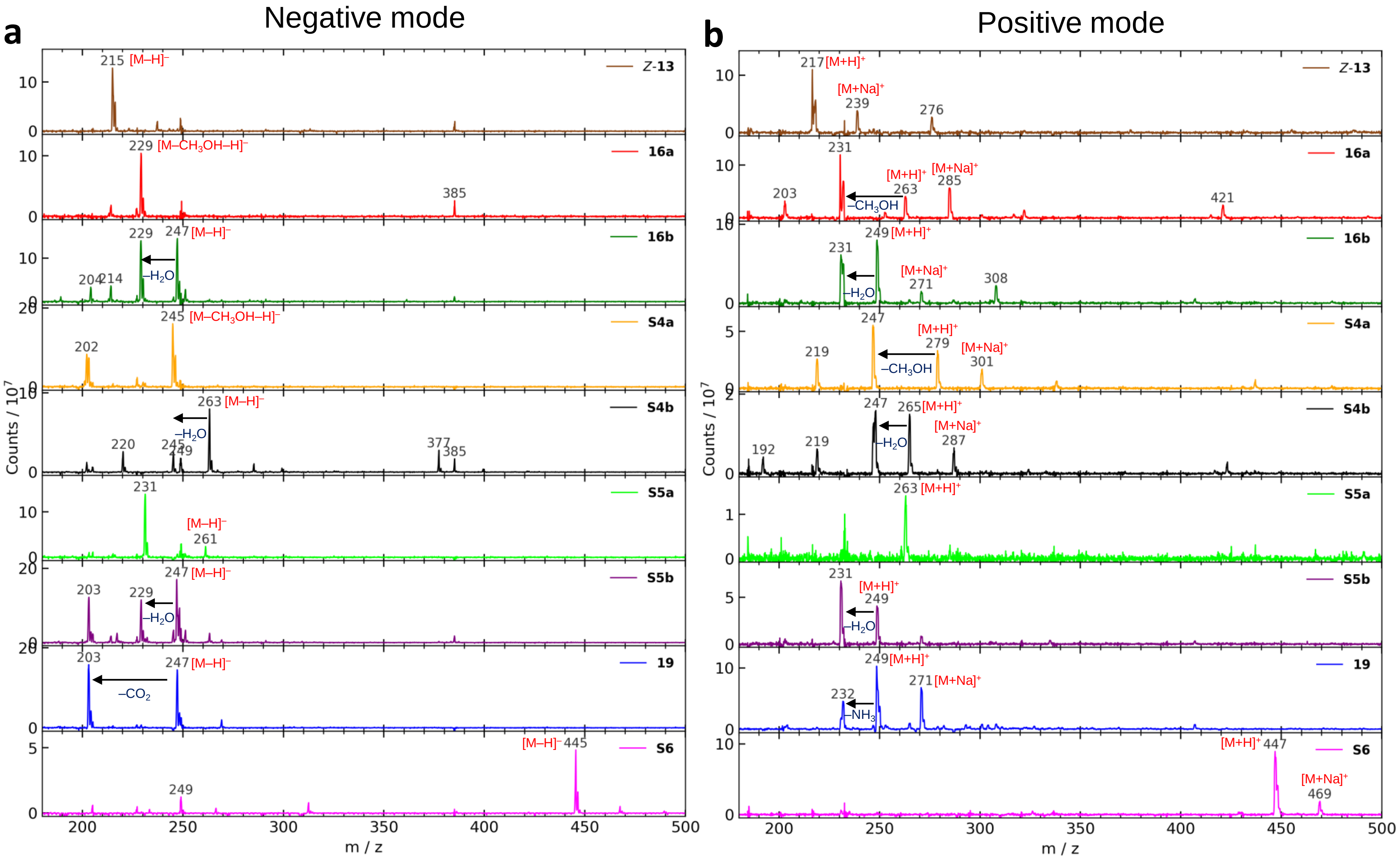

Figure S39. (a) Proposed structures and masses for unknown compounds along with the observed behavior and fragments under MS conditions. (b) Extracted absorption spectra from HPLC data for all species observed during the oxidation experiments.

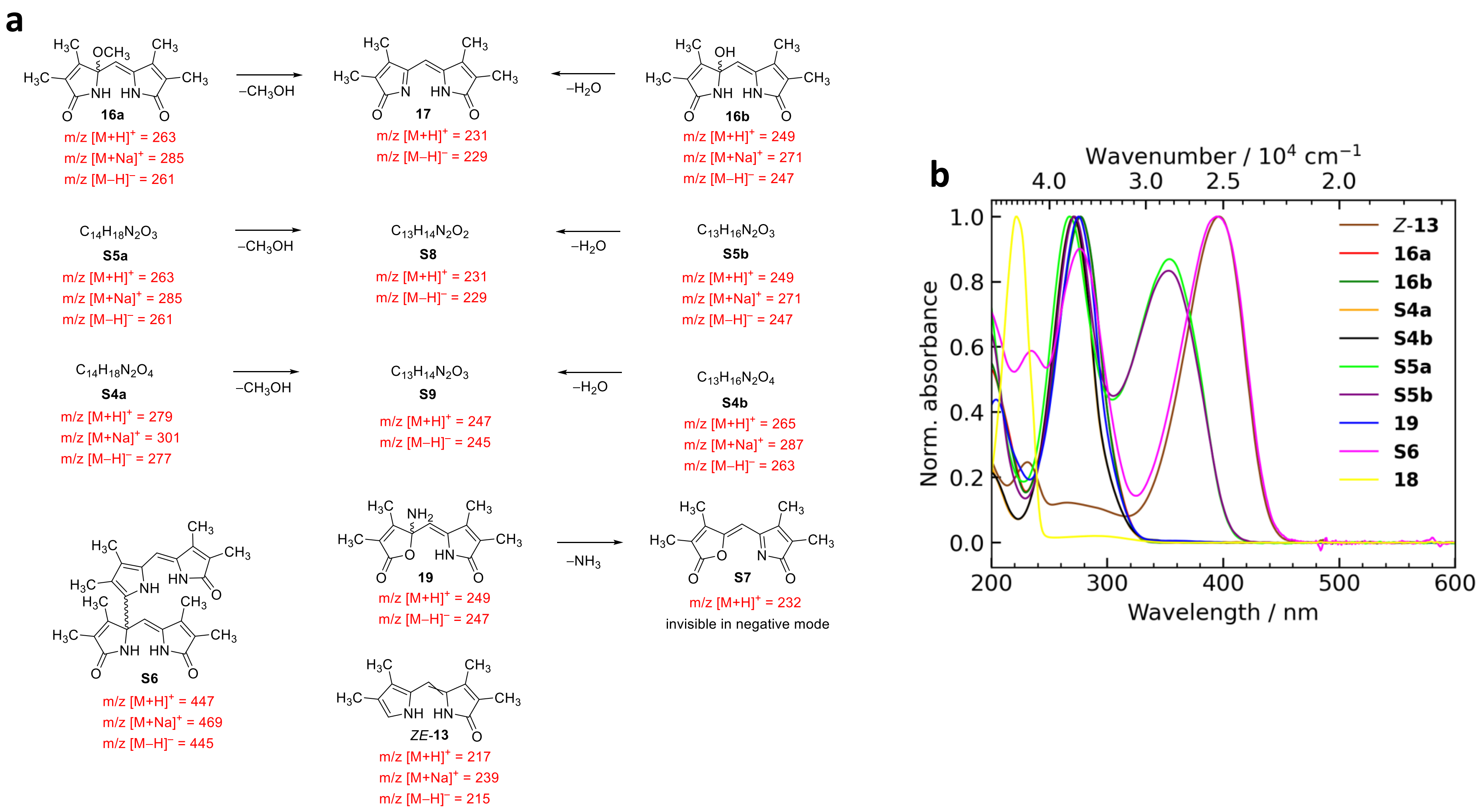

Supplementary Commentary on Products of *Z*-13 Formed by Oxidation with Singlet-Oxygen

S6: Both positive and negative MS spectra clearly point to the adduct of *Z*-**13** and imine **17** (a possible structure displayed as **S6** in Figure S39a). Furthermore, the absorption spectrum of **S6** consists of two bands (395 and 276 nm), and the spectrum resembles the sum of individual spectra of *Z*-**13** and propentdyopent **16a**/**16b**, suggesting that the structure **S6** contains both chromophores which perform independently. We observed that **S6** is formed in a reaction mixture where both imine **17** and *Z*-**13** are present. This is mostly in acetonitrile, where **17** is stable; nevertheless, even in methanol, traces of **S6** could be observed during irradiation. We propose that adduct **S6** is formed by the nucleophilic addition of the pyrrole 2 position of *Z*-**13** to electrophilic carbon of **17**.

Amine **19:** In positive ESI-MS (Figure S38), the molecular ion $[\mathbf{19} + H]^+$ loses ammonia, and *m/z* 232 is detected. On the contrary, propentdyopents such as **16a** and **16b** always show the iminium $\mathbf{17}H^+$ as *m/z* 231. Furthermore, in negative mode, the behavior for $[\mathbf{19}–H]^–$ is different from that of propentdyopents because **S7** (Figure S39a) does not possess any easily detachable proton and, therefore, cannot be observed in negative ESI-MS. Loss of CO_2 from the molecular ion is observed instead (Figure S38).

S4a and **S4b**. The UV-vis spectra of both compounds show absorption bands at 272 nm. This suggests that the structures should be similar to propentdyopents (there are no π-extending chromophores). It is also apparent that the pair **S4a** and **S4b** is in the same relationship as that of **16a** and **16b**. The molecular peak of **S4a** ($[\mathbf{S4a} + H]^+$, *m/z* 279) loses a molecule of methanol whereas that of **S4b** ($[\mathbf{S4b} + H]^+$, *m/z* 265) loses a molecule of water, both yielding the peak *m/z* 247 ($[\mathbf{S8} + H]^+$, Figure S38, Figure S39a). However, **S4a** and **S4b** contain an extra atom of oxygen compared to propentdyopents **16a** and **16b**. In negative mode, the behavior is very similar to that of propentdyopents **16a** and **16b**. Similar to **16a**, the molecular peak for **S4a** is not observed; only the peak formed upon the loss of a molecule of methanol (*m/z* 245, $[\mathbf{S8} – H]^–$, see Figure S38a) is visible. On the other hand, **S4b** shows a molecular peak in negative mode, similar to **16b**. As expected, because **S4a** must contain the methoxy group, we observed product **S4a** only when the reaction was conducted in methanol. Interestingly, we also observed the formation of **S4a** by methanolysis of the photolysate resulting from irradiation of *Z*-**13** in acetonitrile. Similarly, we observed increased production of **S4b** upon hydrolysis of the same photolysate (see Table S2). It suggests that some intermediate, possibly **S9**, is present in the reaction mixture and then selectively reacts with methanol or water to produce **S4a** or **S4b**. Here, we propose structures that would be consistent with MS and UV-vis data. An extra oxygen is either present as epoxide (**S4ab-I,II,III**), or rearrangement to give diketone (**S4ab-IV,V**) occurred.

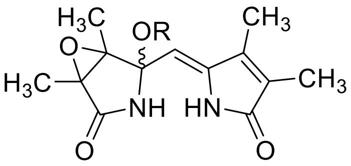

S4a-I: R = CH_3
S4b-I: R = H

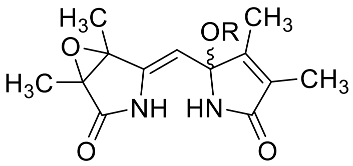

S4a-II: R = CH_3
S4b-II: R = H

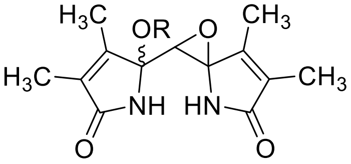

S4a-III: R = CH_3
S4b-III: R = H

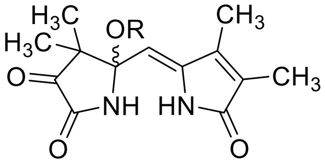

S4a-IV: R = CH_3
S4b-IV: R = H

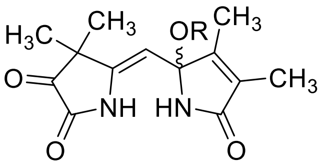

S4a-V: R = CH_3
S4b-V: R = H

S5a and **S5b**: The UV-vis spectra of these compounds show absorption bands at ~270 and ~353 nm. This suggests that the structures must possess a π-extended chromophore, such as that of propentdyopents **16a** and **16b** or other propentdyopent-like molecules. However, the conjugation is less extended than, for example, that in *Z*-**13**, which absorbs up to 450 nm. The MS data show that the masses of **S5a** and **S5b** are the same as those of **16a** and **16b**, respectively. Also, their behavior under MS conditions is similar. In the case of **S5a**, the signal was very weak due to a very low concentration of **S5a** in the oxidized mixtures. Here, we propose various structures. When we performed TDDFT calculations (B3LYP-6–311++G(d,p) level of theory with methanol as a solvent)[4] of some of the proposed structures, we noticed that the observed UV-Vis spectrum resembles that of the calculated spectrum of hydroperoxide intermediate **21a** (see Figure S40 and the main text). Therefore, we proposed another structure, **S5ab-II**, which possesses an equivalent chromophore. Both structures **S5ab-I** and **S5ab-II** could potentially be formed by the rearrangement of **21a** or by [2+2] attack of singlet oxygen on the pyrrole ring of *Z*-**13**, followed by rearrangement. The absorption spectra of known and unknown species follow (Figure S40).

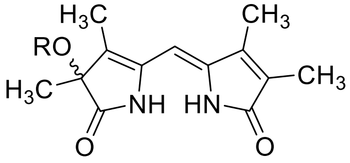

S5a-I: R = CH_3
S5b-I: R = H

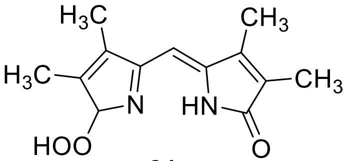

21a

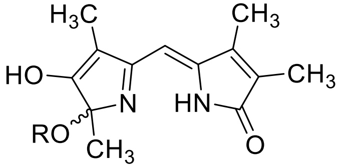

S5a-II: R = CH_3
S5b-II: R = H

Figure S40. (a) The calculated absorption spectra for different known and proposed structures (B3LYP-6–311++G(d,p) level of theory, full width at half maximum of the Gaussian functions, from which the spectra were constructed, were set to 0.25 eV). (b) Comparison of the normalized calculated (solid lines) and recorded (dashed lines) absorption spectra.

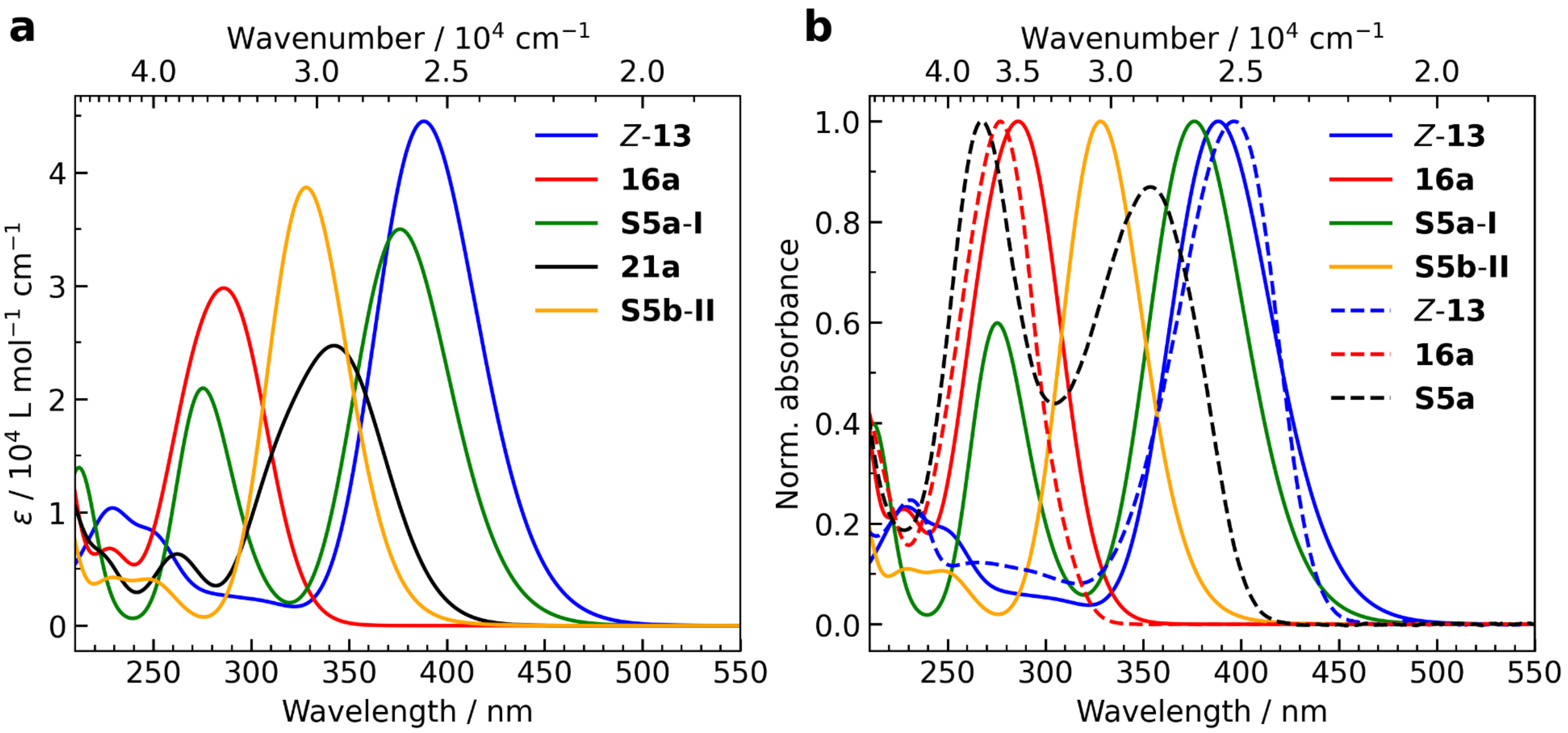

Figure S41. Sensitization of *Z*-**13** (c ~ 50 µmol L^{-1}) with RB (c ~ 7 µmol L^{-1}) under an oxygen atmosphere with 532 nm LEDs in methanol. (a) Irradiation was stopped after the formation of an intermediate absorbing at ~310 nm (8 s, orange line), and the reaction was stirred in the dark. (b) The cuvette was irradiated for the whole time.

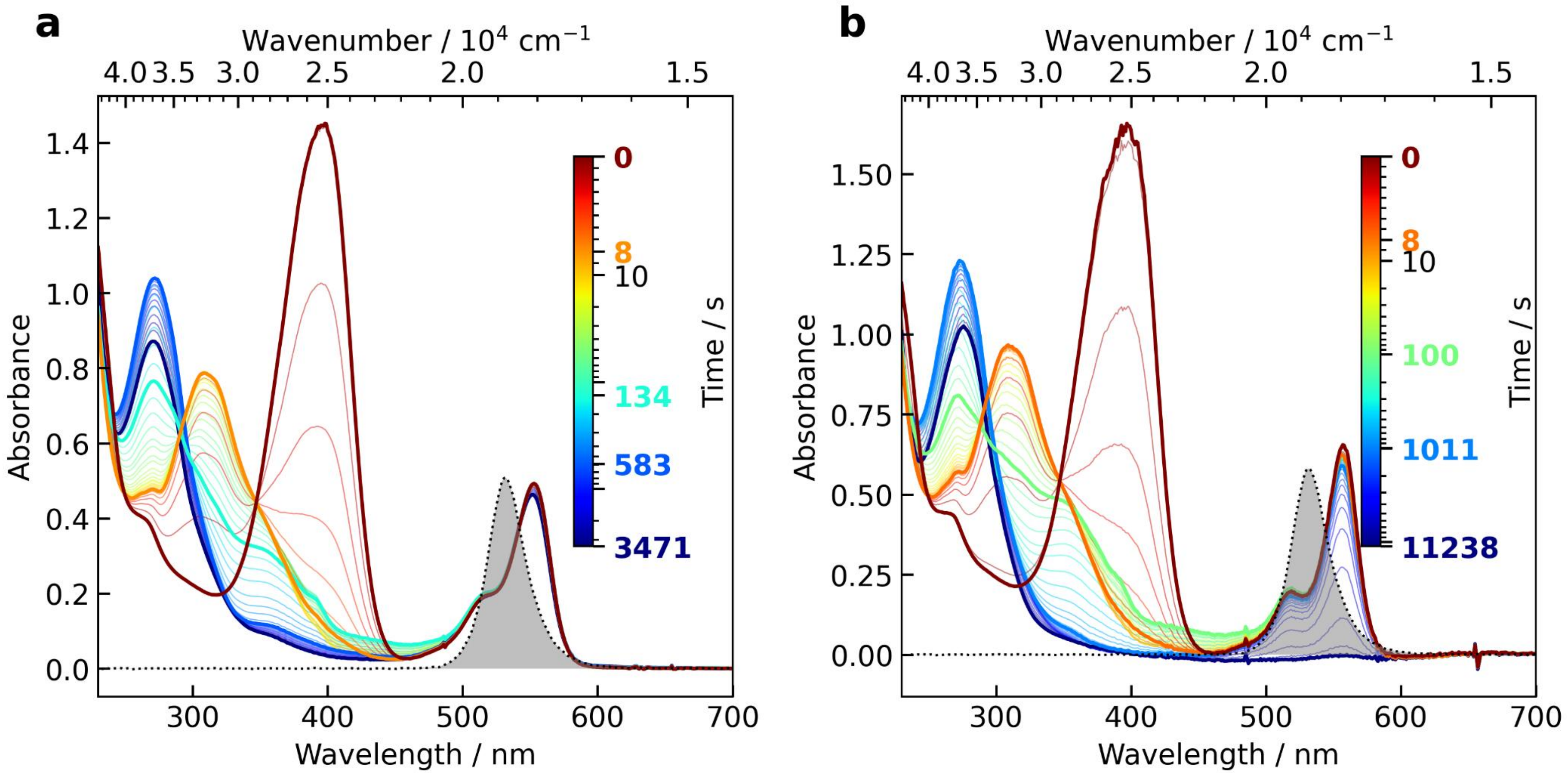

Figure S42. Sensitization of *Z*-**13** (*c* ~ 60 µmol L^{-1}) with RB (*c* ~ 6 µmol L^{-1}) under an oxygen atmosphere with 532 nm LEDs in methanol. Irradiation was stopped after the formation of an intermediate absorbing at ~310 nm (8 s, orange line), and CF_3COOH (50 µL) was added to the cuvette in one portion. Then, the cuvette was stirred in the dark.

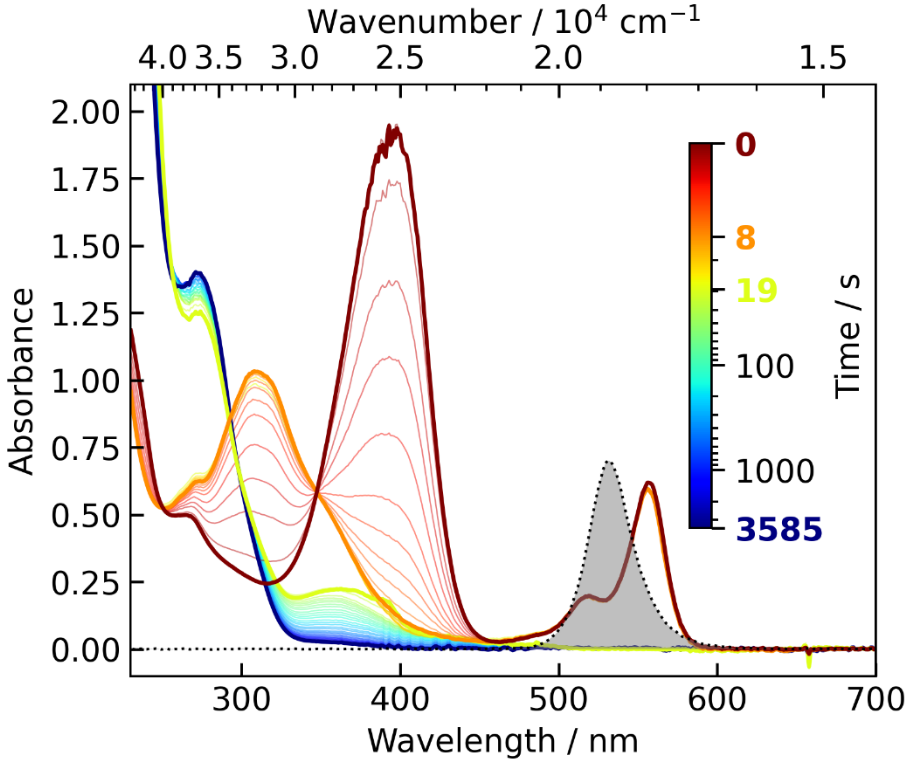

Figure S43. Sensitization of (a) **16a** (c ~ 86 µmol L^{-1}) and (b) **16b** (c ~ 100 µmol L^{-1}) with RB (c ~ 7 µmol L^{-1}) under an oxygen atmosphere with 532 nm LEDs in methanol.

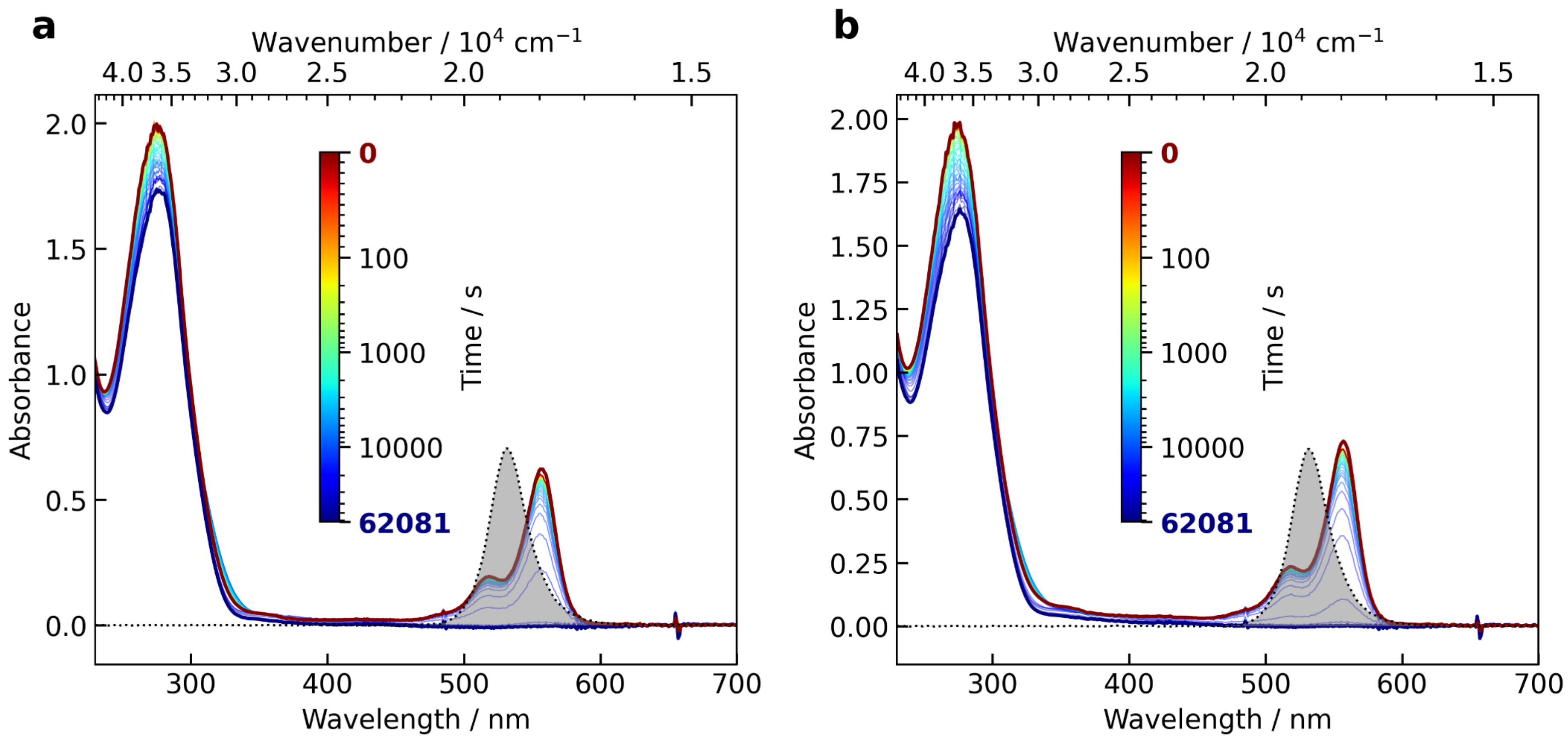

Figure S44. Reaction of *Z*-**13** (c ~ 30 µmol L^{-1}) with singlet oxygen produced thermally by **DMNO₂** (c ~ 400 µmol L^{-1}) in methanol at 30 °C. The formation of 1,4-dimethylnaphthalene is visible at ~288 nm.

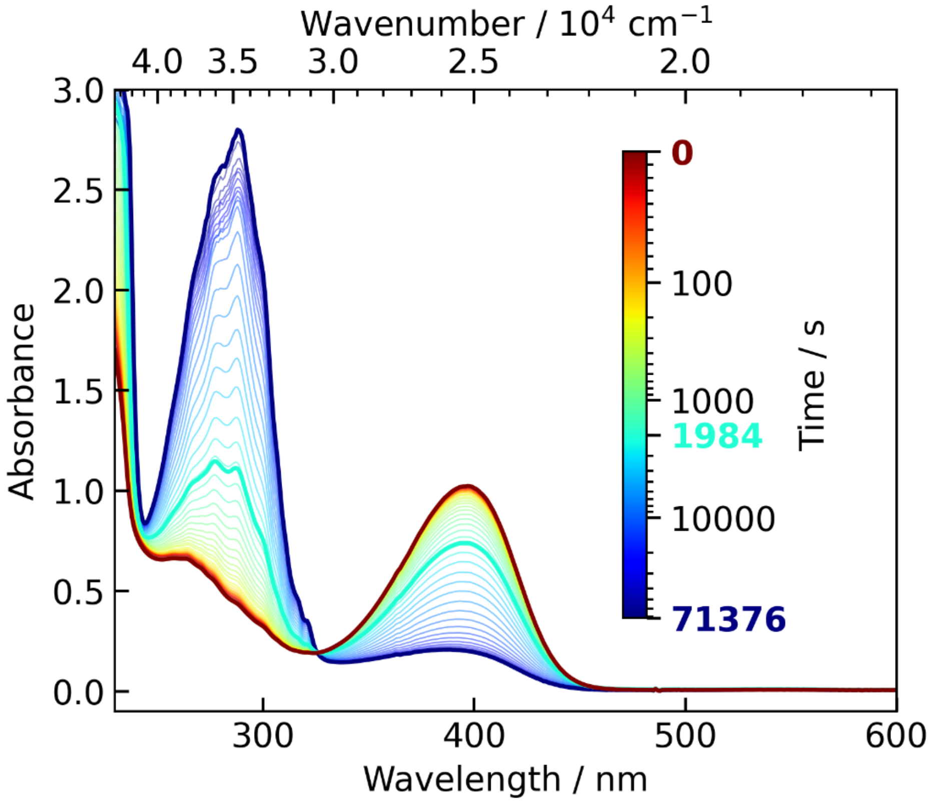

Figure S45. Sensitization of *Z*-**13** (c ~ 30 µmol L^{-1}) with RB (c ~ 5 µmol L^{-1}) in dry acetonitrile with 532 nm LEDs.

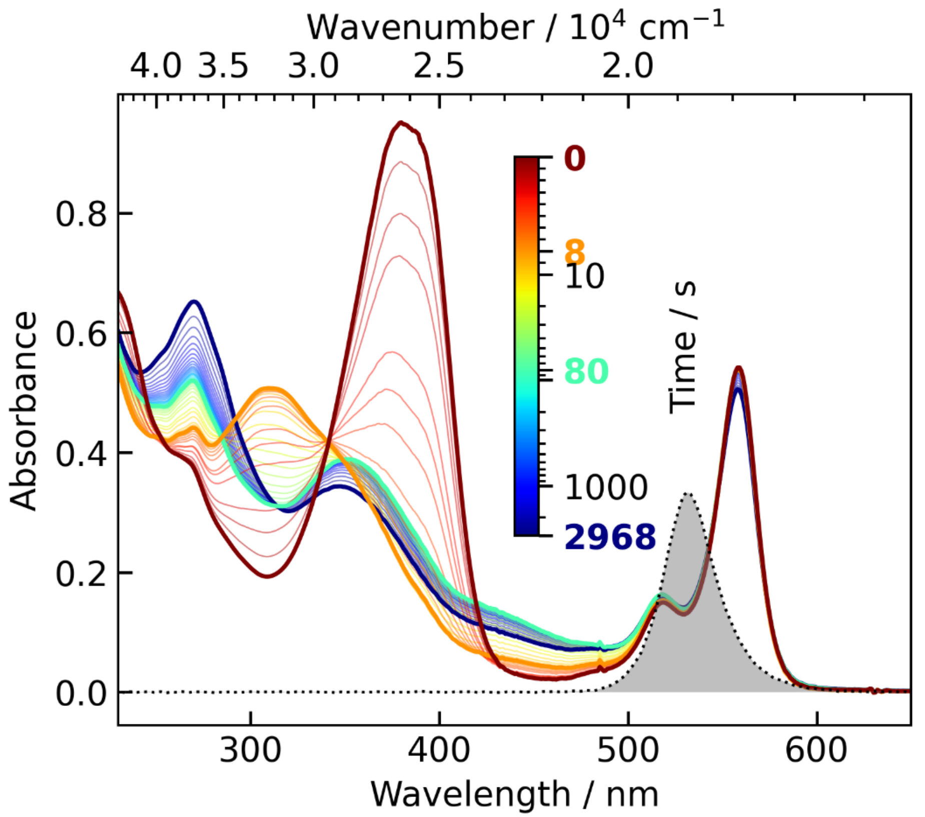

Figure S46. (a) Absorption spectra of *Z*-**13** in methanol at different concentrations (the values of *A* > 2.5 were removed). (b) Concentration-dependent absorbance at the selected wavelengths, denoted as dashed vertical lines in (a) and the corresponding molar absorption coefficients.

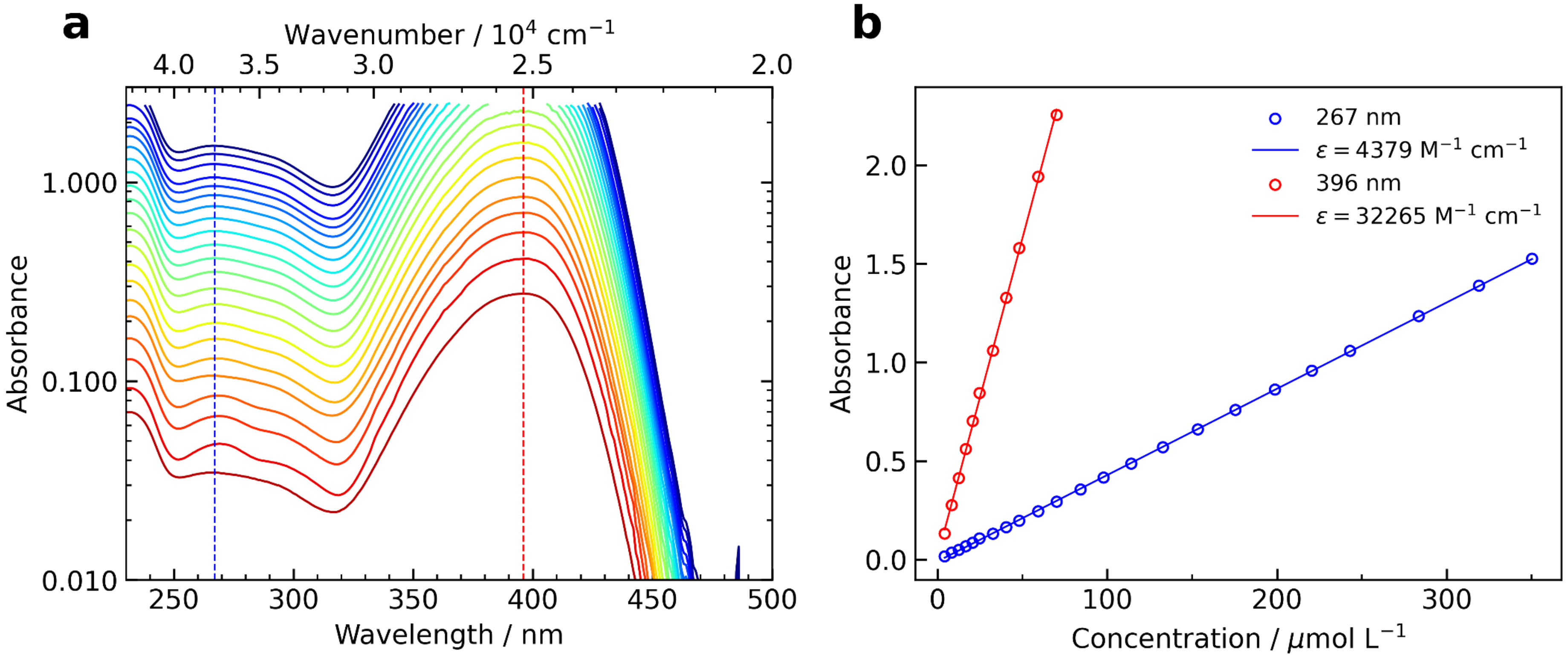

Figure S47. (a) Absorption spectra of **17** in dichloromethane at different concentrations (the values of $A > 2.5$ were removed). (b) Concentration-dependent absorbance at the selected wavelengths, denoted as dashed vertical lines in (a) and the corresponding molar absorption coefficients.

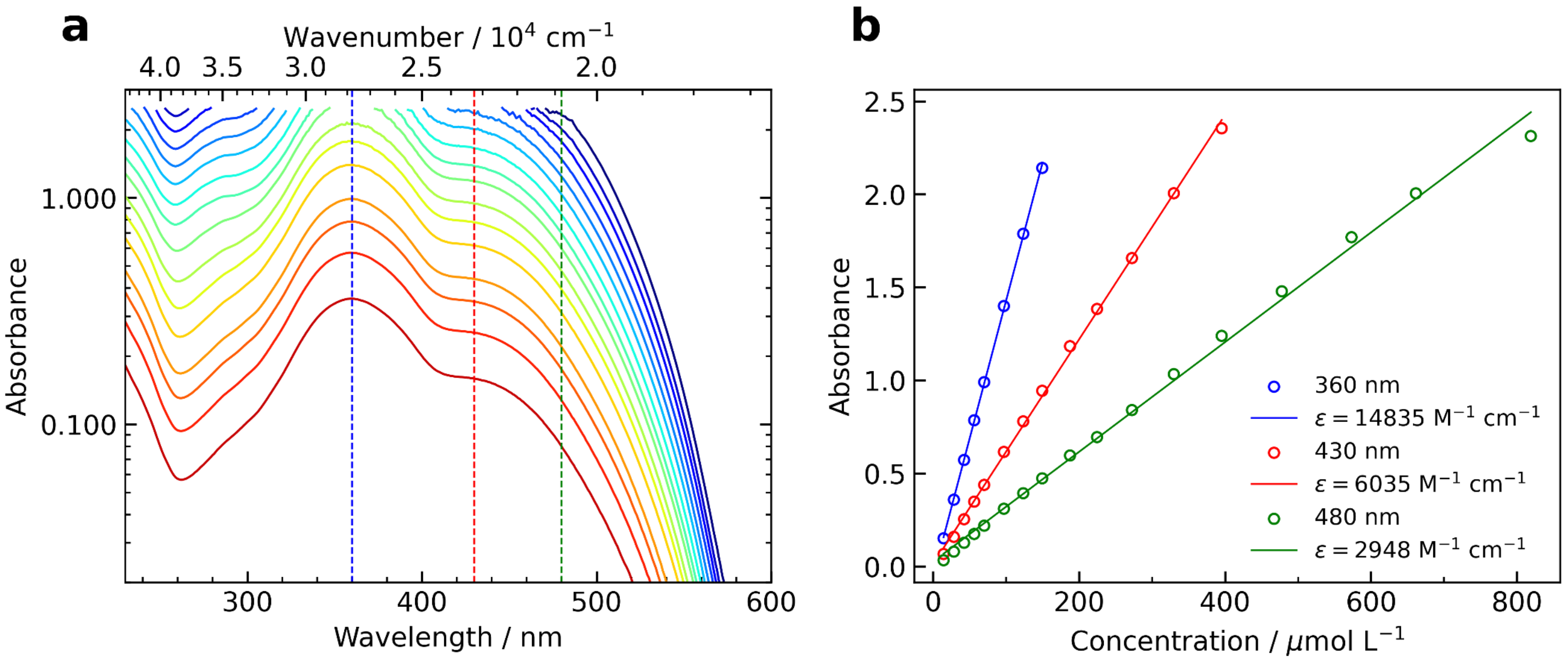

Figure S48. (a) Formation of monoformazan from NBT by addition NBT solution in methanol (100 μL, *c* ~ 100 μmol L^{-1}) to saturated solution of sodium ascorbate in methanol. Aggregation of formed species is apparent after 28 s of the reaction. Next follows difference absorbance spectra: (b) Blank experiment - thermal reaction of **DMNO₂** (*c* = 0.4 mmol L^{-1}) with NBT (*c* = 5.5 μmol L^{-1}) in methanol at 30 °C, (c) oxidation of *Z*-**13** (*c* = 50 μmol L^{-1}) with singlet oxygen by **DMNO₂** (*c* = 0.4 mmol L^{-1}) in the presence of NBT (*c* = 5.5 μmol L^{-1}) in methanol at 30 °C, (d) same experiment as (c) with same concentrations of **DMNO₂** and NBT but in the mixture of methanol (1.62 mL) and water (1.00 mL) with $c_{Z\text{-}\mathbf{13}}$ = 32 μmol L^{-1}.

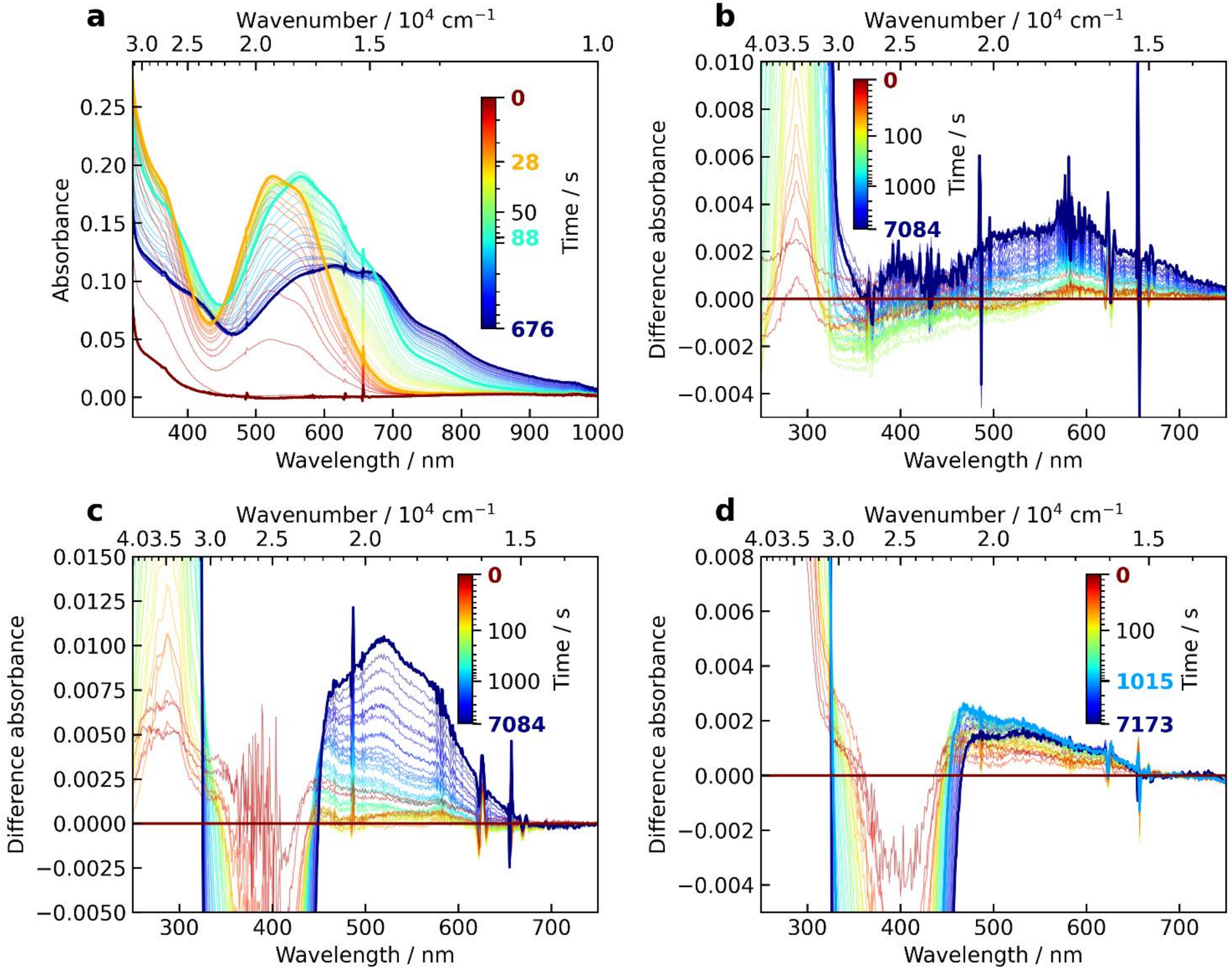

Detection of Superoxide During Singlet Oxygen Oxidation of *Z*-13 in methanol

The ability to produce singlet oxygen thermally allows for testing whether $^1O_2$ can react with *Z*-**13** by electron transfer reaction to produce superoxide ($O_2^{\bullet-}$), because electron transfer reactions cannot be simply avoided during dye-sensitized reactions. To test whether free superoxide (pathway *iii* in Scheme 2, main text) is produced to some extent during the reaction, we used Nitro Blue Tetrazolium (NBT), which is selectively reduced by $O_2^{\bullet-}$ to monoformazan, visible at ~530 nm.[5] We also independently spectroscopically identified monoformazan by the reduction of NBT with sodium ascorbate in methanol (Figure S48a, orange spectrum at 28 s, due to low solubility of mono and diformazan, it started to precipitate from the solution). We detected small signals from monoformazan upon incubation of *Z*-**13** and $DMNO_2$ with NBT in methanol (Figure S48c). Negligible signals were detected in a blank experiment (only $DMNO_2$ and NBT, Figure S48b). This may suggest that superoxide is produced. In the mixture of methanol and water (1:1), no or negligible amount of formazan was observed (Figure S48d). Previously, reserchers[6,7] used superoxide dismutase as a competitive reagent to NBT, which caused fast disproportionation of superoxide to estimate the amount of formazan produced by the free superoxide. Unfortunately, *Z*-**13** is not soluble in water, and superoxide dismutase cannot be used in methanol. Because we lack such a competing reagent in methanol, we cannot be sure whether the detected formazan was produced by free superoxide. NBT could possibly be reduced by some products or reaction intermediates resulting from oxidation of *Z*-**13**. Nevertheless, oxidation potentials of several alkyl-substituted dipyrrinones (E_{ox} ~ 0.52 – 0.65 V vs SCE)[8] are comparable to the reduction potential of singlet oxygen (E_{red} ~ 0.39 V vs SCE),[9] thus electron transfer should theoretically be feasible. From our experiments, we cannot determine whether superoxide is produced or not.

Figure S49. Sensitization of *Z*-**13** (c ~ 27 μmol L^{-1}) with RB (c ~ 3 μmol L^{-1}) in methanol followed by ESI-MS. Traces corresponding to sodium adducts of the various *m/z* values are displayed (the traces were normalized to TIC).

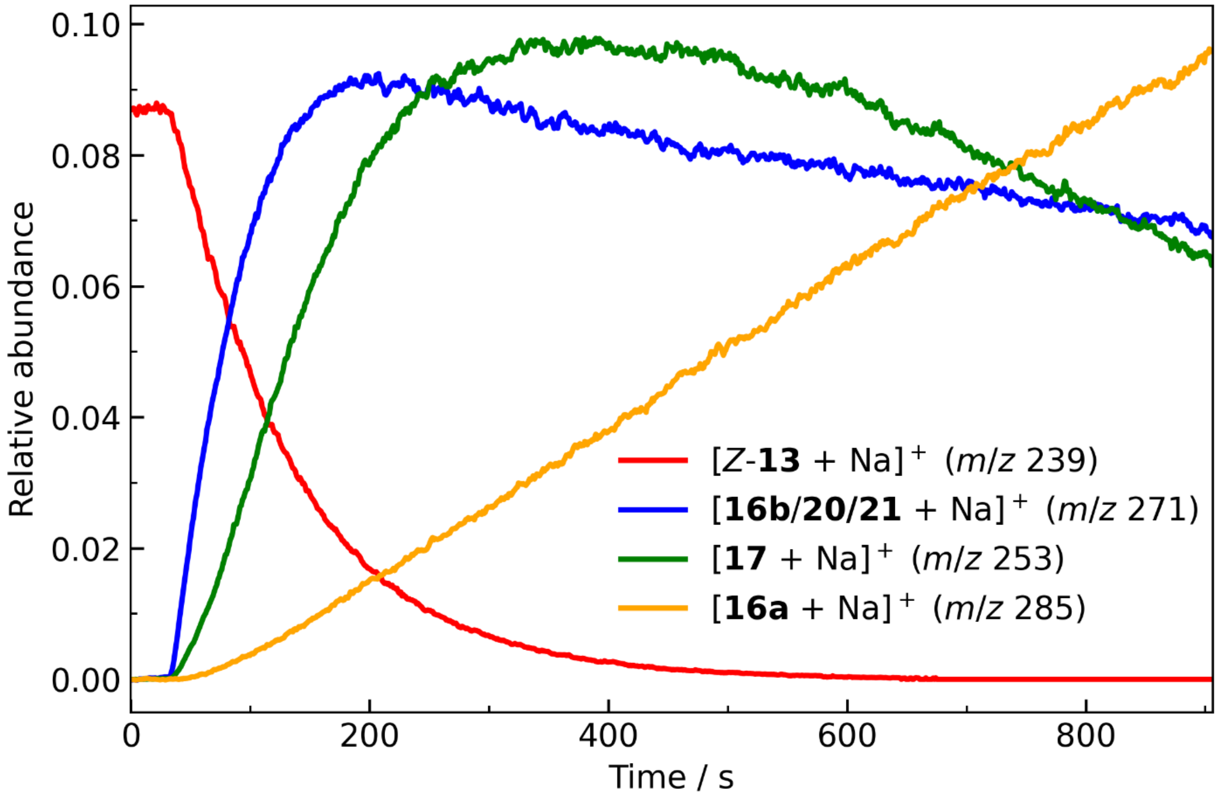

Figure S50. CID experiment showing release of oxygen molecule from sodium aduct of **DMNO₂** in methanol ([**DMNO₂** + Na]$^+$, *m/z* 211).

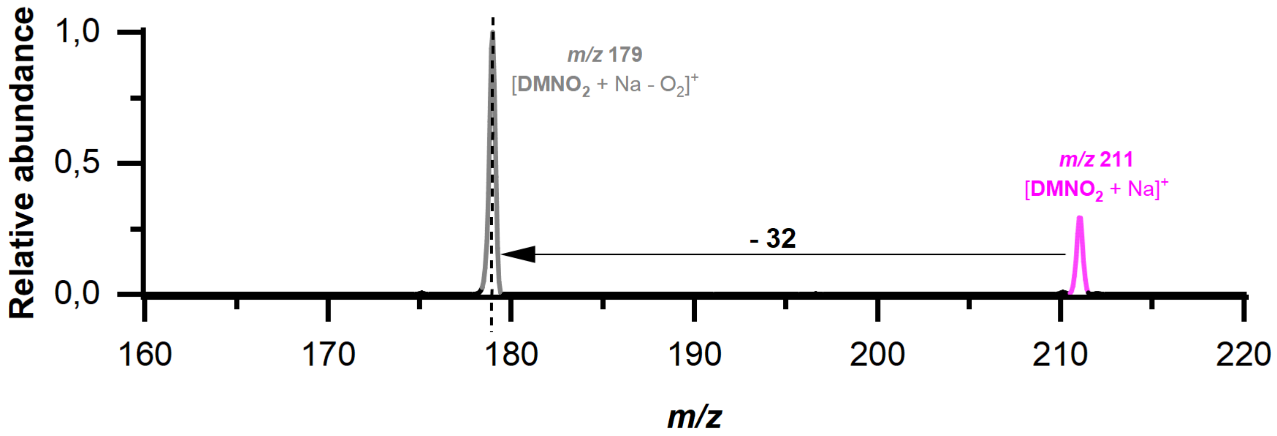

Figure S51. ESI-MS spectra of RB-sensitized irradiation of *Z*-**13** in CD_3OD. (a) Before irradiation, (b) after 4 min, (c) after 10 min of irradiation of the sample at 518 nm LED.

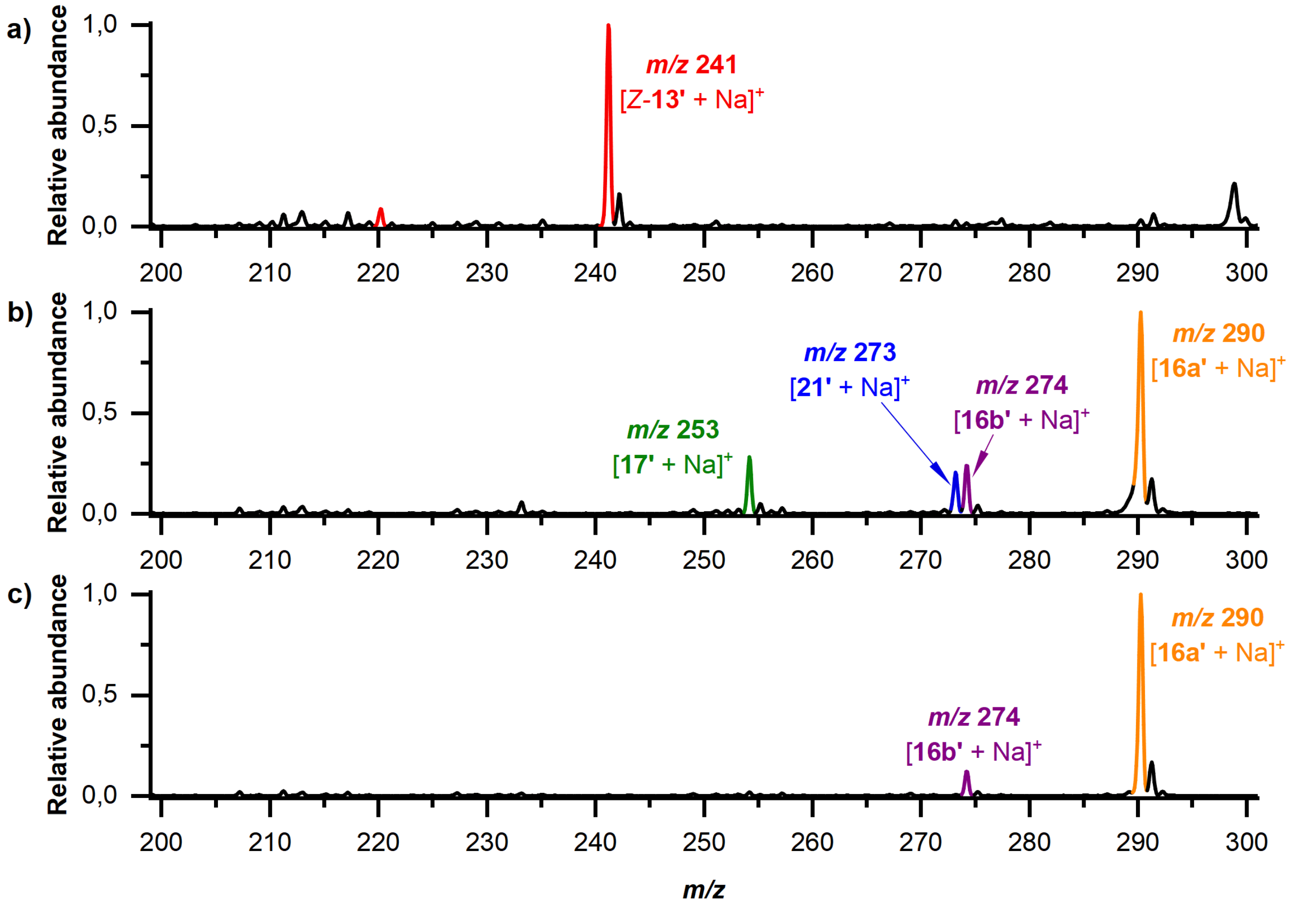

Figure S52. CID mass spectra of deuterated aducts of (a) **21’** ([**21’** + D]$^+$, *m/z* 252) and (b) **16b’** ([**16b’** + D]$^+$, *m/z* 253) from RB-sensitized irradiation of *Z*-**13** in CD_3OD.

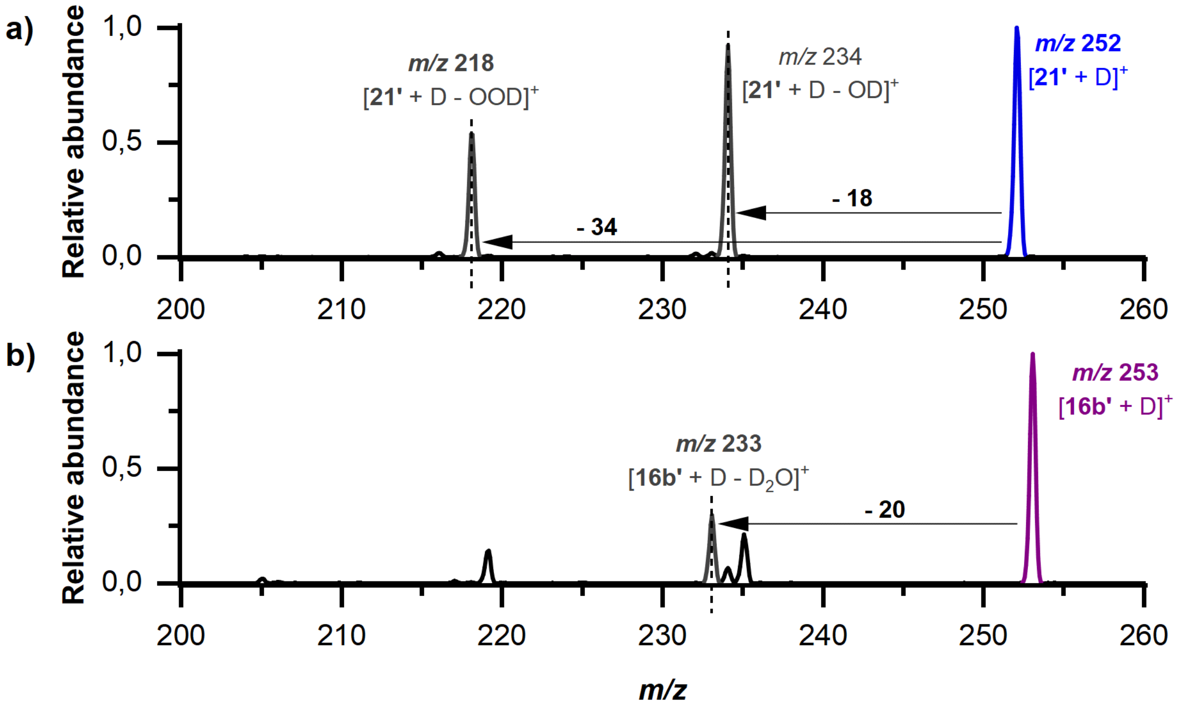

Figure S53. CID mass spectra of sodium aducts of (a) **21'** ([**21'** + Na]$^+$, *m/z* 273) and (b) **16b'** ([**16b'** + Na]$^+$, *m/z* 274) from RB-sensitized irradiation of *Z*-**13** in CD_3OD.

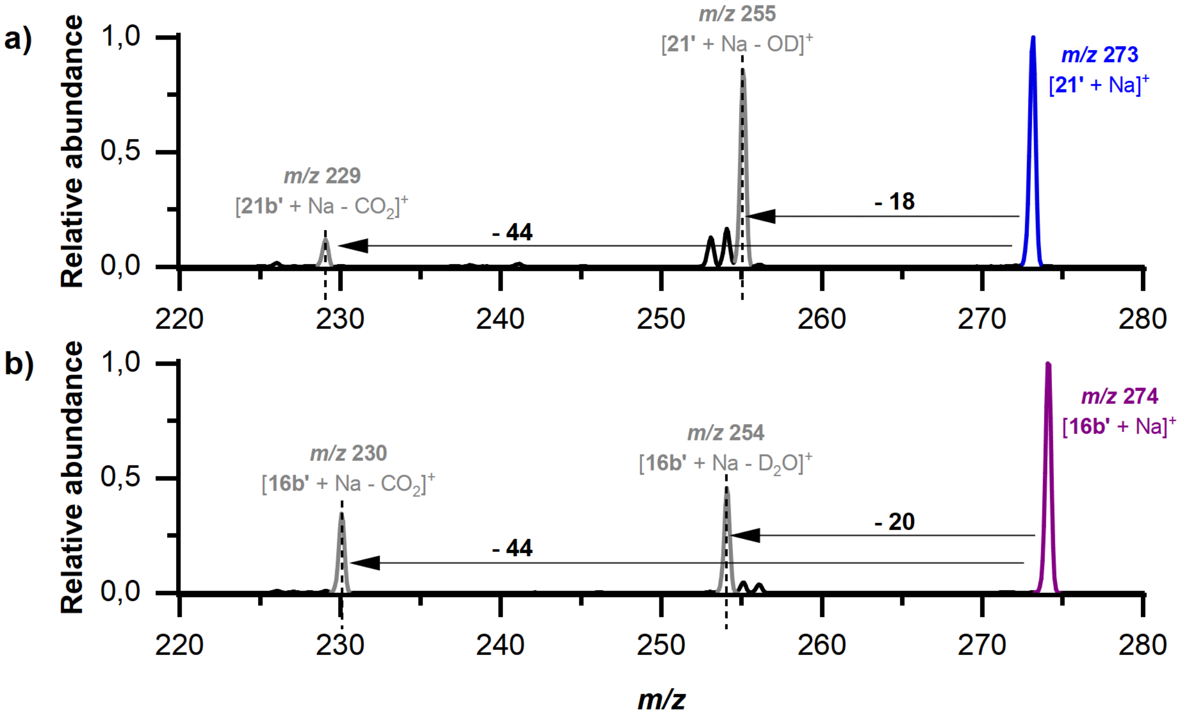

Kinetics and CID Measurements of Photooxidation of *Z*-13 in CD_3OD

Mass spetra from the RB-sensitized irradiation of *Z*-**13** in CD_3OD, analyzed using ESI-MS, are presented in Figure S51. Before irradiation, the spectrum shows only the ion [*Z*-**13**' + Na]$^+$ (*m/z* 241). After irradiating the reaction mixture with a 518 nm LED for 4 minutes (Figure S51b), new sodium adducts are detected at *m/z* 253 ([**17'** + Na]$^+$), *m/z* 273 ([**21'** + Na]$^+$), *m/z* 274 ([**16b'** + Na]$^+$) and *m/z* 290 ([**16a'** + Na]$^+$). Upon examining the reaction mixture again at the 10-minute mark (Figure S51c), only two ions remained: *m/z* 274 ([**16b'** + Na]$^+$) and *m/z* 290 ([**16a'** + Na]$^+$). This confirms that **16a'** and **16b'** are the final products, while **17'** and **21'** serve as intermediates.

The results of CID measurements of a mass-selected ion at *m/z* 252 in deuterated methanol indicated that a deuterated hydroxyl group/radical and a deuterated hydroperoxy group/radical were fragmented from the adduct of **21'** with deuterium ([**21'** + D]$^+$, *m/z* 252, Figure S52a). Additionally, the analysis of a mass-selected ion at *m/z* 253 (corresponding to [**16b'** + D]$^+$, Figure S52b) revealed the fragment resulting from the cleavage of D_2O ([**16'** + D – D_2O]$^+$, *m/z* 233). The obtained fragments in CID mass spectra were formed by the release of the same species as those from the protonated adducts of **16** or **21**, but in this case, they were the corresponding to their deuterated analogs.

Aside from trying to understand the fragmentation of deuterated adducts with target species, we also investigated their corresponding sodium adducts in CD_3OD. However, upon dissociation, the ions [**16b'/21'** + Na]$^+$ and [**21'** + Na]$^+$ did not exhibit the loss of the hydroperoxy group/radical and the deuterated hydroperoxy group/radical, respectively (Figure S53). Instead, both ions showed cleavage of CO_2 (44 Da). Unfortunately, the exact mechanism behind this fragmentation was not determined, but we assumed that it might occur *via* a rearrangement and ring-opening process. The other fragments generated from sodium adducts were found to be identical to those formed from the protonated or deuterated adducts.

Figure S54. Reaction of *Z*-**13** with thermally produced singlet oxygen by **DMNO2** in methanol. CID mass spectrum showing fragmentation of mass-selected [**16b**/**21a** + H]$^+$ (*m/z* 249).

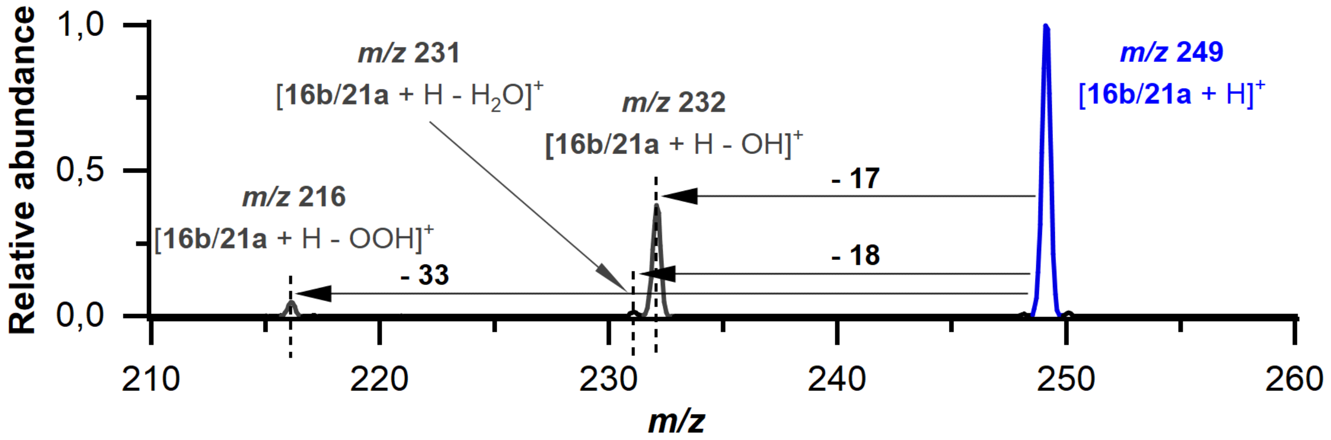

Figure S55. (a) Comparison of the experimental helium tagging IRPD spectrum of the ion with *m/z* 252 with the theoretical IR spectrum for (b) [**20’** + D]$^+$, (c) [**21a’** + D]$^+$ and (d) [**21b’** + D]$^+$ Theoretical IR spectra were calculated using B3LYP/6–311+G**, GD3BJ, and the scaling factor of 0.98 for v < 2000 cm^{-1} and 0.96 for v > 2000 cm^{-1}.

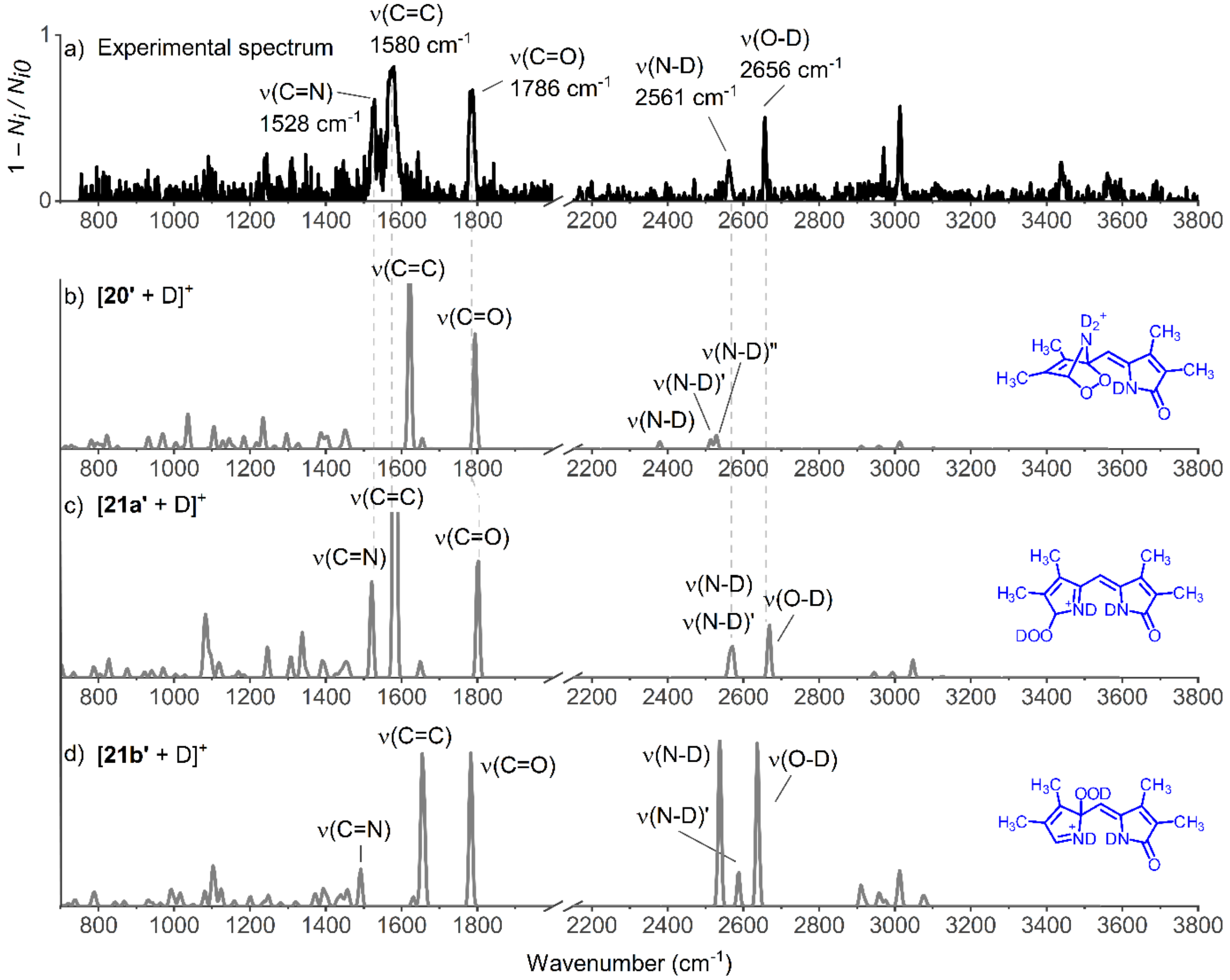

Figure S56. Photochemical Irradiation Methods. (A) The vial containing *Z*-**13** in methanol with rose bengal (RB) is irradiated with green LED light (518 nm, Figure S64) to monitor photochemical reactions over time. This approach was employed for ESI-MS reaction monitoring and collision-induced dissociation (CID) experiments. (B) A section of the capillary towards the mass spectrometer is irradiated with green LED light (518 nm, Figure S64) while the vial remains in the dark, maintaining a constant snapshot of short-lived intermediates. This method was used for IRPD measurements.

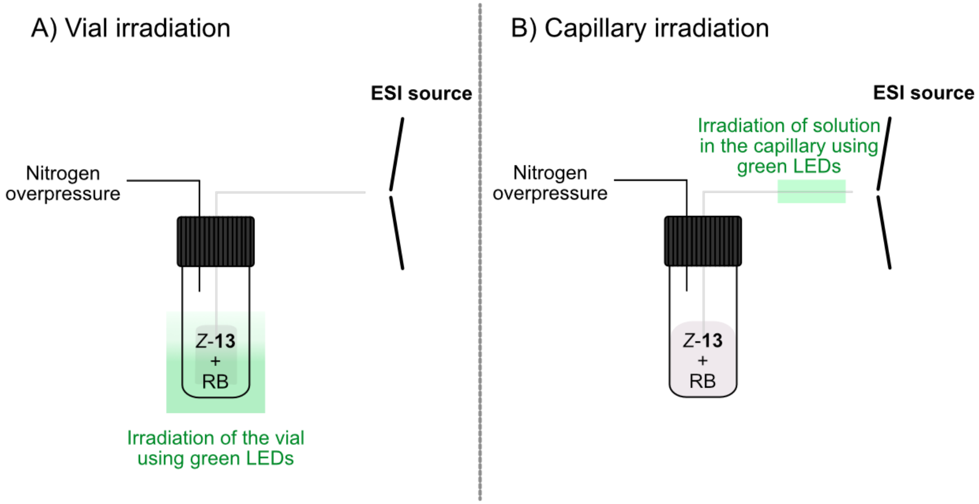

Fitting of MS Kinetic Data

The kinetic traces were simulated for 4 species (**16b**, **21a**, **17** and **16a**) by numerical integration of the following matrix differential equation,

$$\frac{\mathrm{d}\boldsymbol{c}(t)}{\mathrm{d}t} = \mathbf{K}\boldsymbol{c}(t) + \boldsymbol{j}(t)$$

where $\boldsymbol{c}(t)$ are the concentration profiles, $\boldsymbol{j}(t)$ is the initial concentration vector, and **K** is the transfer matrix describing the model. The concentration profile of the starting material *Z*-**13** was not simulated due to the difficulty of modeling the photokinetic decay. Instead, the experimental *m/z* trace of *Z*-**13** was first smoothed (by Whittaker smoother[10]) to remove an instrumental noise (denoted as $m/z'_{Z\text{-}\mathbf{13}}(t)$), and then, the decay rate was calculated by differentiation. This rate was then used to solve the matrix equation during the numerical integration. The kinetic model, as described in the main text, was modeled by the following expressions,

$$\boldsymbol{c}(t) = \begin{pmatrix} c_{\mathbf{16b}}(t) \\ c_{\mathbf{21a}}(t) \\ c_{\mathbf{17}}(t) \\ c_{\mathbf{16a}}(t) \end{pmatrix} \quad \boldsymbol{j}(t) = \begin{pmatrix} -\alpha\left(\frac{\Delta m/z'_{Z\text{-}\mathbf{13}}(t)}{\Delta t}\right)_t \\ -(1-\alpha)\left(\frac{\Delta m/z'_{Z\text{-}\mathbf{13}}(t)}{\Delta t}\right)_t \\ 0 \\ 0 \end{pmatrix} \quad \mathbf{K} = \begin{bmatrix} 0 & 0 & 0 & 0 \\ 0 & -k_1 & 0 & 0 \\ 0 & k_1 & -k_2 & 0 \\ 0 & 0 & k_2 & 0 \end{bmatrix}$$

where $\alpha = 0.5$ is the branching coefficient, which describes how much **16b** is formed compared to **21a**, and it was kept fixed. The experimental *m/z* kinetic traces were then modeled from the simulated concentration profiles by multiplying them with 4 amplitudes as additional fitting parameters ($m/z_i(t) = a_i c_i(t)$). Two unknown rate constants and four amplitudes were obtained by the nonlinear least squares fitting procedure (Python's LMFIT package[11,12]).

For the alternative kinetic model shown on the next page (Figure S57), the following expressions were used:

$$\boldsymbol{c}(t) = \begin{pmatrix} c_{\mathbf{16b}}(t) \\ c_{\mathbf{21a}}(t) \\ c_{\mathbf{17}}(t) \\ c_{\mathbf{16a}}(t) \end{pmatrix} \quad \boldsymbol{j}(t) = \begin{pmatrix} 0 \\ -\left(\frac{\Delta m/z'_{Z\text{-}\mathbf{13}}(t)}{\Delta t}\right)_t \\ 0 \\ 0 \end{pmatrix} \quad \mathbf{K} = \begin{bmatrix} 0 & \alpha k_1 & 0 & 0 \\ 0 & -k_1 & 0 & 0 \\ 0 & (1-\alpha)k_1 & -k_2 & 0 \\ 0 & 0 & k_2 & 0 \end{bmatrix}$$

Figure S57. The traces (solid lines) of the ions formed during the dye-sensitized photooxidation of *Z*-**13** in CD_3OD. Dashed lines show the fitting of an alternative kinetic model as depicted. The fitted curve corresponding to **16b'** does not match the experimental one.

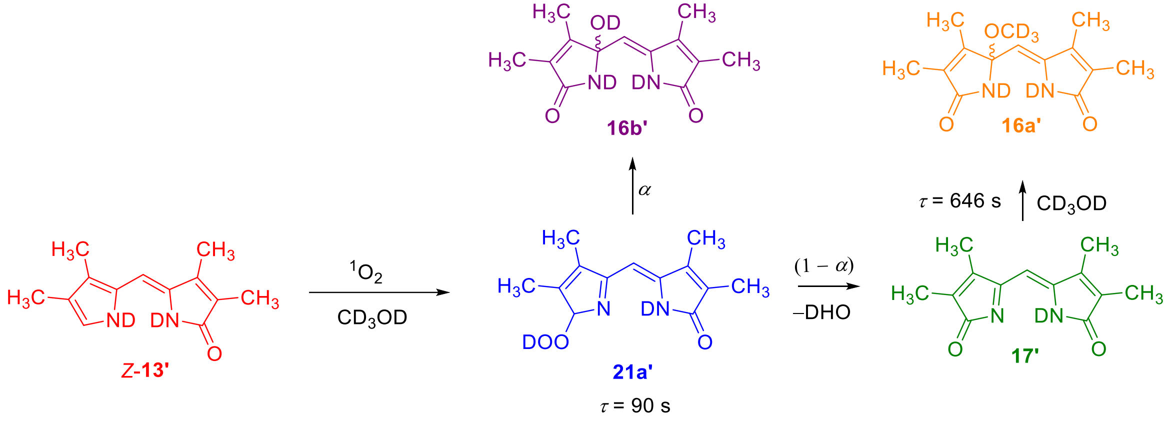

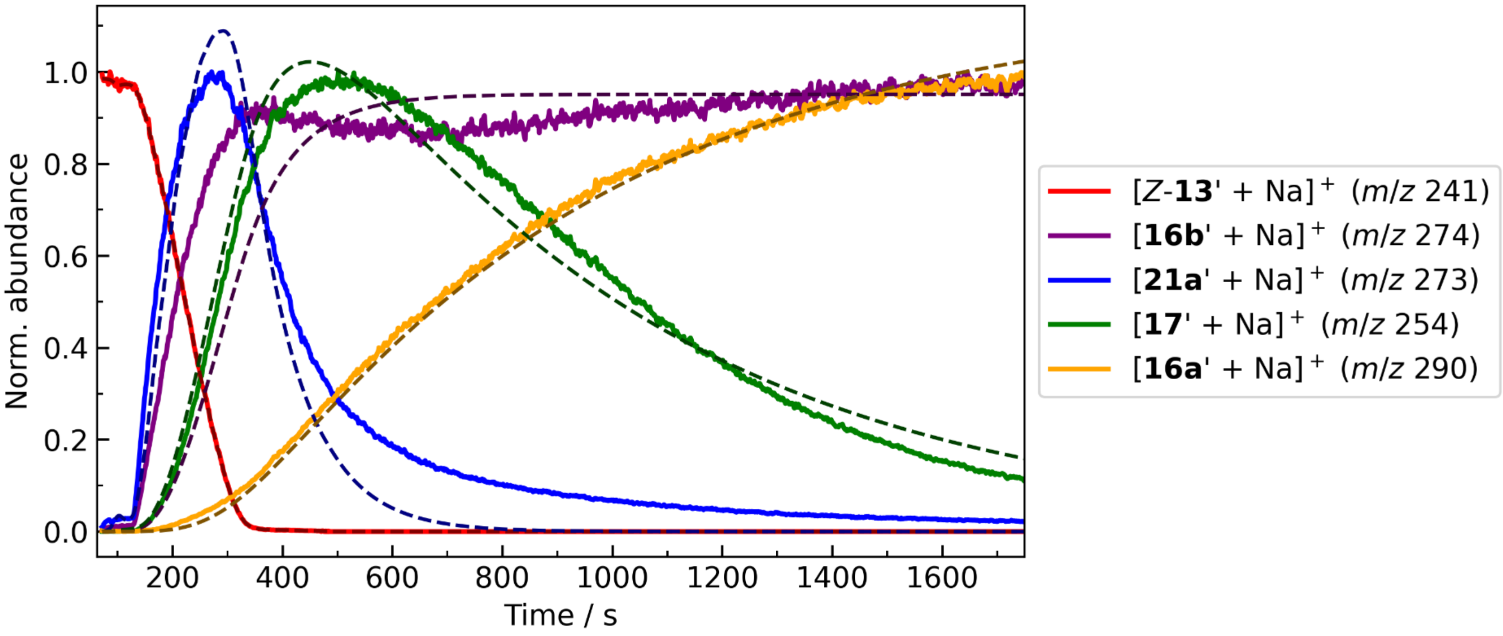

Extended Discussion on the MS Kinetics Data in CD_3OD

Endoperoxide **20** can open on both sides, producing **13a** or **13b** (see Figure 5). From our results, **21a** is probably formed predominantly; however, it is possible that **21b** is also produced but as a minor product. This may be supported by the fact that the experimental trace corresponding to **21'** (*m/*z 252, blue line, Figure 7) deviates at later stages of the reaction (> 450 s) from the proposed model (Figure 7). The experimental trace still has a minor population, while the fitted trace reaches zero. A similar but opposite trend can be observed in the trace corresponding to **16b'** (*m/*z 253, purple line, Figure 7) at later stages of the reaction (>450 s). On the first look, this increase seems to be correlated with the decrease of the *m/*z 252 trace. Thus, the *m/*z 252 ion could consist of both **21a** and **21b**, in which **21a** is a major and **21b** is a minor product. **21b** would likely possess a longer lifetime than **21a** because it cannot undergo dehydration or tautomeric rearrangement as effectively as **21a** can (see main text). The longer lifetime of **21b** could explain the observed experimental trace of *m/*z 252. At the same time, the formation of some other propentdyopent-type products with the *m/*z 253 mass could explain a slight rise of the trace *m/*z 253 (purple line, Figure 7) at the late stages of the reaction.

Global Fitting of UV-Vis Kinetic Data of *Z*-13 Sensitization in Methanol Using the Best Target Model from MS Kinetics in CD_3OD

The kinetic traces were simulated similarly to the MS kinetic model by numerical integration of the following matrix differential equation,

$$\frac{\mathrm{d}\boldsymbol{c}(t)}{\mathrm{d}t} = \mathbf{K}\boldsymbol{c}(t) + \boldsymbol{j}(t) \qquad \boldsymbol{c}(t) = \begin{pmatrix} c_{Z\text{-}\mathbf{13}}(t) \\ c_{\mathbf{16a+16b}}(t) \\ c_{\mathbf{21a}}(t) \\ c_{\mathbf{17}}(t) \end{pmatrix}$$

where ***c***(*t*) are the concentration profiles, ***j***(*t*) is the initial concentration vector, and **K** is the transfer matrix describing the model. Due to the similarity of the absorption spectra of propentdyopents **16a** and **16b**, it is impossible to resolve them by global fitting of UV-Vis kinetics. Therefore, they were modeled as single species $c_{\mathbf{16a+16b}}(t)$. The decay of *Z*-**13** was modeled by a photokinetic model where the singlet oxygen produced by irradiation of the sensitizer (RB) then reacts with the substrate *Z*-**13**,

$$-v_{Z\text{-}\mathbf{13}}(t) = \frac{\mathrm{d}c_{Z\text{-}\mathbf{13}}(t)}{\mathrm{d}t} = -\frac{\Phi_\Delta k_r c_{Z\text{-}\mathbf{13}}(t)}{k_d + (k_q + k_r)c_{Z\text{-}\mathbf{13}}(t)} \int \left(1 - 10^{-A(\lambda,t)}\right) I_{LED}(\lambda)\mathrm{d}\lambda$$

where $k_r = 10^9$ s^{-1} M^{-1} is the rate constant of reaction of singlet oxygen with *Z*-**13**, $k_q = 2.3 \times 10^9$ s^{-1} M^{-1} is the quenching rate constant of singlet oxygen by *Z*-**13**, $k_d = 1.053 \times 10^5$ s^{-1} is the decay rate constant of singlet oxygen in methanol,[13] $\Phi_\Delta = 0.76$[14] is the singlet oxygen production quantum yield of RB, and $I_{LED}(\lambda)$ is the incident spectral flux that hits the cuvette. The integrated intensity was allowed to be varied during fitting because the incident photon flux was not determined beforehand. The kinetic model, as described in the main text, was modeled by the following expressions,

$$\boldsymbol{j}(t) = \begin{pmatrix} -v_{Z\text{-}\mathbf{13}}(t) \\ \alpha v_{Z\text{-}\mathbf{13}}(t) \\ (1-\alpha)v_{Z\text{-}\mathbf{13}}(t) \\ 0 \end{pmatrix} \qquad \mathbf{K} = \begin{bmatrix} 0 & 0 & 0 & 0 \\ 0 & -k_3 & 0 & k_2 \\ 0 & 0 & -k_1 & 0 \\ 0 & 0 & k_1 & -k_2 \end{bmatrix}$$

where $\alpha = 0.3$ is the branching coefficient, which describes how much **16b** is formed compared to **21a**, and it was estimated from the ratio of products (**16b** + **19**) and **16a** and was kept fixed. The initial concentration of *Z*-**13** was calculated from the absorbance data and used as the initial condition for numerical integration. During the fitting routine, the spectra were calculated using the least squares from the concentration profiles,

and the data were recorded using a variable projection algorithm.[15] The spectra of unknown species and three rate constants were then obtained by a nonlinear least squares fitting procedure (use of Python's LMFIT package[11,12]). Lifetimes of 83 s ($1/k_1$), 257 s ($1/k_2$), and 21 800 s ($1/k_3$) were obtained. The first lifetime corresponds to the lifetime of **21a**, while the second one is the lifetime of **17**. The last long lifetime was added to the model to obtain a good fit at longer times, and it has no significance for quick kinetics in the beginning. The lifetimes obtained are slightly lower than those obtained from fitting the MS traces in CD_3OD (see main text), but that is expected due to the solvent kinetic effects. The obtained Species-Associated Spectrum (SAS) for **21a** (blue line, Figure S58b) shows only one peak at ~310 nm, corresponding to its absorption maximum as compared to two peaks (~277 nm and ~310 nm) in the mixture of **21a** and **16b** at early times of the kinetics (Figure S58, 8 s, orange line). Furthermore, the SAS corresponding to **17** (green line, Figure S58) is much better resolved and contains the main absorption peak at ~360 nm with a tail at longer wavelengths, which qualitatively agrees with the experimental spectrum of **17** in dichloromethane (see Figure 3b in main text or Figure S58).

Figure S58. (a) Sensitization of *Z*-**13** (c ~ 43 μmol L^{-1}) with RB (c ~ 7 μmol L^{-1}) under an oxygen atmosphere with 532 nm LEDs in methanol as also depicted in Figure 4a in the main text, and its global fitting results from the target model described on the previous page. (b) Species-associated spectra, (c) concentration profiles, and (d) experimental (solid lines) and fitted traces (dashed lines) at different wavelengths.

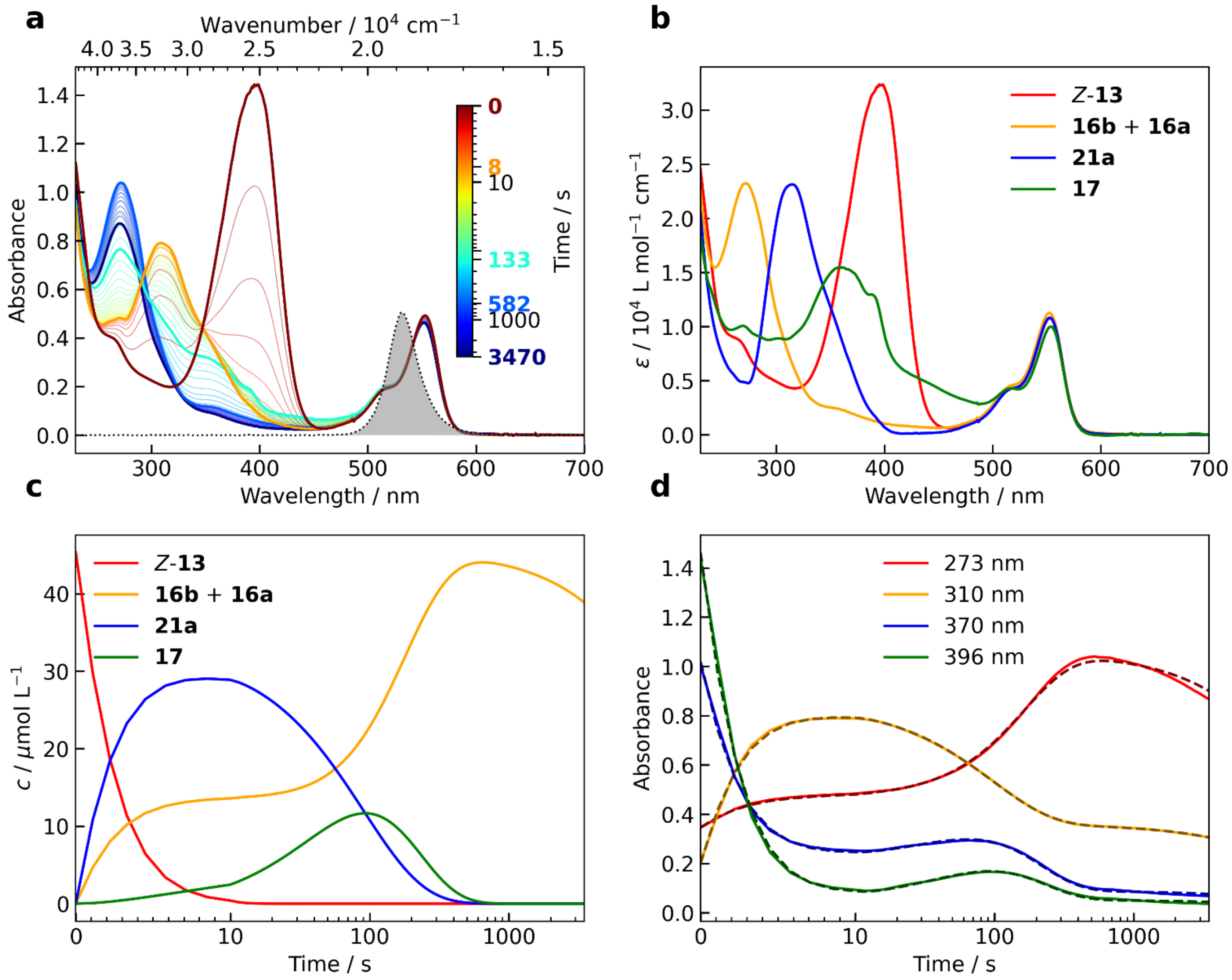

Figure S59. Determination of the reaction and quenching rate constants of singlet oxygen by (a) *E*-**13** in methanol and (b) *Z*-**13** in acetonitrile by initial rate method described in ref [16] using **DMNO2** as singlet oxygen source (T = 25 °C).

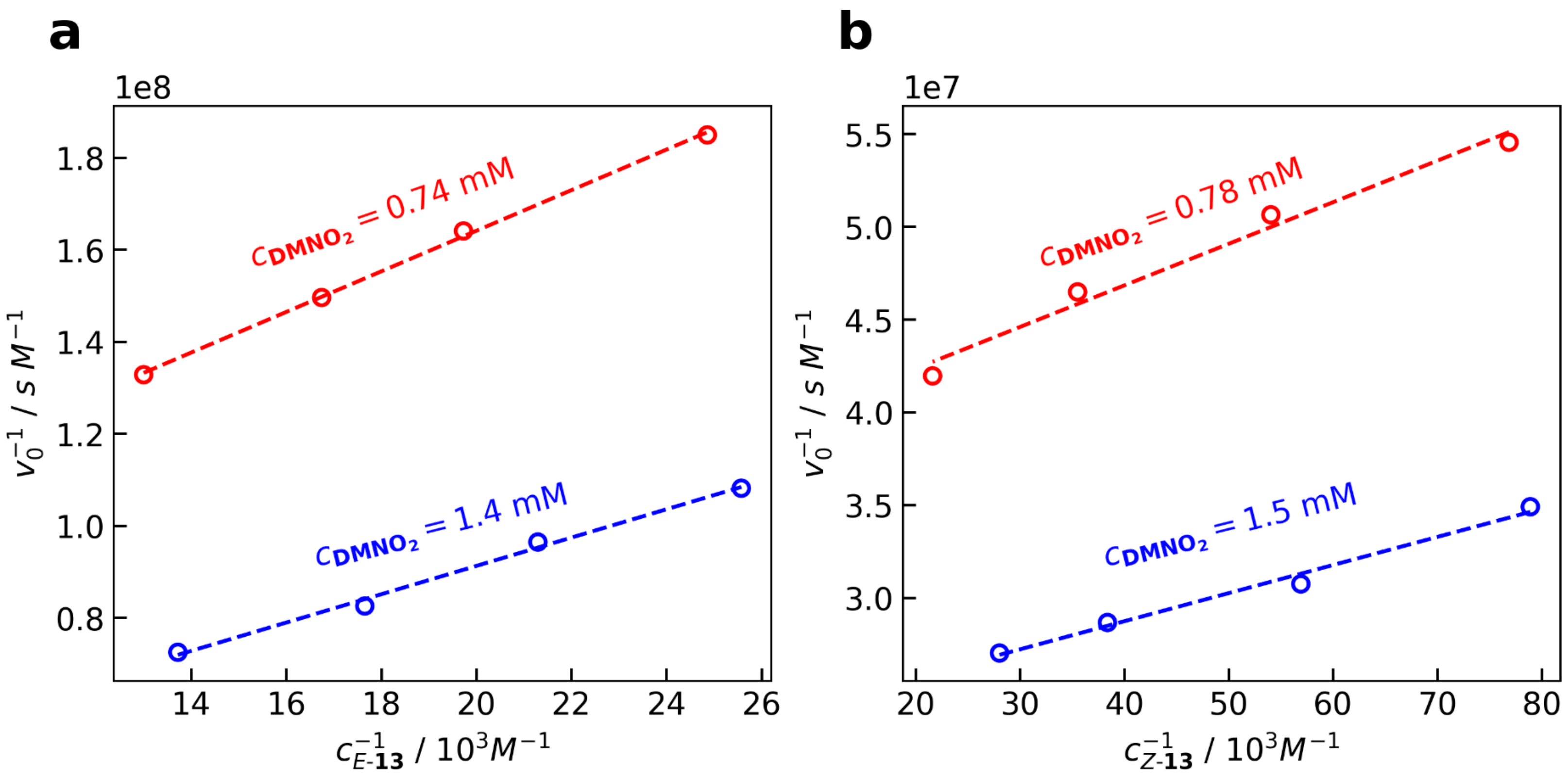

Table S3. Reaction (k_r) and quenching (k_q) rate constants for *Z*-**13** and *E*-**13** in the presence of singlet oxygen in methanol and acetonitrile determined by initial rate method from ref [16]. k_{sq} represents self-quenching rate constant of singlet oxygen by **DMNO₂**. The lifetime of singlet oxygen in methanol which was used to determine the below given constants was $\tau = 9.5$ μs.[13]

Comp.	solvent	k_q / 10^9 s^{-1} M^{-1}	k_r / 10^9 s^{-1} M^{-1}	k_{sq} / 10^7 s^{-1} M^{-1}
Z-**13**	MeOH	2.32	0.992	6.62
Z-**13**	MeCN	1.48	2.09	7.43
E-**13**	MeOH	2.24	0.622	8.42

Figure S60. HRMS (APCI$^+$) of **16a**. Molecular peak was not observed, however, iminium **17H**$^+$ ([**17** + H]$^+$, *m/z* 231.1130), proton aduct of its dimer ([**17** + **17** + H]$^+$, *m/z* 461.2178) and, proton aduct of **16a** and **17** [**16a** + **17** + H]$^+$, *m/z* 493.2446) were detected.

APCI + (MMI)

nitrogen flow 5 L/min, gas temperature 325°C, nebulizer 45 psig, skimmer 65 V, vaporizer 200°C, fragmentor 10 V, dissolved in methanol

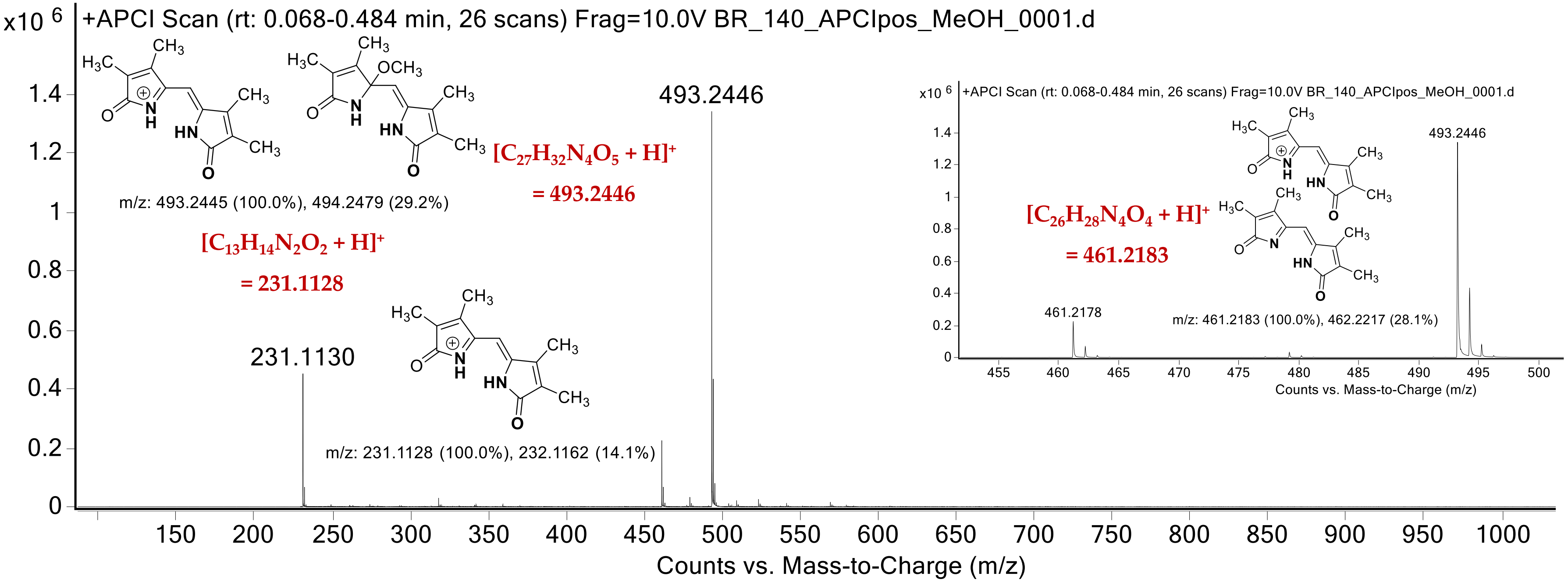

expected mass: $[M+H]^+ = 263.1390$ not observed

Figure S61. HRMS (APCI$^+$) of mixture of **19** and **16b**. Molecular peak was observed, however, it does not prove the presence of **19** as it is in the mixture with **16b** which possess the same mass.

APCI + (MMI)

nitrogen flow 5 L/min, gas temperature 325°C, nebulizer 45 psig, skimmer 65 V, vaporizer 200°C, fragmentor 10 V, dissolved in methanol

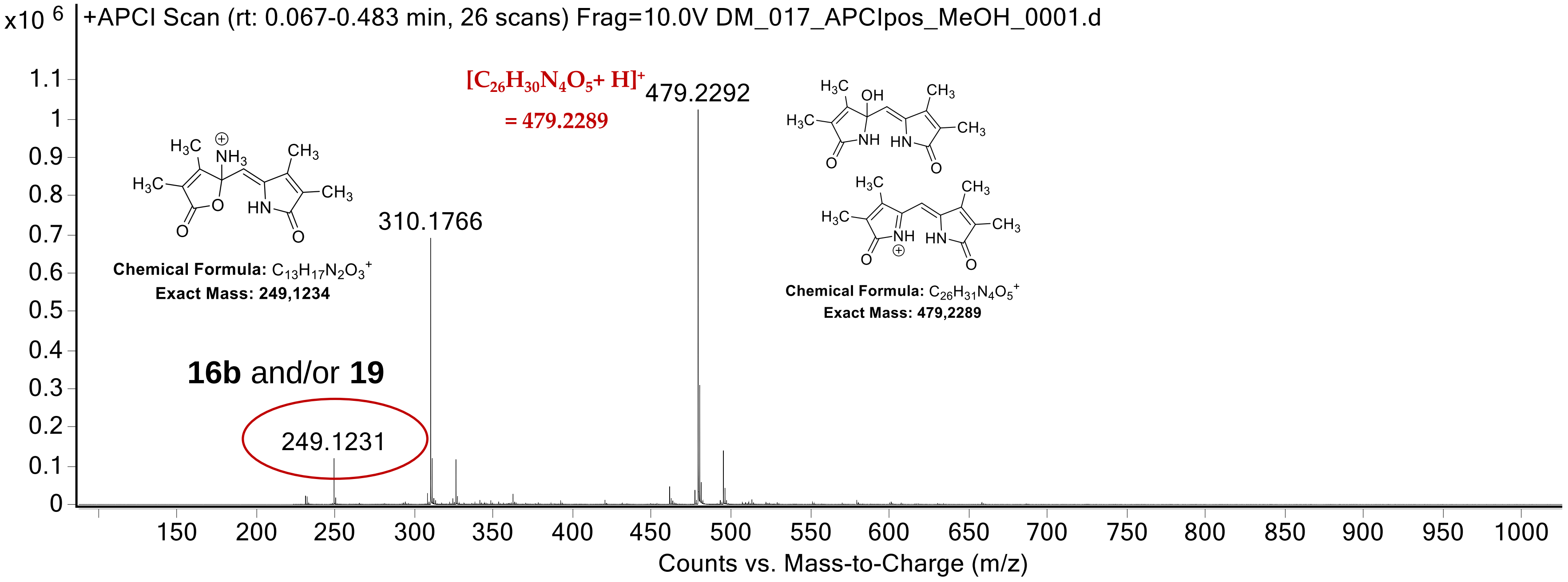

expected mass: $[M+H]^+ = 249.1234$ observed mass : $[M+H]^+ = 249.1231$ mass accuracy = - 1.2 ppm

Figure S62. A custom-made 3D-printed LED reactor (two plug-in LED modules, 14 low-power LEDs per module, an integrated cooling fan, and a stirring pad with adjustable speed) used for simultaneous irradiation and UV-vis spectroscopy measurements of the samples in 10.0 mm cuvettes.

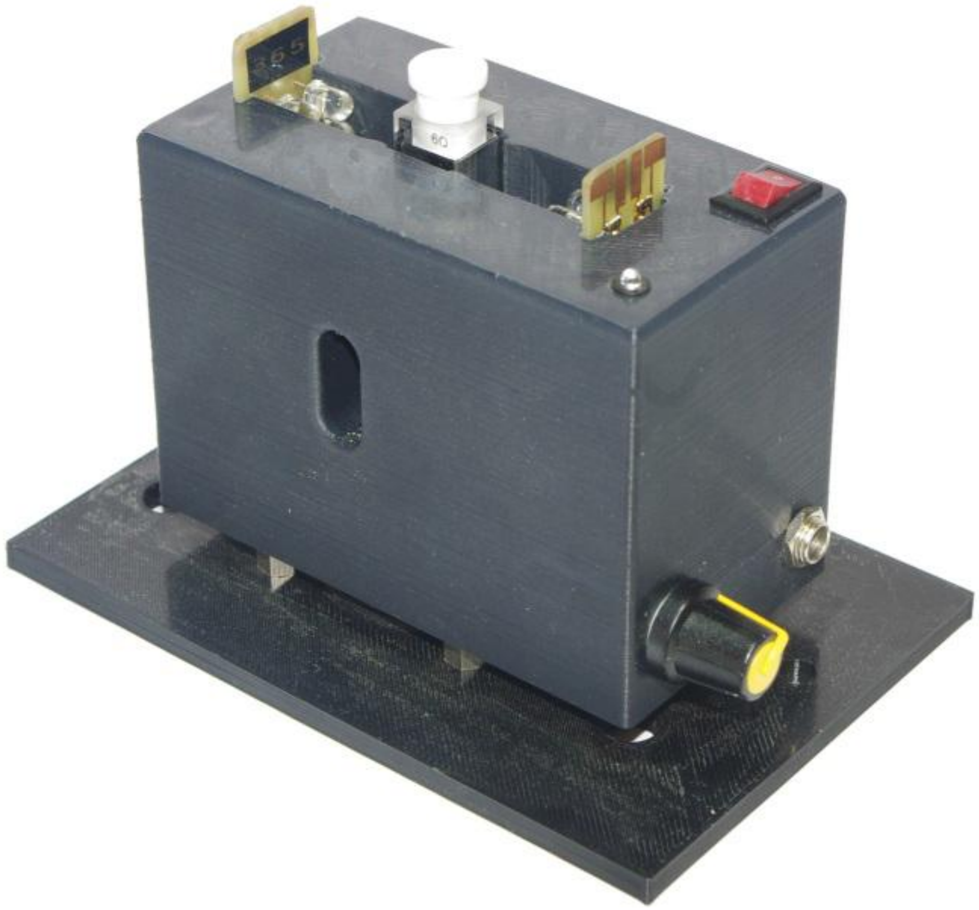

Figure S63. Experimental setup for the preparation of 1,4-dimethylnaphthalene endoperoxide (**DMNO$_2$**). The long cylindrical flask is being cooled with an ice bath at the bottom part while being irradiated with three 100 W white LED reflectors and purging the solution with oxygen.

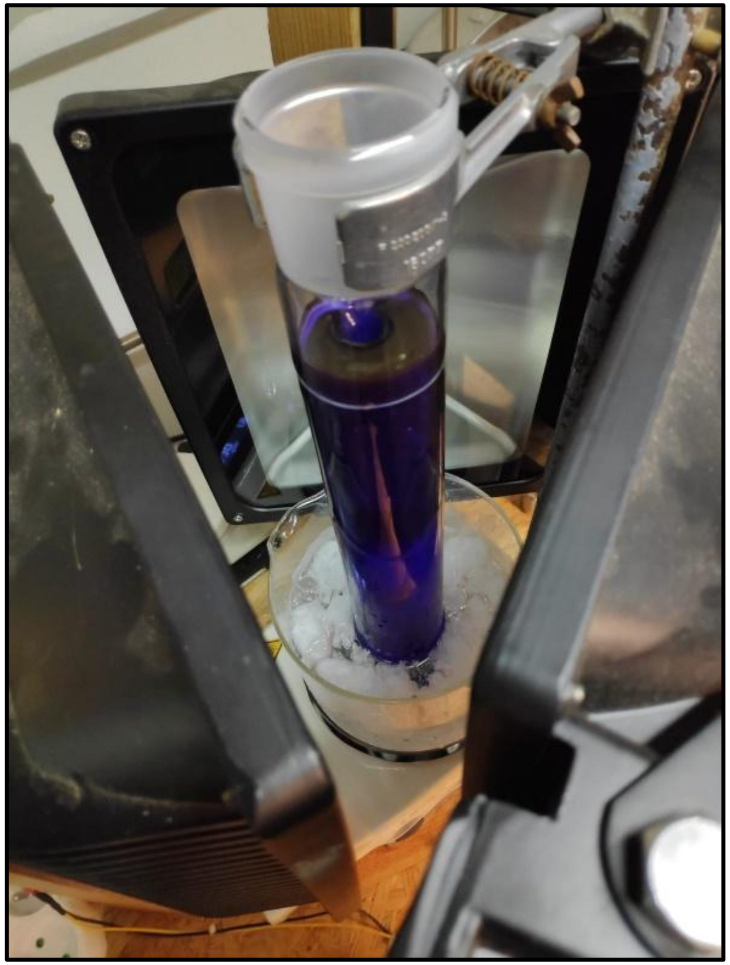

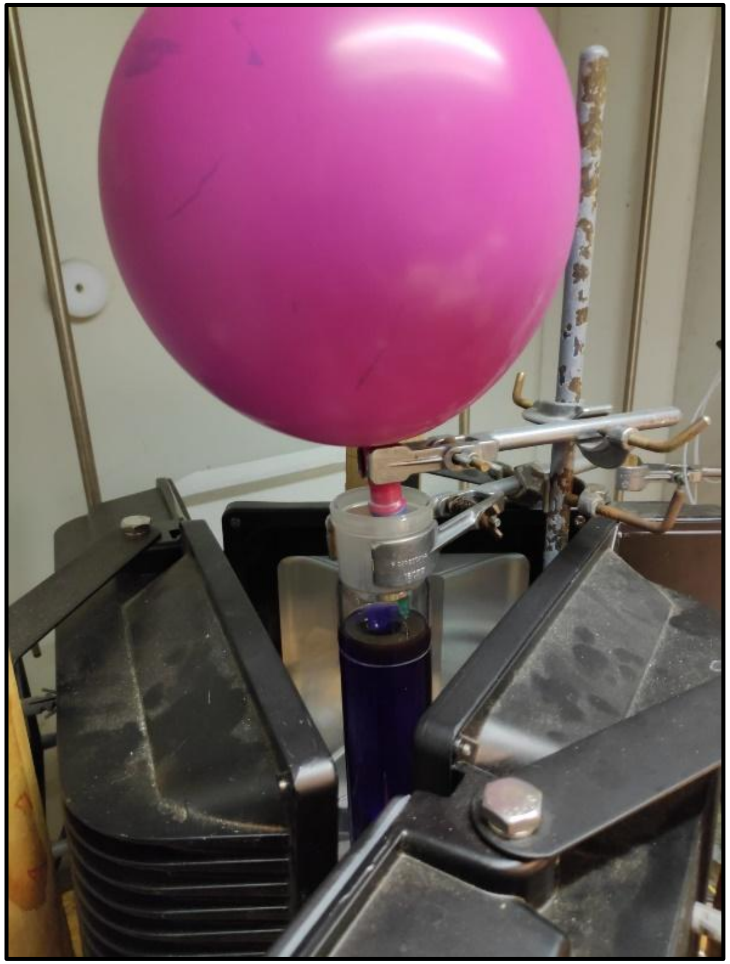

Figure S64. Emission spectra of LEDs used for all irradiation experiments. The spectra are normalized to the unit area $\left(\int PDF(\lambda)\mathrm{d}\lambda = 1\right)$. PDF = Probability density function.

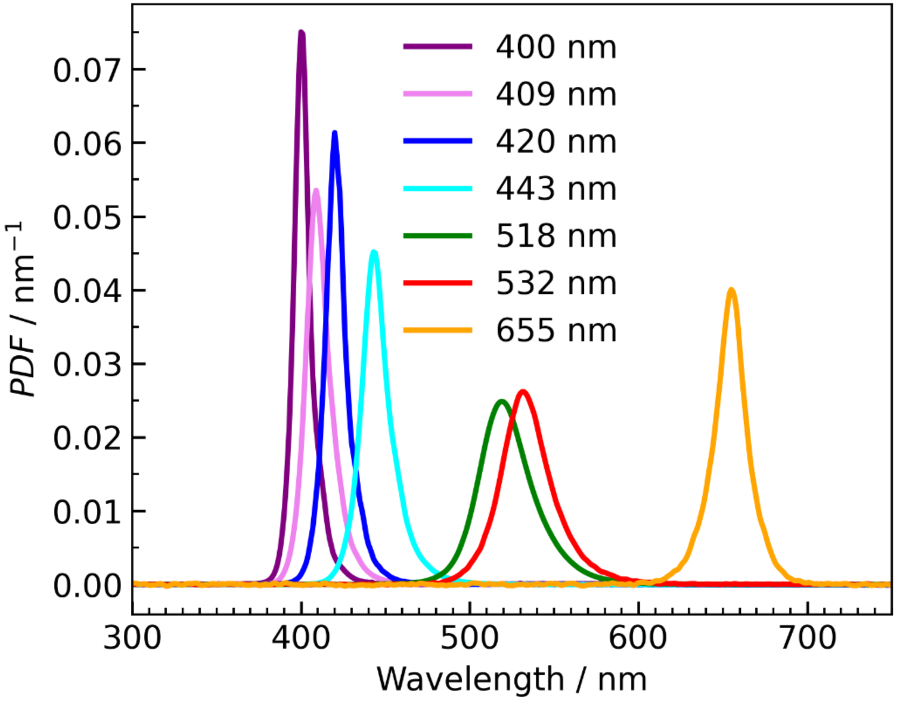

Calculated geometries with B3LYP/6–311+G, GD3BJ**.

[**20** + H]+
Sum of electronic and zero-point energies: -840.446985

C	1.278833000	0.375587000	-0.065480000
N	1.511143000	-0.988610000	-0.122864000
C	2.914843000	-1.223526000	-0.127986000
C	3.554152000	0.112608000	0.076261000
C	2.581902000	1.052274000	0.092323000
C	2.703447000	2.529671000	0.261755000
C	5.027408000	0.231023000	0.216614000
O	3.425939000	-2.299384000	-0.280105000
C	0.059323000	0.972836000	-0.072813000
C	-1.191183000	0.222282000	-0.071156000
C	-2.543328000	0.906550000	-0.103462000
C	-3.461162000	0.003644000	0.285711000
C	-2.704654000	-1.271612000	0.584781000
C	-2.720794000	2.274438000	-0.667045000
C	-4.949275000	0.053732000	0.282848000
N	-1.440366000	-0.742166000	1.206959000
O	-1.170706000	-0.825525000	-1.056535000
O	-2.173617000	-1.826283000	-0.616164000
H	0.907055000	-1.608129000	-0.647652000
H	3.745430000	2.829514000	0.360177000
H	2.282115000	3.059798000	-0.597565000
H	2.170762000	2.870678000	1.154632000
H	5.371512000	-0.327363000	1.092346000
H	5.524398000	-0.216554000	-0.648657000
H	5.349643000	1.266842000	0.312604000
H	-0.010155000	2.048942000	-0.032218000
H	-3.194202000	-2.077632000	1.128121000
H	-3.773351000	2.551820000	-0.698588000
H	-2.190578000	3.024364000	-0.073050000
H	-2.325538000	2.323371000	-1.686063000
H	-5.353098000	-0.650955000	-0.451498000
H	-5.357229000	-0.225192000	1.258990000
H	-5.314839000	1.048056000	0.030587000
H	-0.693395000	-1.433007000	1.322708000
H	-1.583977000	-0.196621000	2.054784000

[**21a** + H]$^+$
Sum of electronic and zero-point energies: -840.501497

C	-1.490313000	0.311147000	-0.064043000
N	-1.710349000	-0.980204000	-0.450904000
C	-3.113314000	-1.246247000	-0.461400000
C	-3.758480000	-0.013587000	0.088218000
C	-2.791363000	0.908386000	0.301706000
C	-0.279035000	0.965623000	-0.021256000
C	0.989850000	0.391539000	-0.218112000
C	2.229880000	1.150603000	-0.391293000
C	3.254246000	0.271163000	-0.471093000
C	2.712113000	-1.141689000	-0.384641000
N	1.266271000	-0.922272000	-0.265862000
C	-2.916800000	2.293757000	0.841298000
C	-5.224363000	0.037895000	0.308918000
C	2.266923000	2.643057000	-0.453242000
C	4.709075000	0.501065000	-0.632575000
O	3.224849000	-1.947502000	0.632009000
O	-3.605574000	-2.264781000	-0.856141000
O	3.057415000	-1.196661000	1.879557000
H	-1.089054000	-1.521275000	-1.035921000
H	-0.300211000	2.026802000	0.179065000
H	2.938658000	-1.720135000	-1.287799000
H	0.686312000	-1.629108000	0.165853000
H	-2.548950000	3.033010000	0.123558000
H	-3.955288000	2.533946000	1.062477000
H	-2.340793000	2.410772000	1.763679000
H	-5.753472000	-0.223639000	-0.611814000
H	-5.520874000	-0.704755000	1.056253000
H	-5.555061000	1.020389000	0.641694000
H	1.634140000	3.020287000	-1.260978000
H	1.913702000	3.085446000	0.482799000
H	3.279435000	3.003368000	-0.626941000
H	5.065354000	0.078831000	-1.578444000
H	4.962809000	1.559349000	-0.610951000
H	5.255247000	-0.009695000	0.166417000
H	3.492547000	-1.810992000	2.490108000

[**21b** + H]$^+$

Sum of electronic and zero-point energies: -840.472935

C	-1.154355000	0.319869000	0.072982000
N	-1.422069000	-0.977257000	0.472651000
C	-2.792837000	-1.275005000	0.279385000
C	-3.424779000	0.001338000	-0.163013000
C	-2.454017000	0.930358000	-0.303228000
C	0.026920000	0.972966000	0.039887000
C	1.378230000	0.507943000	0.485195000
C	2.285454000	0.194155000	-0.704906000
C	2.667413000	-1.116438000	-0.657479000
C	2.131502000	-1.657539000	0.556765000
N	1.381684000	-0.795921000	1.194584000
C	-2.580057000	2.358273000	-0.717157000
C	-4.893817000	0.085132000	-0.371883000
C	2.620747000	1.239683000	-1.694401000
C	3.508380000	-1.897864000	-1.617418000
O	-3.282231000	-2.362502000	0.442491000
H	-0.753908000	-1.728617000	0.490139000
H	0.043904000	1.978401000	-0.357488000
H	0.990066000	-0.913596000	2.121219000
H	-1.989109000	2.565836000	-1.614438000
H	-3.615968000	2.611495000	-0.936412000
H	-2.230369000	3.029915000	0.072361000
H	-5.223526000	-0.702622000	-1.054490000
H	-5.422085000	-0.081056000	0.571747000
H	-5.196719000	1.050794000	-0.774366000
H	1.754944000	1.458741000	-2.328862000
H	2.890953000	2.167147000	-1.182067000
H	3.441184000	0.931708000	-2.340606000
H	2.888170000	-2.528921000	-2.260235000
H	4.087633000	-1.236296000	-2.260511000
H	4.207398000	-2.547949000	-1.086977000
H	2.294547000	-2.656342000	0.946640000
O	1.855987000	1.527151000	1.323295000
O	3.271362000	1.229632000	1.585816000
H	3.441948000	1.882142000	2.282507000

[**16b** + H]$^+$
Sum of electronic and zero-point energies: -840.592056

C	0.925824000	-0.616883000	-0.229617000
N	1.005362000	0.801374000	-0.328651000
C	2.254196000	1.173799000	-0.050766000
C	3.088890000	0.036965000	0.234593000
C	2.278941000	-1.062854000	0.125725000
C	-0.156341000	-1.392164000	-0.407630000
C	-1.576812000	-0.973508000	-0.731399000
C	-2.306881000	-0.590299000	0.572198000
C	-2.507922000	0.738023000	0.601393000
C	-1.889476000	1.322005000	-0.622500000
N	-1.619086000	0.270757000	-1.475432000
C	2.647170000	-2.492352000	0.319167000
C	4.538949000	0.155902000	0.566995000
C	-2.651942000	-1.638877000	1.570302000
C	-3.138028000	1.606805000	1.633779000
O	-1.522093000	2.473548000	-0.805229000
O	2.689244000	2.405254000	-0.035107000
O	-2.135575000	-2.063623000	-1.415867000
H	0.248037000	1.451403000	-0.536814000
H	-0.024805000	-2.460483000	-0.280370000
H	-1.050516000	0.407707000	-2.300699000
H	2.070548000	-2.937076000	1.135205000
H	2.442484000	-3.073667000	-0.584024000
H	3.703612000	-2.599583000	0.557230000
H	4.983569000	-0.821764000	0.744110000
H	5.087005000	0.638206000	-0.246498000
H	4.684843000	0.764917000	1.462873000
H	-3.189839000	-2.463078000	1.094314000
H	-1.749813000	-2.067662000	2.020126000
H	-3.269785000	-1.236669000	2.371895000
H	-3.361301000	1.057516000	2.547590000
H	-2.478026000	2.444139000	1.874978000
H	-4.067503000	2.041333000	1.254436000
H	-3.036960000	-1.837597000	-1.680098000
H	2.012498000	3.066640000	-0.249495000

[**20’** + D]$^+$

C	1.278833000	0.375587000	-0.065480000
N	1.511143000	-0.988610000	-0.122864000
C	2.914843000	-1.223526000	-0.127986000
C	3.554152000	0.112608000	0.076261000
C	2.581902000	1.052274000	0.092323000
C	2.703447000	2.529671000	0.261755000
C	5.027408000	0.231023000	0.216614000
O	3.425939000	-2.299384000	-0.280105000
C	0.059323000	0.972836000	-0.072813000
C	-1.191183000	0.222282000	-0.071156000
C	-2.543328000	0.906550000	-0.103462000
C	-3.461162000	0.003644000	0.285711000
C	-2.704654000	-1.271612000	0.584781000
C	-2.720794000	2.274438000	-0.667045000
C	-4.949275000	0.053732000	0.282848000
N	-1.440366000	-0.742166000	1.206959000
O	-1.170706000	-0.825525000	-1.056535000
O	-2.173617000	-1.826283000	-0.616164000
H(iso=2)	0.907055000	-1.608129000	-0.647652000
H	3.745430000	2.829514000	0.360177000
H	2.282115000	3.059798000	-0.597565000
H	2.170762000	2.870678000	1.154632000
H	5.371512000	-0.327363000	1.092346000
H	5.524398000	-0.216554000	-0.648657000
H	5.349643000	1.266842000	0.312604000
H	-0.010155000	2.048942000	-0.032218000
H	-3.194202000	-2.077632000	1.128121000
H	-3.773351000	2.551820000	-0.698588000
H	-2.190578000	3.024364000	-0.073050000
H	-2.325538000	2.323371000	-1.686063000
H	-5.353098000	-0.650955000	-0.451498000
H	-5.357229000	-0.225192000	1.258990000
H	-5.314839000	1.048056000	0.030587000
H(iso=2)	-0.693395000	-1.433007000	1.322708000
H(iso=2)	-1.583977000	-0.196621000	2.054784000

[**21a’** + D]$^+$

C	-1.490313000	0.311147000	-0.064043000
N	-1.710349000	-0.980204000	-0.450904000
C	-3.113314000	-1.246247000	-0.461400000
C	-3.758480000	-0.013587000	0.088218000
C	-2.791363000	0.908386000	0.301706000
C	-0.279035000	0.965623000	-0.021256000
C	0.989850000	0.391539000	-0.218112000
C	2.229880000	1.150603000	-0.391293000
C	3.254246000	0.271163000	-0.471093000
C	2.712113000	-1.141689000	-0.384641000
N	1.266271000	-0.922272000	-0.265862000
C	-2.916800000	2.293757000	0.841298000
C	-5.224363000	0.037895000	0.308918000
C	2.266923000	2.643057000	-0.453242000
C	4.709075000	0.501065000	-0.632575000
O	3.224849000	-1.947502000	0.632009000
O	-3.605574000	-2.264781000	-0.856141000
O	3.057415000	-1.196661000	1.879557000
H(iso=2)	-1.089054000	-1.521275000	-1.035921000
H	-0.300211000	2.026802000	0.179065000
H	2.938658000	-1.720135000	-1.287799000
H(iso=2)	0.686312000	-1.629108000	0.165853000
H	-2.548950000	3.033010000	0.123558000
H	-3.955288000	2.533946000	1.062477000
H	-2.340793000	2.410772000	1.763679000
H	-5.753472000	-0.223639000	-0.611814000
H	-5.520874000	-0.704755000	1.056253000
H	-5.555061000	1.020389000	0.641694000
H	1.634140000	3.020287000	-1.260978000
H	1.913702000	3.085446000	0.482799000
H	3.279435000	3.003368000	-0.626941000
H	5.065354000	0.078831000	-1.578444000
H	4.962809000	1.559349000	-0.610951000
H	5.255247000	-0.009695000	0.166417000
H(iso=2)	3.492547000	-1.810992000	2.490108000

[**21b’** + D]$^+$

C	-1.154355000	-0.319869000	-0.072982000
N	-1.422069000	0.977257000	-0.472651000
C	-2.792837000	1.275005000	-0.279385000
C	-3.424779000	-0.001338000	0.163013000
C	-2.454017000	-0.930358000	0.303228000
C	0.026920000	-0.972966000	-0.039887000
C	1.378230000	-0.507943000	-0.485195000
C	2.285454000	-0.194155000	0.704906000
C	2.667413000	1.116438000	0.657479000
C	2.131502000	1.657539000	-0.556765000
N	1.381684000	0.795921000	-1.194584000
C	-2.580057000	-2.358273000	0.717157000
C	-4.893817000	-0.085132000	0.371883000
C	2.620747000	-1.239683000	1.694401000
C	3.508380000	1.897864000	1.617418000
O	-3.282231000	2.362502000	-0.442491000
H(iso=2)	-0.753908000	1.728617000	-0.490139000
H	0.043904000	-1.978401000	0.357488000
H(iso=2)	0.990066000	0.913596000	-2.121219000
H	-1.989109000	-2.565836000	1.614438000
H	-3.615968000	-2.611495000	0.936412000
H	-2.230369000	-3.029915000	-0.072361000
H	-5.223526000	0.702622000	1.054490000
H	-5.422085000	0.081056000	-0.571747000
H	-5.196719000	-1.050794000	0.774366000
H	1.754944000	-1.458741000	2.328862000
H	2.890953000	-2.167147000	1.182067000
H	3.441184000	-0.931708000	2.340606000
H	2.888170000	2.528921000	2.260235000
H	4.087633000	1.236296000	2.260511000
H	4.207398000	2.547949000	1.086977000
H	2.294547000	2.656342000	-0.946640000
O	1.855987000	-1.527151000	-1.323295000
O	3.271362000	-1.229632000	-1.585816000
H(iso=2)	3.441948000	-1.882142000	-2.282507000

Calculated geometries with B3LYP-6–311++G(d,p) (solvent=methanol)

***Z*-13**

Sum of electronic and zero-point energies: -689.951998

C	1.02390800	0.27644100	-0.01376400
C	-0.21963700	0.81516100	-0.08248200
C	-1.48084100	0.12750700	-0.12171100
H	-0.27460500	1.89634600	-0.11741600
N	-1.61139400	-1.21347200	-0.46013600
C	-2.76610400	0.61105200	0.13081300
C	-3.68262700	-0.46278600	-0.08007600
C	-2.93910800	-1.55956800	-0.46337000
H	-0.88255800	-1.75158200	-0.90170400
C	-3.13541900	1.99045500	0.58468600
C	-5.17064400	-0.41093100	0.09719600
H	-3.25515100	-2.55510200	-0.73366000
C	2.30326700	0.98129200	-0.12123700
C	2.72040400	-1.25780200	0.23837600
C	3.31335300	0.07960000	-0.00098100
C	2.40419300	2.45395800	-0.37144100
C	4.79337500	0.26050500	-0.05462000
O	3.27865600	-2.30947100	0.49527300
N	1.33391000	-1.08719000	0.13093200
H	0.72378600	-1.73497100	0.60831800
H	-5.62558900	0.35793100	-0.53615300
H	-5.44808000	-0.18002600	1.13137100
H	-5.63084000	-1.36703200	-0.16191400
H	-2.28355300	2.67245500	0.55515600
H	-3.51319700	1.98875500	1.61351800
H	-3.92346500	2.41765800	-0.04391900
H	2.00146500	2.71733800	-1.35461400
H	3.44020700	2.79123500	-0.33437400
H	1.84054400	3.02326400	0.37403100
H	5.26396700	-0.18070600	0.82879700
H	5.07780700	1.31215700	-0.11190100
H	5.21769300	-0.25661900	-0.92163300

16a

Sum of electronic and zero-point energies: -879.764748

C	1.58093200	0.50780800	2.28214000
C	2.05017900	0.10216700	0.92529500
C	3.21297400	-0.44669500	0.54371500
C	4.41197500	-0.84659300	1.33773000
C	3.18318900	-0.65846100	-0.93974100
N	1.99867300	-0.10358500	-1.38755300
C	1.12950900	0.30592400	-0.29456200
O	0.69265400	1.65833900	-0.42360900
C	1.71438600	2.64365900	-0.58774400
C	-0.08882000	-0.58355000	-0.21250400
C	-1.38809500	-0.24157800	-0.12273500
N	-1.94239800	1.03206400	-0.09289100
C	-3.32409300	0.98080000	-0.01014700
C	-3.67116600	-0.47420300	0.03673500
C	-5.09290300	-0.91053200	0.14170700
C	-2.52645200	-1.18885800	-0.03246300
C	-2.35002400	-2.67446500	-0.02385900
O	-4.07706700	1.93550800	0.01746600
O	4.01819000	-1.21431200	-1.62506000
H	0.66813900	-0.03189300	2.55395500
H	1.32941700	1.57317000	2.30526900
H	2.33608100	0.31586800	3.04489400
H	5.28639800	-0.26209200	1.03551300
H	1.62755700	-0.35409200	-2.29270600
H	1.19493700	3.59344600	-0.71002800
H	2.31913500	2.44044000	-1.47450500
H	2.36724000	2.69879800	0.28932500
H	0.15127300	-1.64005800	-0.21111900
H	-1.39364900	1.87213600	-0.18834000
H	-5.55068300	-0.51147800	1.05208600
H	-5.19116700	-1.99675200	0.15180500
H	-3.31069800	-3.18513500	0.04421400
H	-1.73850000	-2.99801800	0.82437500
H	-1.85103700	-3.02086300	-0.93434400
H	4.25822800	-0.71162500	2.40900300
H	4.66080300	-1.89434600	1.14687900
H	-5.67717000	-0.51568000	-0.69494000

21a

Sum of electronic and zero-point energies: -801.003032

C	-3.37299300	-0.20887000	0.02753000
C	-2.58511300	0.88637300	0.10457900
C	-1.17603400	0.44639500	-0.00793600
C	-0.06003400	1.22321200	0.01460500
C	-4.85455800	-0.35628900	0.08140800
C	-2.49103000	-1.41124100	-0.14075000
C	-2.97739000	2.31960600	0.27366800
H	-4.06017800	2.42870400	0.33624700
H	-2.62709600	2.92767200	-0.56634000
H	-2.54430700	2.74337800	1.18519900
H	-5.35894700	0.60272500	0.20487700
H	-5.14466900	-1.01167200	0.90831400
H	-5.22601900	-0.83035600	-0.83221900
N	-1.18811300	-0.92105700	-0.14794700
C	1.28606400	0.70858000	-0.10975700
H	-0.18629900	2.29196300	0.12319300
O	-2.81938700	-2.57349900	-0.24820700
H	-0.32994100	-1.45338100	-0.25451000
N	1.57824500	-0.55560000	-0.22453200
C	2.50301100	1.58427100	-0.11284100
C	3.55130800	0.76075300	-0.23893800
C	3.02818600	-0.64288000	-0.38316100
C	2.48858200	3.07451700	0.01167000
H	2.01240800	3.38803600	0.94647300
H	1.92809700	3.53652400	-0.80771600
H	3.50201100	3.47833800	-0.00224200
H	4.60061400	1.01774600	-0.27081200
O	3.63869500	-1.46786900	0.59658900
H	3.24124600	-1.05922500	-1.37764700
O	3.30135500	-2.84455900	0.29342400
H	2.45685700	-2.94300000	0.75964000

S5a-I

Sum of electronic and zero-point energies: -879.747815

C	-4.07687800	0.07108000	-0.02064900
C	-3.10656100	1.00327900	-0.17913300
C	-1.79392600	0.34621300	-0.04788700
C	-0.58326500	0.95288500	-0.11318200
C	-5.56224300	0.18643600	-0.07537700
C	-3.42767700	-1.24088000	0.25358100
C	-3.25854200	2.46280200	-0.47249300
H	-4.30891000	2.75299300	-0.49543100
H	-2.81843600	2.72029400	-1.44085500
H	-2.75976000	3.07486400	0.28544800
H	-5.89120200	1.21946000	-0.19382300
H	-6.00980900	-0.22146700	0.83554500
H	-5.96457700	-0.40124700	-0.90676700
N	-2.05311700	-1.01178900	0.15149500
C	0.71849900	0.32713000	-0.07604700
H	-0.58717100	2.03128700	-0.19992200
O	-3.94671300	-2.30496200	0.52493600
H	-1.38742900	-1.66576800	0.53520600
N	0.87061800	-1.05568900	-0.39806300
C	1.91046100	0.87479300	0.25905600
C	2.99363100	-0.18974300	0.15479100
C	2.20862800	-1.40765300	-0.41623700
H	0.22872900	-1.53672400	-1.01374100
O	2.66694000	-2.45782800	-0.79160000
C	2.17715300	2.27762500	0.69666000
C	3.58302800	-0.59673500	1.51209000
H	1.26040500	2.86526100	0.76644000
H	2.84743600	2.78773700	-0.00376900
H	2.65753200	2.30887100	1.68146900
H	4.11019500	0.23067300	1.99101600
H	2.77984000	-0.91392300	2.18125200
H	4.27066200	-1.43525700	1.38076000
O	3.97432400	0.12561400	-0.83824000
C	5.17927400	0.76036800	-0.43541400
H	5.72489800	0.96120800	-1.35766300
H	5.79464200	0.11467200	0.19969100
H	5.00454400	1.71122800	0.08178600

S5b-II

Sum of electronic and zero-point energies: -840.425892

C	-3.69856000	-0.07520500	-0.00285300
C	-2.83206600	0.96100500	0.02677900
C	-1.45821800	0.40964800	0.00492400
C	-0.28883900	1.10239500	0.02301100
C	-5.18820200	-0.10812300	0.00146500
C	-2.90594800	-1.34941700	-0.04582400
C	-3.11945200	2.42811700	0.07390700
H	-4.19242800	2.62092400	0.08421000
H	-2.69463300	2.94275600	-0.79376300
H	-2.68755000	2.88791600	0.96846300
H	-5.62188500	0.89225300	0.03202500
H	-5.55686800	-0.67401800	0.86252800
H	-5.56266200	-0.62317700	-0.88847000
N	-1.57123600	-0.96047900	-0.03729400
C	1.02034400	0.48140800	-0.00158300
H	-0.33851100	2.18276700	0.05293800
O	-3.32390100	-2.48800200	-0.07972300
H	-0.74933500	-1.55906700	-0.06806100
N	1.19624300	-0.81033200	-0.02286700
C	2.28397500	1.26391900	-0.00795900
C	3.24862300	0.32962100	-0.02699300
C	2.63165900	-1.06111700	-0.00489500
C	2.40722600	2.75573800	-0.02557200
H	1.93850700	3.21293900	0.85202900
H	1.93662600	3.19086400	-0.91344700
H	3.45412500	3.07686400	-0.02916500
C	3.01687000	-1.84613900	1.25390200
H	2.71306300	-1.30757800	2.15431900
H	4.09898600	-2.00233500	1.28959500
H	2.51249200	-2.81287300	1.22953700
O	4.59650900	0.42244900	-0.07041400
H	4.87255000	1.34595500	-0.11503600
O	2.96190200	-1.79055900	-1.18594900
H	3.92192000	-1.88072600	-1.21773300

References

(1) Madea, D.; Mujawar, T.; Dvořák, A.; Pospíšilová, K.; Muchová, L.; Čubáková, P.; Kloz, M.; Švenda, J.; Vítek, L.; Klán, P. Photochemistry of (Z)-Isovinylneoxanthobilirubic Acid Methyl Ester, a Bilirubin Dipyrrinone Subunit: Femtosecond Transient Absorption and Stimulated Raman Emission Spectroscopy. *J. Org. Chem.* **2022**, *87,* 3089-3103.

(2) Bonnett, R.; Hamzetash, D.; Vallés, M. A. Propentdyopents [5-(2-Oxo-2*H*-Pyrrol-5-ylmethylene)pyrrol-2(5*H*)-Ones] and Related Compounds. Part 2. The *Z*⇄*E* Photoisomerisation of Pyrromethenone Systems. *J. Chem. Soc., Perkin Trans. 1* **1987**, 1383-1388.

(3) Madea, D.; Mahvidi, S.; Chalupa, D.; Mujawar, T.; Dvořák, A.; Muchová, L.; Janoš, J.; Slavíček, P.; Švenda, J.; Vítek, L.; Klán, P. Wavelength-Dependent Photochemistry and Biological Relevance of a Bilirubin Dipyrrinone Subunit. *J. Org. Chem.* **2020**, *85*, 13015-13028.

(4) Frisch, M. J.; Trucks, G. W.; Schlegel, H. B.; Scuseria, G. E.; Robb, M. A.; Cheeseman, J. R.; Scalmani, G.; Barone, V.; Petersson, G. A.; Nakatsuji, H.; et al. *Gaussian 16 Rev. C.01*; Wallingford, CT, 2016.

(5) Bielski, B. H. J.; Shiue, G. G.; Bajuk, S. Reduction of Nitro Blue Tetrazolium by CO2- and O2- Radicals. *J. Phys. Chem.* **1980**, *84*, 830-833.

(6) Saito, I.; Matsuura, T.; Inoue, K. Formation of superoxide ion via one-electron transfer from electron donors to singlet oxygen. *J. Am. Chem. Soc.* **1983**, *105*, 3200-3206.

(7) Inoue, K.; Matsuura, T.; Saito, I. Importance of single electron-transfer in singlet oxygen reaction in aqueous solution: Oxidation of electron-rich thioanisoles. *Tetrahedron* **1985**, *41*, 2177-2181.

(8) Falk, H.; Leodolter, A.; Schade, G. Beiträge zur Chemie der Pyrrolpigmente, 19. Mitt.: Die elektrochemische Oxidation von Pyrromethenonen und Pyrromethenen (Gallenpigment-Partialstrukturen). *Monatsh. Chem.* **1978**, *109*, 183-192.

(9) Koppenol, W. H.; Stanbury, D. M.; Bounds, P. L. Electrode potentials of partially reduced oxygen species, from dioxygen to water. *Free. Radic. Biol. Med.* **2010**, *49*, 317-322.

(10) Whittaker, E. T. On a New Method of Graduation. *Proc. Edinb. Math. Soc.* **1922**, *41*, 63-75.

(11) Virtanen, P.; Gommers, R.; Oliphant, T. E.; Haberland, M.; Reddy, T.; Cournapeau, D.; Burovski, E.; Peterson, P.; Weckesser, W.; Bright, J.; et al. Scipy 1.0: Fundamental Algorithms for Scientific Computing in Python. *Nat. Methods* **2020**, *17*, 261-272.

(12) Newville, M.; Otten, R.; Nelson, A.; Stensitzki, T.; Ingargiola, A.; Allan, D.; Fox, A.; Carter, F.; Michał; Osborn, R.; et al. *LMFIT: Non-Linear Least-Square Minimization and Curve-Fitting for Python*; Zenodo: 2024.

(13) Bregnhoj, M.; Westberg, M.; Jensen, F.; Ogilby, P. R. Solvent-dependent singlet oxygen lifetimes: temperature effects implicate tunneling and charge-transfer interactions. *Phys. Chem. Chem. Phys.* **2016**, *18*, 22946-22961.

(14) Redmond, R. W.; Gamlin, J. N. A compilation of singlet oxygen yields from biologically relevant molecules. *Photochem. Photobiol.* **1999**, 391-475.

(15) van Stokkum, I. H. M.; Larsen, D. S.; van Grondelle, R. Global and Target Analysis of Time-Resolved Spectra. *Biochim. Biophys. Acta, Bioenerg.* **2004**, *1657*, 82-104.

(16) Galliani, G.; Manitto, P.; Monti, D. A Kinetic-Study of the Interaction between Bilirubin and Thermally Produced Singlet Oxygen. *Isr. J. Chem.* **1983**, *23*, 219-222.
